# Copper(I) Iodide Catalyzed [3 + 3] Annulation of Iodonium Ylides with Pyridinium 1,4-Zwitterionic Thiolates for the Synthesis of 1,4-Oxathiin Scaffolds

**DOI:** 10.1021/acs.orglett.3c01538

**Published:** 2023-06-26

**Authors:** Àlex Díaz-Jiménez, Stuart C. D. Kennington, Anna Roglans, Anna Pla-Quintana

**Affiliations:** Institut de Química Computacional i Catàlisi and Departament de Química, Universitat de Girona, C/Maria Aurèlia Capmany, 69, 17003 Girona, Catalunya Spain

## Abstract

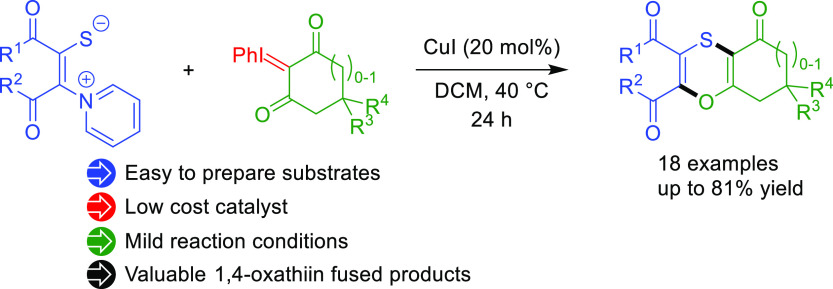

The selective assembly of the 1,4-oxathiin nucleus has been treated as a powerful strategy to access this scaffold present in molecules with very interesting properties. In this study, the chameleon-like reactivity of pyridinium 1,4-zwitterionic thiolates is exploited to assemble the 1,4-oxathiin core through a [3 + 3] annulation. The optimal annulation partner has been found to be the iodonium ylide of the cyclic 1,3-diketones. The developed protocol allows the synthesis of a variety of bicyclic 1,4-oxathiin derivatives under very mild conditions under copper(I) iodide catalysis. Access to benzoannulated 1,4-oxathiins has been achieved through iodine-mediated aromatization of the initially obtained bicyclic compounds.

The development of novel methodologies for the preparation of sulfur-containing heterocyclic compounds has become a key objective for the synthetic community as these sulfur-based molecules are present in pharmaceutically active molecules,^1a^ natural products,^1b^ and various functional materials.^1c^ Specifically, 1,4-oxathiin containing molecules have drawn great attention as they have a wide array of potential applications, such as fungicides or pesticides,^2a^ antiviral,^2b^ bioimaging,^2c^ anticancer drugs,^2d^ and even artificial sweetening agents.^2e^

While several methodologies for the preparation of the benzoannulated derivatives of 1,4-oxathiins have been reported,^3^ examples regarding the synthesis of simple 1,4-oxathiins are scarce. A couple of examples report their preparation from ring expansions of 1,3-oxathiolanes,^4^ and more recently Khan et al.^2d^ utilized copper catalysis to forge this core by the reaction of 4-hydroxydithiocoumarins, arylacetylenes, and DMSO. The synthesis of the 5,6-dihydrogenated-1,4-oxathiin core is a bit more developed and can be for instance accomplished by [4 + 2] annulation as reported by Nishino et al.^5^ and Ye et al.^6^ or ring expansion of thiiranes with copper carbenes as reported by Xu et al.^7^ Nonetheless, given the low quantity of methodologies reported so far, the development of novel transformations for the preparation of these heterocycle derivatives is highly desirable.

Pyridinium 1,4-zwitterionic thiolates (PZTs)^8^ can be regarded as air-stable, odorless and easy-to-handle sulfur containing synthons. Depending on the reaction partner, PZTs can engage as 1,3-dipoles in [3+m] annulations if the pyridine moiety acts as a leaving group or as 1,5-dipoles in [5+m] annulations if the pyridine unit remains in the final product. This versatility has accounted for the preparation of a myriad of different sulfur or sulfur and nitrogen containing scaffolds.^9^ In this context, we have recently reported the use of predictive catalysis as a tool for designing a novel reaction in which a copper carbene can be trapped with PZTs, furnishing dihydropyridothiazines through a [5 + 1] annulation, which upon oxidation with DDQ and sulfur extrusion, afford indolizine derivatives with high yields (Scheme 1a).^10^ Xu et al. also recently reported that diazo(aryl)methyl(diaryl)phosphine oxides react with PZTs to give a [5 + 1] annulation product under blue light irradiation,^11a^ but interestingly, benzo[c]thiopyran scaffolds were obtained via [3 + 3] annulation when the reaction was carried out under microwave irradiation (Scheme 1b).^11b^ The authors postulate that the [3 + 3] annulation reaction capitalizes from an intramolecular Michael addition of an electron-rich phenyl group in the zwitterionic intermediate formed upon nucleophilic attack of the PZT to the carbene.

**Scheme 1:**
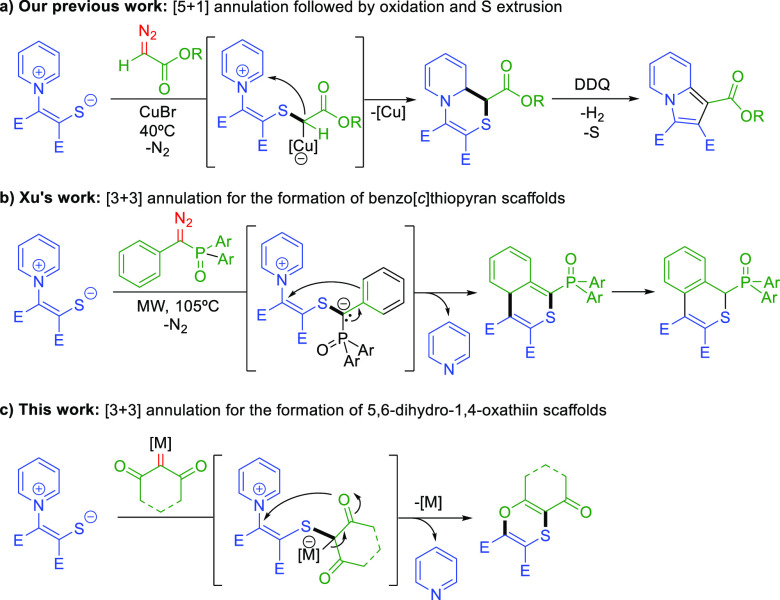
Different Reaction Modes of PZTs and Carbene or Metallacarbene Species (E = Ester Group)

The in-depth mechanistic information gained from our previous study prompted us to further explore the reactivity of PZTs in carbene chemistry. Inspired by the low abundance of methodologies available for the preparation of 1,4-oxathiins we envisioned that by modifying the α-oxo metal carbene and the reaction conditions, we could switch the chemoselectivity of the reaction and promote an *oxa-*Michael addition from the α-oxo carbene that triggers a [3 + 3] annulation toward a 1,4-oxathiin scaffold (Scheme 1c). We thus present here our efforts to achieve a copper catalyzed [3 + 3] annulation between pyridinium 1,4-zwitterionic thiolates and metal carbenes generated *in situ* from iodonium ylides.

Our study commenced with the reaction of PZT **1a** and 2-diazo-5,5-dimethylcyclohexane-1,3-dione (Scheme 2, Z = N_2_). This cyclic diazo dicarbonyl compound derived from dimedone^12^ was chosen because it had been previously described to efficiently engage as a 3-atom reagent in annulation reactions^13^ including [3 + 3] annulations.^14^ However, when the two compounds were mixed in dichloromethane (DCM) in the presence of 20 mol% of CuI and stirred at 40 °C for 24 h, only starting materials were recovered. Increasing the temperature to 80 °C in dichloroethane (DCE) resulted in the formation of a 36% yield of 1,4-oxathiin derivative **3aa**. With the aim to work at milder reaction conditions, we decided to test iodonium ylides as carbene precursors, which are bench-stable, nontoxic, and easy to prepare from the corresponding 1,3-dicarbonyl compounds.^15^ 2-(Phenyl-λ^3^-iodanylidene)cyclohexane-1,3-dione derivatives have also been tested as 3-atom synthons in [3 + 3] annulation reactions, especially in rhodium catalyzed cascades encompassing C–H activation with a directing group, carbene migratory insertion, and annulation.^16^ After some optimization with the iodonium ylide derived from 1,3-cyclohexandione (Scheme 2, Z = PhI) we were able to isolate a 56% yield of 1,4-oxathiin **3aa** at 40 °C (see the SI for details). It should be noted that a blank reaction without copper showed that the presence of the catalyst is crucial for the reaction to take place.

**Scheme 2:**
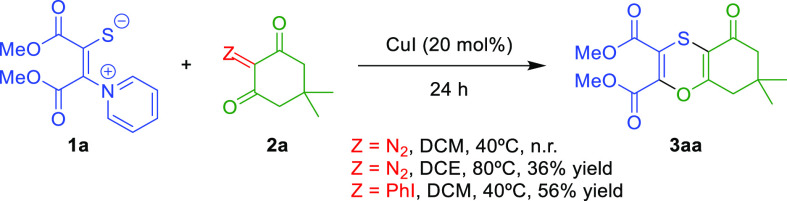
Optimization of the Carbene Precursor Substrate

After obtaining optimized conditions, we moved to investigate the scope of the reaction (Scheme 3). First, by varying the type of ester in the PZT starting material **1** we were able to synthesize a variety of products (**3aa–3ga**) with good tolerance of the differing ester groups (Scheme 3). Changing the esters from methyl to ethyl resulted in an increase in the yield (**3ba**). Further elongating the ester or adding branching reduced the yield to around 40% (**3ca–3ea**) most likely due to the increased steric interactions. The benzyl ester was also similarly tolerated with a comparable yield (**3fa**). Finally, the use of a PZT containing a methyl ester next to the pyridinium and a phenyl ketone next to the sulfur (**1g**), furnished product **3ga** in a 25% yield. Considering the added complexity and benefit of being able to obtain an unsymmetric ketone/ester instead of a diester this is a considerable achievement.

**Scheme 3:**
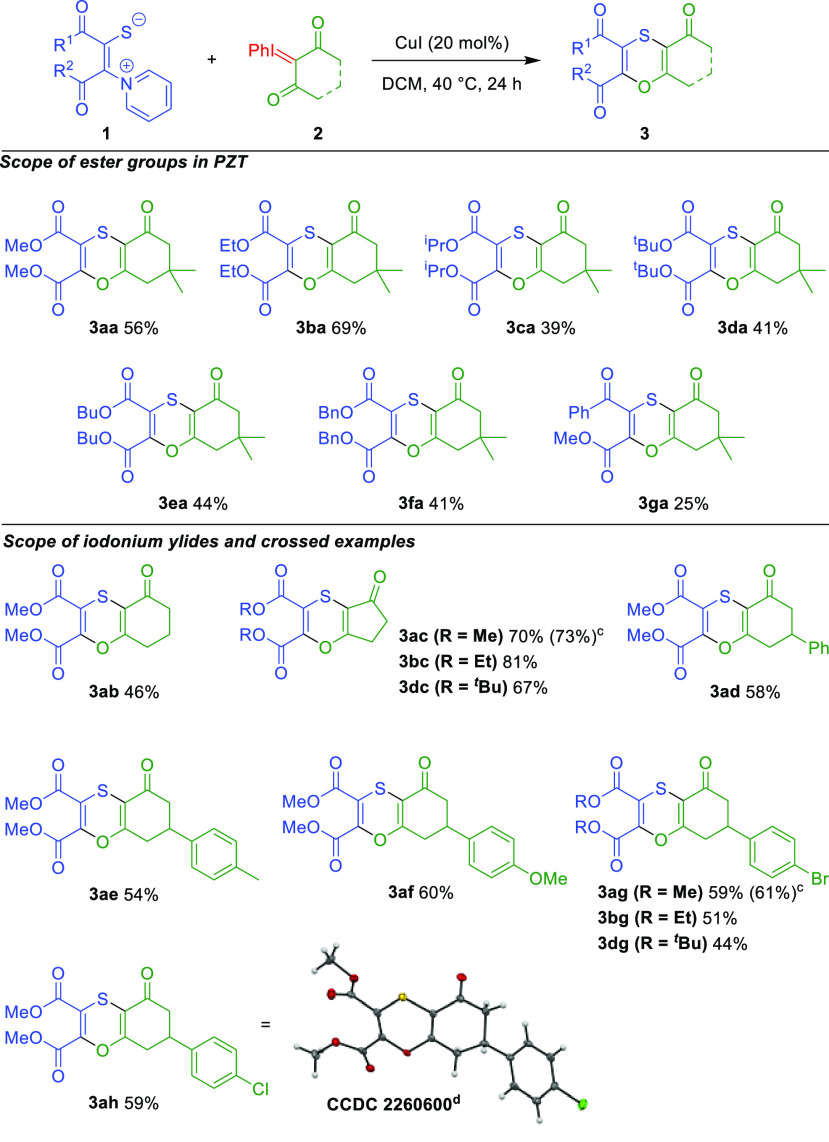
Scope of the Reaction^,^ Reaction conditions: **1** (0.1 mmol), **2** (0.3 mmol), CuI (0.02 mmol) in 3.2 mL of dichloromethane at 40 °C for 24 h. Isolated yields. Yield for the reaction at 1 mmol scale. ORTEP drawing at 50% ellipsoid probability.

After examining the effect of different PZT starting materials, we then moved to the complementary reagent: the iodonium carbene precursor. We examined a range of compounds prepared from cyclic 1,3-diketone analogues. We investigated the effect of the substitution on the ring and the size of the ring (Scheme 3). Using the iodonium prepared from unsubstituted 1,3-cyclohexanedione (**2b**) resulted in a moderate drop in the yield, highlighting the important role of the substitution in the 5 position. Using 2-(phenyl-λ^3^-iodanylidene)cyclopentane-1,3-dione **2c** the product **3ac** was formed in a 70% yield. Reaction of this iodonium ylide with PZT functionalized with different ester groups, such as ethyl and *tert*-butyl was also evaluated. The reaction with ethyl decorated PZT **1b**, gave an excellent 81% yield of the corresponding 1,4-oxathiin, whereas the reaction with bulkier *tert*-butyl ester substituted **1d** furnished **3dc** in a 67% yield. Iodonium ylides substituted with aromatic groups in the 5 position were then evaluated. Phenyl substituted substrate **2d** gave the product **3ad** in a similar yield to that of the dimethyl product **3aa**. Further substitution on the aromatic ring itself gave comparable yields (**3ae–3ah**) with tolerance for different substituents, both electron donating (Me, MeO) and electron withdrawing (Cl, Br), suggesting the ease of the preparation of a large variety of products by the simple variation of the aromatic group. The structure of **3ah** could be confirmed by single-crystal X-ray diffraction analysis (CCDC-2260600). For the 4-bromo substituted derivative **2g**, we again studied the effect of variation of the ester groups, resulting in similar yields for methyl and ethyl esters and a moderate decrease for the annulation with *tert*-butyl **PZT**. Finally, we scaled up the reaction to a 1 mmol scale for the synthesis of 1,4-oxathiin derivatives **3ac** and **3ag** which gave almost identical yields of 73% and 61%, respectively, proving the scalability of the reaction.

We then examined the possibility of using linear iodonium compounds (Scheme 4). The reaction of PZT **1a** with 3-(phenyl-λ^3^-iodanylidene)pentane-2,4-dione (**2i**) provided furan **3′ai** via formal [3 + 3] annulation followed by a spontaneous and unexpected ring-contraction/sulfur extrusion.^17^ It should be noted that a 24% yield of an indolizine was also isolated in this reaction, formed by [5 + 1] annulation followed by formal acetaldehyde elimination and sulfur extrusion, in contrast to all other reactions evaluated where this annulation, reported in our previous study,^10^ was never observed. Next, the reaction of the iodonium ylide derived from 1,3-diphenylpropane-1,3-dione (**2j**) was carried out to give an analogous furan **3′aj** in 52% yield together with unidentified products. On the other hand, the reaction with the linear iodonium ylide derived from diethyl malonate (**2k**) only led to decomposition products, hinting that ester derivatives are not suitable for the reaction.

**Scheme 4:**
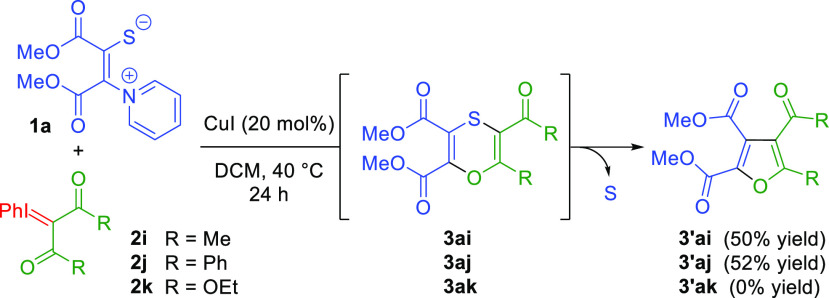
Reaction with Linear Iodonium Ylides

To show the practical application of the newly developed synthesis, we looked at functionalizing the products further (Scheme 5). We first performed ester hydrolysis, which under basic conditions provided the diacid **5ag** in a good yield. This acid was slightly unstable and degraded slowly under standard conditions. We then performed an aromatization reaction, using iodine and potassium carbonate as a base following a procedure previously described for the aromatization of isocoumarine scaffolds.^18^ Benzoannulated 1,4-oxathiin scaffold **4ag** was obtained in a good 65% yield. We then attempted an iodination reaction of the α-carbonyl position using NIS;^18^ however, we observed the subsequent aromatization leading to the same aromatic product **4ag** in a slightly lower yield.

**Scheme 5:**
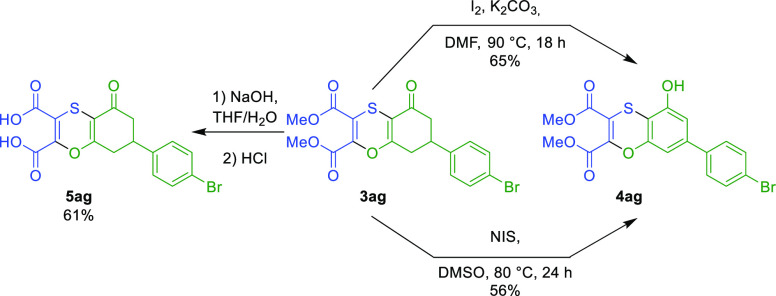
Further Functionalization of the 1,4-Oxathiin Derivatives

Based on our previous work and our experimental observations, we propose the mechanism shown in Scheme 6. First, extrusion of iodobenzene in the presence of the copper catalyst yields the copper carbene **I**. Then the pyridinium 1,4-thiolate **1** nucleophilically attacks carbene **I** to form the metal bound intermediate **II** or upon copper release metal unbound intermediate **III**. The *O*-enolate nucleophilically attacks, in a conjugate addition, the carbon attached to the pyridinium moiety, effectively displacing pyridine and closing the 1,4-oxathiin ring of **3**.

**Scheme 6:**
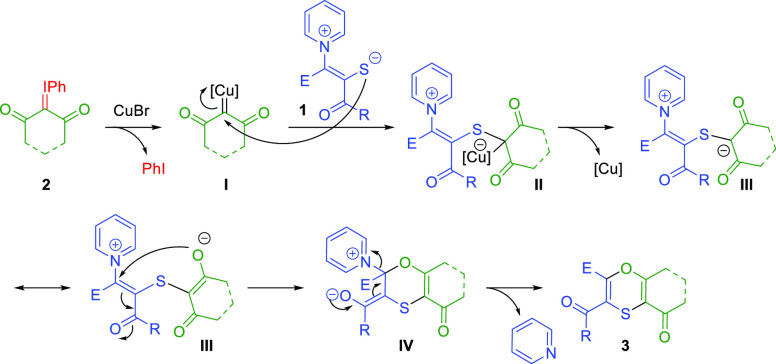
Proposed Mechanism for the Reaction of PZTs with Iodonium Ylides

One possible explanation for the change in selectivity seen using PZTs in copper catalyzed annulations, [3 + 3] in the present study vs [5 + 1] in our previous one (Scheme 1a),^10^ is the restricted geometry in intermediate **III** due to the large cyclic moiety causing an approach of the negatively charged oxygen to the *sp*^2^ carbon attached to the pyridinium ring providing easier access to the conjugate addition. This explanation is in line with the previous study by Xu et al.^11b^ reporting that an increase of the steric hindrance of the pyridinium ring of the PZT, favored the [3 + 3] annulation over the [5 + 1] annulation with phosphoryl carbenes. Furthermore, we also observe that the two annulations compete when **2j**, a more flexible (open-chain) and less sterically congested iodonium ylide, is reacted. However, the higher prevalence of an oxygen centered negative charge in intermediate **III** (Scheme 6) in comparison to the analogous intermediate in the reaction with acyl carbenes (Scheme 1a), could also explain a more favored conjugated attack of the harder negative oxygen that finally displaces the pyridine group.

In conclusion, we have successfully described a novel synthesis of bicyclic 5,6-dihydrogenated-1,4-oxathiin derivatives through a copper-catalyzed [3 + 3] annulation of PZT and iodonium ylides of cyclic 1,3-dicarbonyl compounds. Linear iodonium ylides afford furan derivatives through complementary ring contraction and sulfur extrusion. The method uses a simple, inexpensive, and stable copper catalyst and runs under mild conditions. The obtained compounds can be transformed to benzoannulated 1,4-oxathiin derivatives through an iodine mediated aromatization. The selectivity in the cyclization of the intermediate formed upon S attack to the carbene has been rationalized based on steric factors.

## Data Availability

The data underlying this study are available in the published article and its Supporting Information.

## Supporting Information Available

Experimental details and spectral data for all unknown compounds and crystallographic data (PDF)
