# Supplementary material for: Copper(I) Iodide Catalyzed [3 + 3] Annulation of Iodonium Ylides with Pyridinium 1,4-Zwitterionic Thiolates for the Synthesis of 1,4-Oxathiin Scaffolds
Source: Org Lett. 2023 Jun 26;25(26):4830–4. doi: 10.1021/acs.orglett.3c01538 (PMC10334467; doi:10.1021/acs.orglett.3c01538)

SUPPORTING INFORMATION

Copper(I) Iodide Catalyzed [3+3] Annulation of Iodonium Ylides with Pyridinium 1,4-Zwitterionic Thiolates for the Synthesis of 1,4-Oxathiin Scaffolds

Àlex Díaz-Jiménez,[a] Stuart C. D. Kennington,[a] Anna Roglans,[a] Anna Pla-Quintana*,[a]

[a] Institut de Química Computacional i Catàlisi (IQCC) and Departament de Química, Universitat de Girona (UdG), Facultat de Ciències, C/ Maria Aurèlia Capmany, 69, 17003-Girona, Catalunya, Spain.

CONTENTS

General materials and methods 3

S1. Optimization of the reaction conditions 5

S2. General procedure for the copper catalyzed [3+3] cycloaddition 6

S3. Further Functionalization 13

S4. Crystal structure of compound 3ah with probability level of 50 % 14

S5. References 18

S6. NMR SPECTRA 19

General materials and methods

Unless otherwise noted, materials were obtained from commercial suppliers and used without further purification. Pyridinium 1,4-zwitterionic thiolates [1-4] and iodonium ylides,[5,6] were prepared as previously reported in the literature. Reaction progress during the preparation of all compounds was monitored using thin layer chromatography on Macherey-Nagel Xtra SIL G/UV254 silica gel plates. Solvents were removed under reduced pressure with a rotary evaporator. Reaction mixtures were chromatographed on silica gel using an automated purification instrument Interchim PuriFlash XS 520 Plus equipped with a quaternary gradient pump (up to 300 ml/min, 20 bar) and an UV-Vis 200-800 nm diode array detector. All ^{1}H and ^{13}C NMR spectra were recorded on a Bruker ASCEND 400 spectrometer equipped with a 5 mm BBFO probe using acetone d_6 a deuterated solvent. Chemical shifts for ^{1}H and ^{13}C NMR are reported in ppm (δ) relative to residual solvent signals (Acetone d_6: 2.05 ppm for ^{1}H, 29.84 ppm for ^{13}C). Coupling constants are given in Hertz (Hz). Structural assignments were made with additional information from gHSQC, gHMBC and gCOSY experiments. Electrospray ionization high-resolution mass spectrometry was performed using a Bruker microTOF-Q II instrument operated in the positive ESI (+) ion mode. IR spectra were recorded on an Agilent Cary 630 FT-IR spectrometer equipped with an ATR sampling accessory. The X-ray intensity data were measured on a 'Bruker D8 QUEST ECO' three-circle diffractometer system equipped with a Ceramic x-ray tube (Mo Kα, λ = 0.71076 Å) and a doubly curved silicon crystal Bruker Triumph monochromator. Melting points were measured in a SMP10 apparatus from Stuart without any correction.

Unless otherwise noted, all materials were purchased from commercial suppliers and were used without further purification. Pyridinium 1,4-zwitterionic thiolate compounds **1a,**[1] **1b,**[1] **1c,**[2] **1d,**[1] **1e,**[3] **1f,**[4] and **1g,**[4] iodonium ylides **2a-2h**,[5] **2i-2k**[6] and diazo compound **2a'**[7] were prepared as previously reported in the literature.

Pyridinium 1,4-zwitterionic thiolates

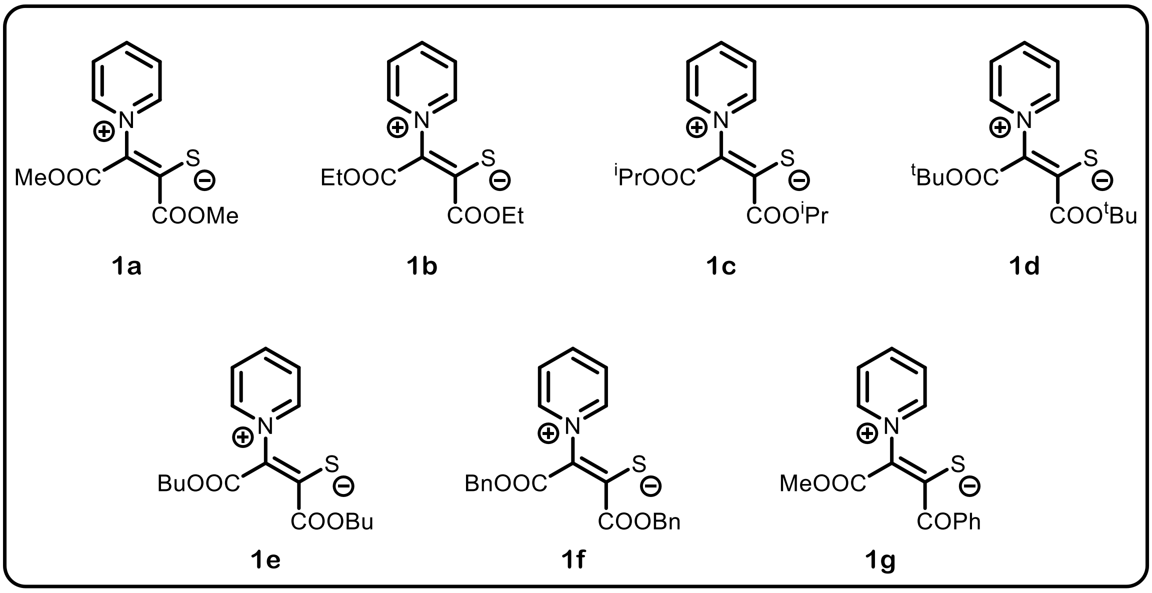

Carbene Precursors

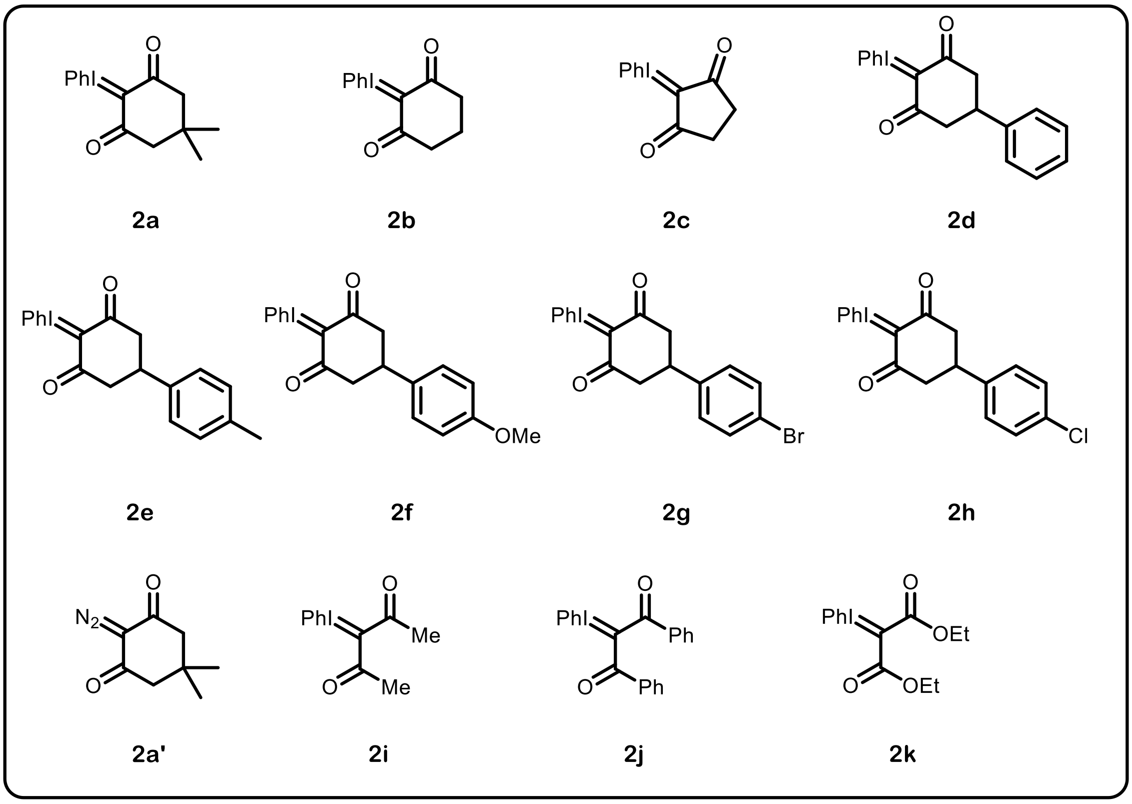

S1. Optimization of the reaction conditions

Catalyst optimization[a]

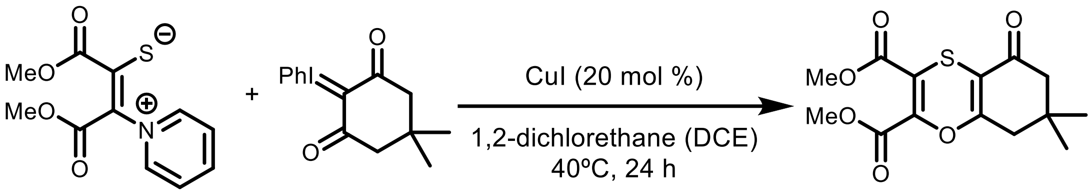

1a (3 equiv.) **2a** (1 equiv.) **3aa**

Entry	Deviation from standard conditions	NMR Yield (%)[a]
**1**	None	45
**2**	CuBr as the catalyst	31
**3**	$Cu(OTf)_2$ as the catalyst	27
**4**	CuCl as the catalyst	16
**5**	$CuCl_2$ as the catalyst	0
**6**	$Cu(AcN)_4PF_6$ as the catalyst	32
**7**	CuOAc as the catalyst	0
**8**	CuCN as the catalyst	28
**9**	$CuSO_4$·5H2O as the catalyst	43
**10**	CuO as the catalyst	34
**11**	No catalyst	0

[a] Standard conditions: Unless otherwise noted, reactions were carried out with 0.05 mmol of **2a** ([**2a**] = 0.03 mM) and 0.15 mmol of **1a**, at 40ºC (heating block) in 1.6 mL of 1,2-DCE for 24h. The yields given were determined by NMR using 1,3,5-trimethoxybenzene as internal standard.

Solvent optimization[a]

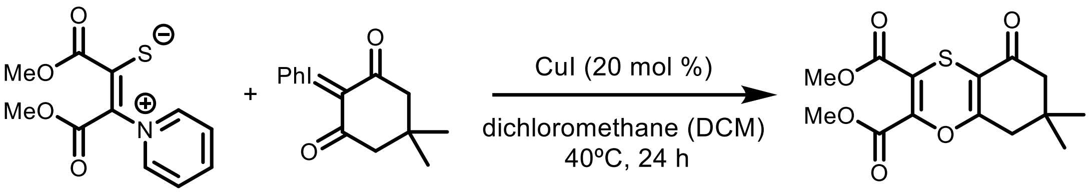

1a **2a** **3aa**

Entry	Deviation from standard conditions	NMR Yield (%)
**1**	None	61 (56)[b]
**2**	DCE	45
**3**	Acetone	31
**4**	Chlorobenzene	29
**5**	THF	17
**6**	Dioxane	16
**7**	Acetonitrile	32
**8**	Toluene	11
**9**	Chloroform	58

[a] Standard conditions: Unless otherwise noted, reactions were carried out with 0.05 mmol of **2a** ([**2a**] = 0.03 mM) and 0.15 mmol of **1a**, at 40ºC (heating block) in 1.6 mL of DCM for 24h. The

yields given were determined by NMR using 1,3,5-trimethoxybenzene as internal standard. [b] Isolated yield.

Equivalents optimization[a]

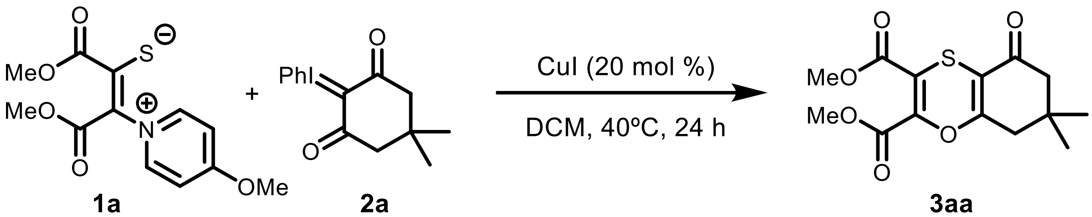

Entry	Deviation from standard conditions	NMR Yield (%)
**1**	None	61 (56)[b]
**2**	2.5 equivalents of **1a**	41
**3**	2.0 equivalents of **1a**	40
**4**	1.5 equivalents of **1a**	40
**5**	1.2 equivalents of **1a**	40

[a] Standard conditions: Unless otherwise noted, reactions were carried out with 0.05 mmol of **2a** ([**2a**] = 0.03 mM) and 0.15 mmol of **1a**, at 40ºC (heating block) in 1.6 mL of DCM for 24h. The yields given were determined by NMR using 1,3,5-trimethoxybenzene as internal standard. [b] Isolated yield.

S2. General procedure for the copper catalyzed [3+3] cycloaddition

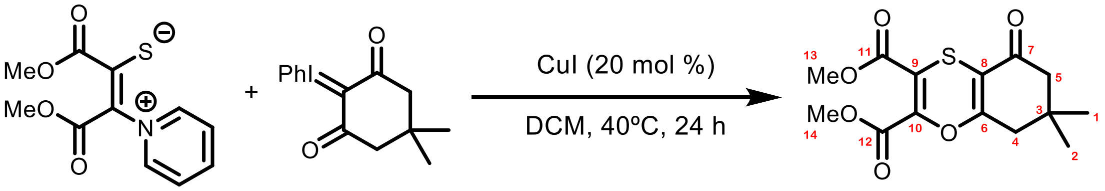

To an oven-dried Schlenk flask containing iodonium ylide **2a** (34.2 mg, 0.100 mmol), pyridinium 1,4-zwitterionic thiolate **1a** (75.9 mg, 0.299 mmol) and CuI (3.8 mg, 0.02 mmol) was added anhydrous dichloromethane (3.2 mL) under a nitrogen atmosphere. The mixture was heated to 40ºC (heating block) and stirred for 24h. The solvent was then removed under reduced pressure and the crude reaction mixture was purified by column chromatography on silica gel using hexane/EtOAc mixture as the eluent (100:0 to 80:20). Concentration under reduced pressure afforded compound **3aa** (17.5 mg, 56% yield) as a yellow solid.

MW ($C_{14}H_{16}O_6S$): 312.34 g/mol; **Rf:** 0.58 (Hexanes/EtOAc 6:4). **MP (ºC):** 93-95. **IR (ATR) ν (cm^{-1}):** 2956, 1741, 1684, 1598, 1249, 1035, 763. **^{1}H NMR (Acetone d_6, 400 MHz):** δ_H 3.80 (s, 3H, **$3H_{14}/3H_{13}$**), 3.79 (s, 3H, **$3H_{14}/3H_{13}$**), 2.41 (s, 2H, **$2H_4/2H_5$**), 2.38 (s, 2H, **$2H_4/2H_5$**), 1.11 (s, 6H, **$3H_1$** + **$3H_2$**). **^{13}C{H} NMR (Acetone d_6, 101 MHz):** δ_C 193.0 (**C_7**), 165.2 (**C_6**), 163.5 (**$C_{11/12}$**), 160.9 (**$C_{11/12}$**), 142.1 (**C_{10}**), 115.1 (**C_9**), 106.6 (**C_8**), 53.7 (**$C_{13/14}$**), 53.5 (**$C_{13/14}$**), 50.6 (**C_5**), 41.9 (**C_4**), 32.4 (**C_3**), 28.0 (**C_1** + **C_2**). **HRMS (ESI) m/z:** $[M+Na]^+$ calcd. for $C_{14}H_{16}O_6SNa$: 335.0560; Found 335.0562.

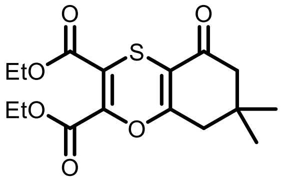

Starting from iodonium ylide **2a** (33.4 mg, 0.098 mmol) and pyridinium 1,4-zwitterionic thiolate **1b** (84.6 mg, 0.301 mmol), compound **3ba** was obtained as a yellow oil (22.8 mg, 69% yield).

MW ($C_{16}H_{20}O_6S$): 340.39 g/mol; **Rf:** 0.73 (Hexanes/EtOAc 6:4). **IR (ATR) ν (cm^{-1}):** 2958, 1720, 1650, 1603, 1367, 1248, 1049, 764. **^1H NMR (Acetone d_6, 400 MHz):** δ_H 4.30 – 4.19 (m, 4H), 2.41 (s, 2H), 2.38 (s, 2H), 1.33 – 1.23 (m, 6H), 1.11 (s, 6H). **$^{13}C\{H\}$ NMR (Acetone d_6, 101 MHz):** δ_C 193.1, 165.2, 162.9, 160.4, 142.5, 114.7, 106.8, 63.2, 63.1, 50.6, 41.9, 32.4, 28.0, 14.1. **HRMS (ESI) m/z:** $[M+Na]^+$ calcd. for $C_{16}H_{20}O_6SNa$ 363.0873; Found 363.0879.

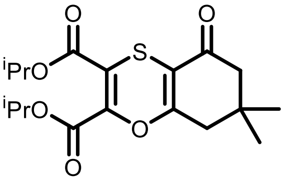

Starting from iodonium ylide **2a** (34.0 mg, 0.099 mmol) and pyridinium 1,4-zwitterionic thiolate **1c** (92.7 mg, 0.300 mmol), compound **3ca** was obtained as a yellow solid (14.1 mg, 39% yield).

MW ($C_{18}H_{24}O_6S$): 368.44 g/mol; **Rf:** 0.38 (Hexanes/EtOAc 9:1). **MP (ºC):** 82-85. **IR (ATR) ν (cm^{-1}):** 2956, 1721, 1648, 1604, 1257, 1129, 1098, 827. **^1H NMR (Acetone d_6, 400 MHz):** δ_H 5.12 – 4.99 (m, 2H), 2.41 (s, 2H), 2.38 (s, 2H), 1.29 (d, 6H, J = 4.9 Hz), 1.27 (d, 6H, J = 4.9 Hz) 1.11 (s, 6H).**$^{13}C\{H\}$ NMR (Acetone d_6, 101 MHz):** δ_C 193.1, 165.2, 162.3, 160.0, 142.7, 114.5, 106.9, 71.3, 71.2, 50.7, 42.0, 32.4, 28.0, 21.6. **HRMS (ESI) m/z:** $[M+Na]^+$ calcd. for $C_{18}H_{24}O_6SNa$: 391.1186; Found 391.1181.

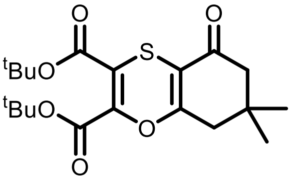

Starting from iodonium ylide **2a** (33.6 mg, 0.098 mmol) and pyridinium 1,4-zwitterionic thiolate **1d** (103.5 mg, 0.308 mmol), compound **3da** was obtained as a yellow solid (16.0 mg, 41% yield).

MW ($C_{20}H_{28}O_6S$): 396.50 g/mol; **Rf:** 0.60 (Hexanes/EtOAc 9:1). **MP (ºC):** 84-87. **IR (ATR) ν (cm^{-1}):** 2972, 1720, 1649, 1601, 1252, 1126, 1055, 842. **^1H NMR (Acetone d_6, 400 MHz):** δ_H 2.39 (s, 2H), 2.37 (s, 2H), 1.50 (s, 9H), 1.49 (s, 9H), 1.11 (s, 6H).**$^{13}C\{H\}$ NMR (Acetone d_6, 101 MHz):** δ_C 193.1, 165.3, 161.8, 159.8, 143.6, 114.2, 107.2, 84.3, 84.2, 50.7, 42.0, 32.4, 28.0, 27.96, 27.93. **HRMS (ESI) m/z:** $[M+Na]^+$ calcd. for $C_{20}H_{28}O_6SNa$: 419.1499; Found 419.1502.

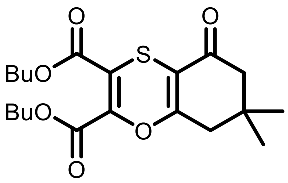

Starting from iodonium ylide **2a** (33.0 mg, 0.096 mmol) and pyridinium 1,4-zwitterionic thiolate **1e** (100.5 mg, 0.298 mmol), compound **3ea** was obtained as a yellow oil (16.8 mg, 44% yield).

MW ($C_{20}H_{28}O_6S$): 396.50 g/mol; **Rf:** 0.63 (Hexanes/EtOAc 9:1). **IR (ATR) ν (cm^{-1}):** 2955, 1727, 1651, 1604, 1347, 1248, 1122, 939. **^1H NMR (Acetone d_6, 400 MHz):** δ_H 4.25 – 4.16 (m, 4H), 2.41 (s, 2H), 2.38 (s, 2H), 1.70 – 1.60 (m, 4H), 1.46 – 1.34 (m, 4H), 1.11 (s, 6H), 0.98 – 0.90 (m, 6H). **$^{13}C\{H\}$ NMR (Acetone d_6, 101 MHz):** δ_C 193.1, 165.1, 162.9, 160.6, 142.5, 114.6, 106.8, 66.9, 66.8, 50.6, 41.9, 32.4, 31.1, 31.0, 28.0, 19.7, 13.9, 13.8. **HRMS (ESI) m/z:** $[M+Na]^+$ calcd. for $C_{20}H_{28}O_6SNa$: 419.1499; Found 419.1502.

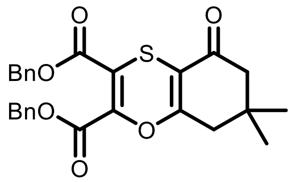

Starting from iodonium ylide **2a** (34.5 mg, 0.100 mmol) and pyridinium 1,4-zwitterionic thiolate **1f** (121.6 mg, 0.300 mmol), compound **3fa** was obtained as a yellow oil (19.1 mg, 41% yield).

MW ($C_{26}H_{24}O_6S$): 464.53 g/mol; **Rf:** 0.25 (Hexanes/EtOAc 9:1). **IR (ATR) ν (cm^{-1}):** 3030, 2955, 1720, 1649, 1601, 1347, 1246, 694. **^{1}H NMR (Acetone d_6, 400 MHz):** δ_H 7.44 – 7.33 (m, 10H), 5.16 (s, 2H), 5.15 (s, 2H), 2.41 (s, 2H), 2.38 (s, 2H), 1.11 (s, 6H). **^{13}C{H} NMR (Acetone d_6, 101 MHz):** δ_C 193.0, 165.2, 162.8, 160.2, 142.2, 136.0, 135.9, 129.45, 129.44, 129.41, 115.2, 106.6, 68.8, 68.7, 50.6, 41.9, 32.4, 28.0. **HRMS (ESI) m/z:** $[M+Na]^+$ calcd. for $C_{26}H_{24}O_6SNa$: 487.1191; Found 487.1186.

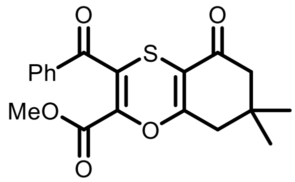

Starting from iodonium ylide **2a** (34.8 mg, 0.102 mmol) and pyridinium 1,4-zwitterionic thiolate **1g** (91.9 mg, 0.307 mmol), compound **3ga** was obtained as a yellow solid (8.7 mg, 25% yield).

MW ($C_{19}H_{18}O_5S$): 358.41 g/mol; **Rf:** 0.65 (Hexanes/EtOAc 7:3). **MP (ºC):** 137-140. **IR (ATR) ν (cm^{-1}):** 3048, 2920, 1714, 1672, 1632, 1587, 1241, 1121, 692. **^{1}H NMR (Acetone d_6, 400 MHz):** δ_H 8.08 – 8.01 (m, 2H), 7.73 – 7.66 (m, 1H), 7.60 – 7.53 (m, 2H), 3.54 (s, 3H), 2.51 (s, 2H), 2.41 (s, 2H), 1.15 (s, 6H). **^{13}C{H} NMR (Acetone d_6, 101 MHz):** δ_C 192.8, 188.9, 167.0, 159.6, 135.6, 135.4, 135.1, 129.9, 129.7, 127.8, 105.4, 52.8, 50.8, 42.4, 32.5, 28.1. **HRMS (ESI) m/z:** $[M+Na]^+$ calcd. for $C_{16}H_{20}O_6SNa$ 381.0767; Found 381.0759.

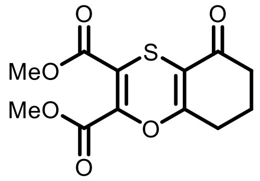

Starting from iodonium ylide **2b** (31.4 mg, 0.100 mmol) and pyridinium 1,4-zwitterionic thiolate **1a** (76 mg, 0.300 mmol), compound **3ab** was obtained as a yellow solid (13.0 mg, 46% yield).

MW ($C_{12}H_{12}O_6S$): 284.28 g/mol; **Rf:** 0.31 (Hexanes/EtOAc 6:4). **MP (ºC):** 96-97. **IR (ATR) ν (cm^{-1}):** 2923, 1717, 1657, 1597, 1431, 1179, 710. **^{1}H NMR (Acetone d_6, 400 MHz):** δ_H 3.80 (s, 3H), 3.78 (s, 3H), 2.51 (t, 2H, J = 6.2 Hz,), 2.46 (dd, 2H, J = 7.1, 6.2 Hz), 2.08-2.02 (m, 2H, overlapped with acetoned_6). **^{13}C{H} NMR (Acetone d_6, 101 MHz):** δ_C 193.2, 167.1, 163.5, 160.9, 142.0, 115.2, 107.6, 53.6, 53.5, 36.8, 28.6, 20.3. **HRMS (ESI) m/z:** $[M+Na]^+$ calcd. for $C_{12}H_{12}O_6SNa$: 307.0252; Found: 307.0247.

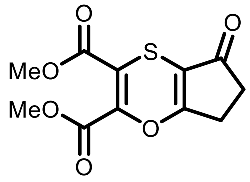

Starting from iodonium ylide **2c** (30.0 mg, 0.100 mmol) and pyridinium 1,4-zwitterionic thiolate **1a** (76.0 mg, 0.300 mmol), compound **3ac** was obtained as a yellow solid (18.8 mg, 70% yield).

MW ($C_{11}H_{10}O_6S$): 270.26 g/mol; **Rf:** 0.26 (Hexanes/EtOAc 6:4). **MP (ºC):** 65-66. **IR (ATR) ν (cm^{-1}):** 2954, 1716, 1651, 1596, 1425, 1332, 1235, 1195, 806, 730. **^{1}H NMR (Acetone d_6, 400 MHz):** δ_H 3.83 (s, 3H), 3.79 (s, 3H), 2.69-2.65 (m, 2H), 2.56-2.52 (m, 2H). **^{13}C{H} NMR (Acetone d_6, 101 MHz):** δ_C 197.7, 176.8, 163.2, 160.9, 143.7, 112.0, 108.9, 53.9, 53.7, 34.2, 27.2. **HRMS (ESI) m/z:** $[M+Na]^+$ calcd. for $C_{11}H_{10}O_6SNa$: 293.0096; Found: 293.0090.

A mmol scale reaction was carried using the following amounts of materials: iodonium ylide **2c** (300 mg, 1.000 mmol), pyridinium 1,4-zwitterionic thiolate **1a** (760 mg, 3.000 mmol) and CuI (38.0 mg, 0.200 mmol) in anhydrous dichloromethane (32 mL) affording compound **3ac** (198.0 mg, 73% yield).

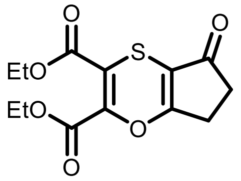

Starting from iodonium ylide **2c** (30.0 mg, 0.100 mmol) and pyridinium 1,4-zwitterionic thiolate **1b** (84.6 mg, 0.301 mmol), compound **3bc** was obtained as a yellow oil (24.0 mg, 81% yield).

MW ($C_{13}H_{14}O_6S$): 298.31 g/mol; **Rf:** 0.70 (Hexanes/EtOAc 6:4). **IR (ATR) ν (cm^{-1}):** 2980, 1712, 1667, 1556, 1367, 1228, 1015, 662. **^{1}H NMR (Acetone d$_6$, 400 MHz):** δ_H 4.28 (q, 2H, J = 8 Hz), 4.24 (q, 2H, J = 8 Hz), 2.70 – 2.65 (m, 2H), 2.56 – 2.52 (m, 2H), 1.30 (t, 3H, J = 8 Hz), 1.27 (t, 3H, J = 8 Hz). **^{13}C{H} NMR (Acetone d$_6$, 101 MHz):** δ_C 197.8, 176.8, 162.7, 160.5, 144.1, 111.6, 109.2, 63.5, 63.4, 34.2, 27.2, 14.08, 14.07. **HRMS (ESI) m/z:** [M+Na]$^+$ calcd. for $C_{13}H_{14}O_6SNa$: 321.0398; Found 321.0403.

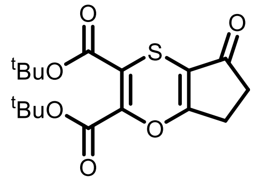

Starting from iodonium ylide **2c** (30.0 mg, 0.100 mmol) and pyridinium 1,4-zwitterionic thiolate **1d** (101.0 mg, 0.322 mmol), compound **3dc** was obtained as a yellow solid (23.8 mg, 67% yield).

MW ($C_{17}H_{22}O_6S$): 354.42 g/mol; **Rf:** 0.59 (Hexanes/EtOAc 6:4). **MP (ºC):** 120-121 (decomposition). **IR (ATR) ν (cm^{-1}):** 2981, 2925, 1731, 1699, 1654, 1600, 1356, 1052, 819.**^{1}H NMR (Acetone d$_6$, 400 MHz):** δ_H 2.67 – 2.61 (m, 2H), 2.56 – 2.50 (m, 2H), 1.51 (s, 9H), 1.49 (s, 9H). **^{13}C{H} NMR (Acetone d$_6$, 101 MHz):** δ_C 197.9, 176.8, 161.6, 159.7, 145.2, 110.9, 109.7, 84.8, 84.6, 34.2, 27.9, 27.2. **HRMS (ESI) m/z:** [M+Na]$^+$ calcd. for $C_{17}H_{22}NaO_6S$: 377.1029; Found: 377.1028.

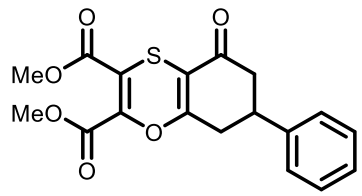

Starting from iodonium ylide **2d** (39.0 mg, 0.100 mmol) and pyridinium 1,4-zwitterionic thiolate **1a** (76.0 mg, 0.300 mmol), compound **3ad** was obtained as a yellow oil (21.0 mg, 58% yield).

MW ($C_{18}H_{16}O_6S$): 360.38 g/mol; **Rf:** 0.45 (Hexanes/EtOAc 6:4). **IR (ATR) ν (cm^{-1}):** 2952,1722, 1650, 1601, 1433, 1257, 760, 699. **^{1}H NMR (Acetone d$_6$, 400 MHz):** δ_H 7.44 – 7.33 (m, 4H), 7.35 – 7.23 (m, 1H), 3.80 (s, 6H), 3.60 – 3.48 (m, 1H), 2.91 – 2.81 (m, 2H), 2.74 – 2.64 (m, 2H). **^{13}C{H} NMR (Acetone d$_6$, 101 MHz):** δ_C 192.5, 166.1, 163.4, 160.9, 143.1, 142.2, 129.6 ,128.0, 127.8, 115.0, 107.7, 53.7, 53.5, 43.9, 38.5, 36.0. **HRMS (ESI) m/z:** [M+Na]$^+$ calcd. for $C_{18}H_{16}O_6SNa$: 383.0565; Found: 383.0556.

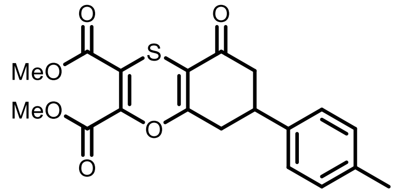

Starting from iodonium ylide **2e** (40.4 mg, 0.100 mmol) and pyridinium 1,4-zwitterionic thiolate **1a** (76.0 mg, 0.300 mmol), compound **3ae** was obtained as a yellow solid (20.0 mg, 54% yield).

MW ($C_{19}H_{18}O_6S$): 374,41 g/mol; **Rf:** 0.49 (Hexanes/EtOAc 6:4). **MP (ºC):** 122-123. **IR (ATR) *ν* (cm^{-1}):** 2919, 1721, 1651, 1604, 1123, 799. **^{1}H NMR (Acetone d$_6$, 400 MHz):** δ_H 7.32 – 7.24 (m, 2H), 7.22 – 7.14 (m, 2H), 3.800 (s, 3H), 3.799 (s, 3H), 3.58 – 3.42 (m, 1H), 2.88 – 2.75 (m, 2H), 2.73 – 2.61 (m, 2H), 2.31 (s, 3H). **^{13}C{H} NMR (Acetone d$_6$, 101 MHz):** δ_C 192.6, 166.1, 163.4, 160.9, 142.2, 140.1, 137.4, 130.2, 127.6, 115.0, 107.7, 53.7, 53.5, 44.0, 38.1, 36.1, 21. 0. **HRMS (ESI) m/z:** [M+Na]$^+$ calcd. for $C_{19}H_{18}O_6SNa$: 397.0722; Found: 397.0726.

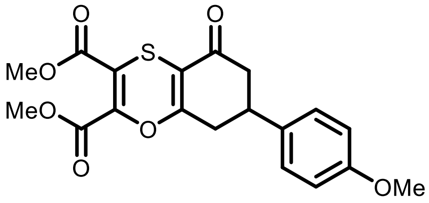

Starting from iodonium ylide **2f** (46.9 mg, 0.116 mmol) and pyridinium 1,4-zwitterionic thiolate **1a** (85.0 mg, 0.335 mmol), compound **3af** was obtained as a yellow oil (26.2 mg, 60% yield).

MW ($C_{19}H_{18}O_7S$): 390.41 g/mol; **Rf:** 0.42 (Hexanes/EtOAc 6:4). **IR (ATR) *ν* (cm^{-1}):** 2952, 1721, 1649, 1600, 1511, 1432, 1121, 828. **^{1}H NMR (Acetone d$_6$, 400 MHz):** δ_H 7.35 – 7.27 (m, 2H), 6.95 – 6.87 (m, 2H), 3.801 (s, 3H), 3.798 (s, 3H), 3.788 (s, 3H), 3.54 – 3.40 (m, 1H), 2.80 – 2.76 (m, 2H), 2.69 – 2.61 (m, 2H). **^{13}C{H} NMR (Acetone d$_6$, 101 MHz):** δ_C 192.6, 166.2, 163.4, 160.9, 159.8, 142.2, 135.0, 128.8, 115.0, 114.9, 107.7, 55.5, 53.7, 53.5, 44.1, 37.8, 36.3. **HRMS (ESI) m/z:** [M+Na]$^+$ calcd. for $C_{19}H_{18}O_7SNa$: 413.067; Found: 413.0666.

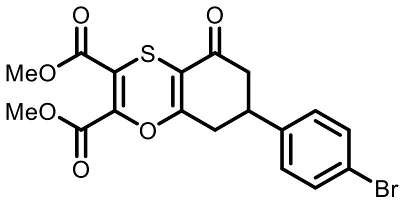

Starting from iodonium ylide **2g** (47.0 mg, 0.100 mmol) and pyridinium 1,4-zwitterionic thiolate **1a** (76.0 mg, 0.300 mmol), compound **3ag** was obtained as a yellow solid (26.0 mg, 59% yield).

MW ($C_{18}H_{15}BrO_6S$): 439.28 g/mol; **Rf:** 0.50 (Hexanes/EtOAc 6:4). **MP (ºC):** 125-126. **IR (ATR) *ν* (cm^{-1}):** 2948, 1729, 1642, 1596, 1429, 1127, 1050, 821. **^{1}H NMR (Acetone d$_6$, 400 MHz):** δ_H 7.59 – 7.49 (m, 2H), 7.42 – 7.35 (m, 2H), 3.80 (s, 6H), 3.65 – 3.49 (m, 1H), 2.90 – 2.78 (m, 2H), 2.74 – 2.60 (m, 2H). **^{13}C{H} NMR (Acetone d$_6$, 101 MHz):** δ_C 192.2, 165.9, 163.4, 160.8, 142.5, 142.2, 132.6, 130.0, 121.3, 115.0, 107.8, 53.7, 53.5, 43.6, 38.0, 35.7. **HRMS (ESI) m/z:** [M+Na]$^+$ calcd. for $C_{18}H_{15}BrO_6SNa$: 460.9665-462.9645; Found: 460.9671-462.9655.

A mmol scale reaction was carried using the following amounts of materials: iodonium ylide **2g** (470.1 mg, 1.002 mmol), pyridinium 1,4-zwitterionic thiolate **1a** (759.6 mg, 2.999 mmol) and CuI (38.0 mg, 0.200 mmol) in anhydrous dichloromethane (32 mL) affording compound **3ag** (267.0 mg, 61% yield)

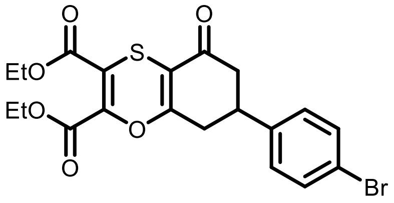

Starting from iodonium ylide **2g** (45.5 mg, 0.097 mmol) and pyridinium 1,4-zwitterionic thiolate **1b** (84.6 mg, 0.301 mmol), compound **3bg** was obtained as a yellow oil (23.0 mg, 51% yield).

MW ($C_{20}H_{19}BrO_6S$): 467.33 g/mol; **Rf:** 0.25 (Hexanes/EtOAc 9:1). **IR (ATR) ν (cm^{-1}):** 2978, 1719, 1648, 1605, 1366, 1250, 1123, 820. **^{1}H NMR (Acetone d$_6$, 400 MHz):** δ_H 7.59 – 7.51 (m, 2H), 7.42 – 7.34 (m, 2H), 4.30 – 4.20 (m, 4H), 3.64 – 3.50 (m, 1H), 2.90 – 2.82 (m, 1H), 2.81 – 2.76 (m, 1H), 2.75 – 2.67 (m, 1H), 2.71 – 2.63 (m, 1H), 1.28 (t, 6H, J = 7.2 Hz). **^{13}C{H} NMR (Acetone d$_6$, 101 MHz):** δ_C 192.2, 166.0, 162.9, 160.4, 142.54, 142.50, 132.6, 130.0, 121.3, 114.8, 107.9, 63.3, 63.1, 43.6, 38.0, 35.7, 14.12, 14.10. **HRMS (ESI) m/z:** $[M+Na]^+$ calcd. for $C_{20}H_{19}BrO_6SNa$: 488.9978-490.9958; Found 488.9972-490.9954.

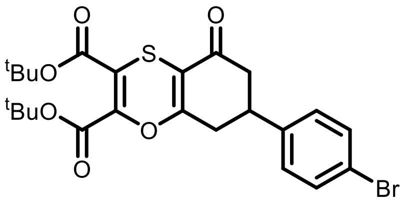

Starting from iodonium ylide **2g** (46.9 mg, 0.100 mmol) and pyridinium 1,4-zwitterionic thiolate **1d** (101.0 mg, 0.300 mmol), compound **3dg** was obtained as a yellow solid (23.0 mg, 44% yield).

MW ($C_{24}H_{27}BrO_6S$): 523.44 g/mol; **Rf:** 0.64 (Hexanes/EtOAc 6:4). **MP (ºC):** 128-130 (decomposition). **IR (ATR) ν (cm^{-1}):** 2973, 1709, 1596, 1367, 1296, 1022, 816. **^{1}H NMR (Acetone d$_6$, 400 MHz):** δ_H 7.60 – 7.49 (m, 2H), 7.41 – 7.34 (m, 2H), 3.63 – 3.47 (m, 1H), 2.92 – 2.76 (m, 2H), 2.73 – 2.61 (m, 1H), 1.50 (s, 9H), 1.49 (s, 9H). **^{13}C{H} NMR (Acetone d$_6$, 101 MHz):** δ_C 192.3, 166.1, 161.8, 159.7, 143.5, 142.6, 132.6, 130.0, 121.3, 114.5, 108.4, 84.4, 84.3, 43.6, 38.1, 35.8, 28.0, 27. 9. **HRMS (ESI) m/z:** $[M+Na]^+$ calcd. for $C_{24}H_{27}BrNaO_6S$: 545.0604-547.0584; Found: 545.0614-547.0592.

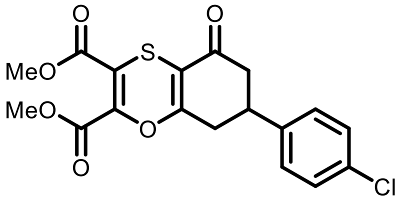

Starting from iodonium ylide **2h** (42.5 mg, 0.100 mmol) and pyridinium 1,4-zwitterionic thiolate **1a** (76.0 mg, 0.300 mmol), compound **3ah** was obtained as a yellow solid (23.4 mg, 59% yield).

MW ($C_{18}H_{15}ClO_6S$): 394.82 g/mol; **Rf:** 0.49(Hexanes/EtOAc 6:4). **MP (ºC):** 117-118. **IR (ATR) ν (cm^{-1}):** 2951, 1730, 1643, 1428, 1258, 980, 824. **^{1}H NMR (Acetone d$_6$, 400 MHz):** δ_H 7.49 – 7.36 (m, 4H), 3.80 (s, 6H), 3.64 – 3.50 (m, 1H), 2.90 – 2.77 (m, 2H), 2.76 – 2.64 (m, 2H). **^{13}C{H} NMR (Acetone d$_6$, 101 MHz):** δ_C 192.2, 166.0, 163.4, 160.8, 142.2, 142.0, 133.2, 129.64, 129.61, 115.0, 107.8, 53.7, 53.5, 43.7, 37.9, 35.8. **HRMS (ESI) m/z:** $[M+Na]^+$ calcd. for $C_{18}H_{15}ClO_6SNa$: 417.0176; Found: 417.0172.

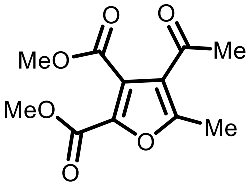

Starting from iodonium ylide **2i** (82.5 mg, 0.272 mmol) and pyridinium 1,4-zwitterionic thiolate **1a** (207.2 mg, 0.818 mmol), compound **3'ai** was obtained as a yellow oil (33.0 mg, 50% yield) alongside compound **6ai** (18 mg, 24% yield) as a colorless solid.

Spectral data for **3'ai**

MW ($C_{11}H_{12}O_6S$): 240.21 g/mol; **Rf:** 0.70 (Hexanes/EtOAc 6:4). **^{1}H NMR (Acetone d$_6$, 400 MHz):** δ_H 3.87 (s, 3H), 3.82 (s, 3H), 2.66 (s, 3H), 2.42 (s, 3H). **^{13}C{H} NMR (Acetone d$_6$, 101 MHz):** δ_C 186.4, 164.1, 162.7, 162.5, 147.7, 124.8, 115.0, 53.0, 52.3, 26.4, 14.2. **HRMS (ESI) m/z:** [M+Na]$^+$ calcd. for $C_{11}H_{12}O_6SNa$: 263.0526; Found: 263.0534.

The spectroscopic data agrees with those previously reported in the literature.[8]

Spectral data for **6ai**

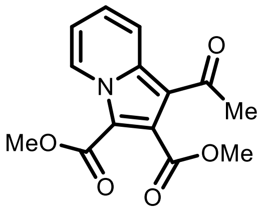

MW ($C_{14}H_{13}O_5N$): 275.26 g/mol; **Rf:** 0.45 (Hexanes/EtOAc 6:4). **^{1}H NMR ($CDCl_3$, 400 MHz):** δ_H 9.53 (dt, 1H, J = 7.1, 1.1 Hz), 8.54 (dt, 1H, J = 9.0, 1.2 Hz), 7.44 (ddd, 1H, J = 9.0, 6.8, 1.2 Hz), 7.09 (td, 1H, 7.0, 1.4 Hz), 4.02 (s, 3H), 3.92 (s, 3H), 2.5 (s, 3H). **^{13}C{H} NMR ($CDCl_3$, 101 MHz):** δ_C 192.0, 167.3, 160.7, 138.0, 130.0, 128.0, 120.8, 116.2, 112.3, 112.2, 53.3, 52.2, 29.2.

The spectroscopic data agrees with those previously reported in the literature by us.[9]

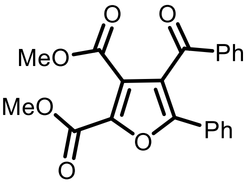

Starting from iodonium ylide **2j** (63.1 mg, 0.148 mmol) and pyridinium 1,4-zwitterionic thiolate **1a** (112.5 mg, 0.444 mmol), compound **3'aj** was obtained as a yellow solid (27.9 mg, 52% yield).

MW ($C_{21}H_{16}O_6$): 364.35 g/mol; **Rf:** 0.45 (Hexanes/EtOAc 7:3). **^{1}H NMR (Acetone d$_6$, 400 MHz):** δ_H 8.16 – 8.08 (m, 2H), 8.07 – 7.98 (m, 2H), 7.76 – 7.68 (m, 1H), 7.66 – 7.59 (m, 2H), 7.61 – 7.52 (m, 3H), 3.84 (s, 3H), 3.83 (s, 3H). **^{13}C{H} NMR (Acetone d$_6$, 101 MHz):** δ_C 182.2, 163.7, 162.7, 159.1, 148.8, 137.1, 134.3, 131.8, 130.3, 129.7, 129.6, 129.4, 129.1, 128.4, 115.3, 53.0, 52.7. **HRMS (ESI) m/z:** [M+Na]$^+$ calcd. for $C_{21}H_{16}O_6Na$: 387.0839; Found: 387.0835.

The spectroscopic data agrees with those previously reported in the literature.[8]

S3. Further Functionalization

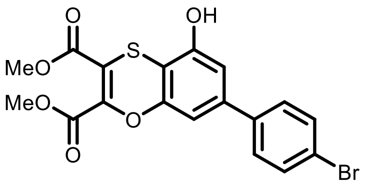

Prepared from an adapted procedure.[10] A mixture of compound **3ag** (43.9 mg, 0.100 mmol), K_2CO_3 (14.0 mg, 0.100 mmol, 1eq) and I_2 (25.0 mg, 0.100 mmol, 1eq) in DMF (1 mL) was heated to 90 °C (heating block) for 18 h. The reaction mixture was then cooled and extracted with EtOAc/H_2O, the organic phases dried, filtered and concentrated and the resulting residue was purified by column chromatography on silica gel using hexanes/EtOAc as the eluent (100:0 to 80:20). Product **4ag** was obtained as a yellow solid (28.0 mg, 65% yield).

An alternative method for the same transformation was also conducted: A mixture of compound **3ag** (43.9 mg, 0.100 mmol) and NIS (23.0 mg, 0.110 mmol, 1.1eq) was dissolved in DMSO (1 mL) and heated to 80 °C (heating block) for 24 h. The reaction was then cooled and extracted with EtOAc/H_2O, the organic phases dried, filtered and concentrated and the resulting residue was purified by column chromatography on silica gel using hexanes/EtOAc as the eluent (100:0 to 80:20). Product **4ag** was obtained as a yellow solid (24.5 mg, 56% yield).

MW ($C_{18}H_{13}BrO_6S$): 437.26 g/mol; **Rf:** 0.39 (Hexanes/EtOAc 6:4). **MP (ºC):** 223-224. **IR (ATR) ν (cm^{-1}):** 3328, 2942, 1739, 1702, 1581, 1425, 1202, 1157, 822. **^{1}H NMR (Acetone d_6, 400 MHz):** δ_H 9.64 (bs, 1H), 7.65 – 7.58 (m, 2H), 7.58 – 7.51 (m, 2H), 6.95 (d, 1H, J = 1.7 Hz), 6.73 (d, 1H, J = 1.7 Hz), 3.85 (s, 3H), 3.81 (s, 3H). **^{13}C{H} NMR (Acetone d_6, 101 MHz):** δ_C 163.5, 161.8, 154.4, 151.2, 146.4, 141.4, 139.1, 132.8, 129.4, 122.6, 111.5, 110.2, 108.0, 104.5, 53.5, 53.4. **HRMS (ESI) m/z:** $[M+Na]^+$ calcd. for $C_{18}H_{13}BrO_6SNa$: 458.9508-460.9489; Found: 458.9505-460.9487.

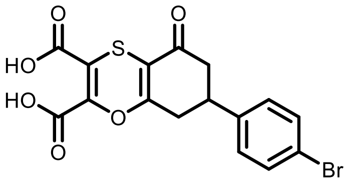

To a stirred solution of compound **3ag** (43.9 mg, 0.100 mmol) in THF (1mL), NaOH (1M, 1mL) was added dropwise. Upon addition the reaction crude turned pink and after 1 minute the reaction was quenched with diluted HCl and acidified until pH = 2. Then, the mixture was extracted with dichloromethane and water. The combined organic extracts were dried over anhydrous Na_2SO_4 and concentrated under reduced pressure. Finally, pentane washings of the resulting residue afforded compound **5ag** as a yellow solid which decomposed over time (25.0 mg, 61% yield).

MW ($C_{16}H_{11}BrO_6S$): 409.95 g/mol; **Rf:** 0.24 (Hexanes/EtOAc 3:7). **MP (ºC):** 130-138 (decomposition). **IR (ATR) ν (cm^{-1}):** 3019, 2952, 1730, 1558, 1487, 1201, 1141, 818. **^{1}H NMR (Acetone d_6, 400 MHz):** δ_H 7.59 – 7.50 (m, 2H), 7.45 – 7.30 (m, 2H), 3.95 – 3.60 (m, 2H), 3.00 – 2.57 (m, 3H). **^{13}C{H} NMR (Acetone d_6, 101 MHz):** δ_C 193.4, 172.9, 160.1, 143.3, 143.2, 132.5, 130.1, 130.0, 121.1, 121.0, 107.6, 43.9, 38.3, 37.2. **HRMS (ESI) m/z:** $[M-H]^+$ calcd. for $C_{16}H_{11}BrO_6S$: 408.9387-410.9367; Found: 408.9480-410.9418.

S4. Crystal structure of compound 3ah with probability level of 50 %

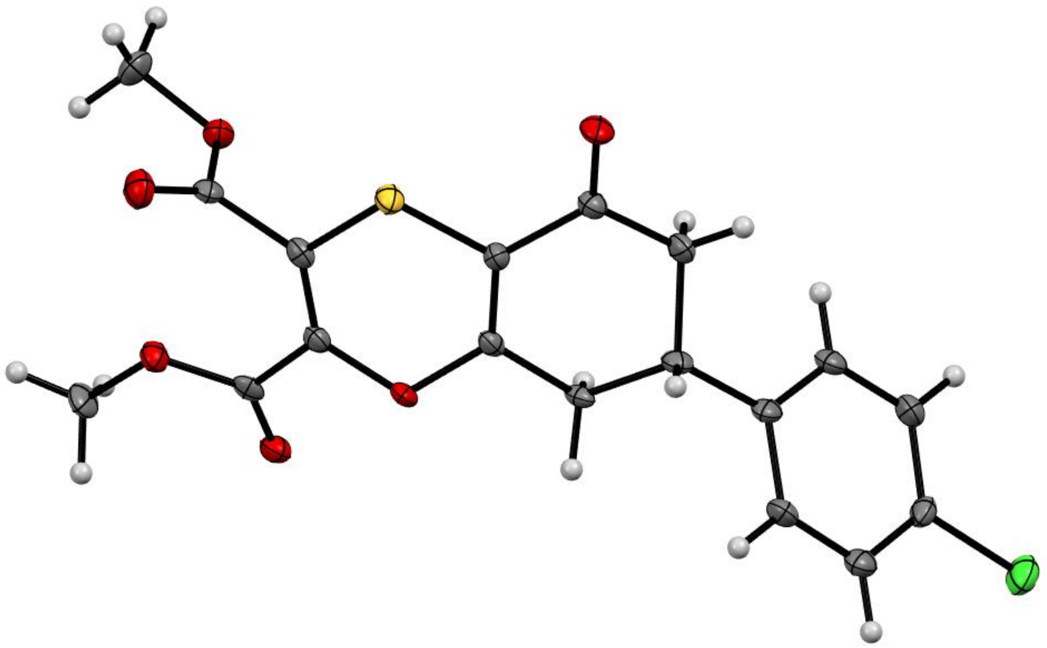

Crystals of **3ah** were obtained via slow evaporation at 0-4 °C of a supersaturated solution of **3ah** in a mixture of DCM/hexanes.

A yellow, prism-like specimen of $C_{18}H_{15}ClO_6S$, approximate dimensions 0.170 mm x 0.190 mm x 0.320 mm, was used for the X-ray crystallographic analysis. The X-ray intensity data were measured on a D8 QUEST ECO three-circle diffractometer system equipped with a Ceramic x-ray tube (Mo Kα, λ = 0.71073 Å) and a doubly curved silicon crystal Bruker Triumph monochromator. A total of 2904 frames were collected. The total exposure time was 40.33 hours. The frames were integrated with the Bruker SAINT software package using a narrow-frame algorithm. The integration of the data using a monoclinic unit cell yielded a total of 18825 reflections to a maximum θ angle of 28.38° (0.75 Å resolution), of which 4094 were independent (average redundancy 4.598, completeness = 95.4%, R_{int} = 4.64%, R_{sig} = 4.51%) and 3477 (84.93%) were greater than $2\sigma(F^2)$. The final cell constants of a = 4.6520(6) Å, b = 11.6041(16) Å, c = 31.723(4) Å, β = 91.267(4)°, volume = 1712.1(4) $Å^3$, are based upon the refinement of the XYZ-centroids of 7279 reflections above 20 σ(I) with 5.138° < 2θ < 56.48°. Data were corrected for absorption effects using the Multi-Scan method (SADABS). The ratio of minimum to maximum apparent transmission was 0.857. The calculated minimum and maximum transmission coefficients (based on crystal size) are 0.8880 and 0.9380.

The structure was solved and refined using the Bruker SHELXTL Software Package, using the space group P 1 21/c 1, with Z = 4 for the formula unit, $C_{18}H_{15}ClO_6S$. The final anisotropic full-matrix least-squares refinement on F^2 with 245 variables converged at R1 = 9.84%, for the observed data and wR2 = 23.51% for all data. The goodness-of-fit was 1.260. The largest peak

in the final difference electron density synthesis was 0.682 $e^-/Å^3$ and the largest hole was -0.704 $e^-/Å^3$ with an RMS deviation of 0.151 $e^-/Å^3$. On the basis of the final model, the calculated density was 1.532 g/cm^3 and F(000), 816 e^-.

Sample and crystal data for SKt108_2

**Identification code**	SKt108_2	
**Chemical formula**	$C_{18}H_{15}ClO_6S$	
**Formula weight**	394.81 g/mol	
**Temperature**	100(2) K	
**Wavelength**	0.71073 Å	
**Crystal size**	0.170 x 0.190 x 0.320 mm	
**Crystal habit**	yellow prism	
**Crystal system**	monoclinic	
**Space group**	P 1 21/c 1	
**Unit cell dimensions**	a = 4.6520(6) Å	α = 90°
	b 11.6041(16)	β = 91.267(4)°
	c = 31.723(4)	γ = 90°
**Volume**	1712.1(4) $Å^3$	
**Z**	4	
**Density (calculated)**	1.532 g/cm^3	
**Absorption coefficient**	0.379 mm-1	
**F(000)**	816	

Data collection and structure refinement for SKt108_2

**Diffractometer**	D8 QUEST ECO three-circle diffractometer
**Radiation source**	Ceramic x-ray tube (Mo Kα, λ = 0.71073 Å)
**Theta range for data collection**	2.61 to 28.38°
**Index ranges**	-6<=h<=5, -15<=k<=15, -41<=l<=42
**Reflections collected**	18825
**Independent reflections**	4094 [R(int) = 0.0464]
**Coverage of independent reflections**	95.4%
**Absorption correction**	Multi-Scan

**Max. and min. transmission**	0.9380 and 0.8880
**Structure solution technique**	direct methods
**Structure solution program**	XT, VERSION 2018/2
**Refinement method**	Full-matrix least-squares on F2
**Refinement program**	SHELXL-2019/1 (Sheldrick, 2019)
**Function minimized**	Σ w(Fo2 - Fc2)2
**Data / restraints / parameters**	4094 / 0 / 245
**Goodness-of-fit on F2**	1.260
**Final R indices3477 data**;	I>2σ(I) R1 = 0.0984, wR2 = 0.2288
	all data R1 = 0.1114, wR2 = 0.2351
**Weighting scheme**	w=1/[σ2(Fo2)+(0.0186P)2+18.2863P]
	where P=(Fo2+2Fc2)/3
**Largest diff. peak and hole**	0.682 and -0.704 eÅ-3
**R.M.S. deviation from mean**	0.151 eÅ-3

Atomic coordinates and equivalent isotropic atomic displacement parameters ($Å^2$) for SKt108_2.

U(eq) is defined as one third of the trace of the orthogonalized Uij tensor.

	x/a	y/b	z/c	U(eq)
Cl25	0.0501(4)	0.42665(15)	0.94374(5)	0.0289(4)
S1	0.9963(3)	0.25071(13)	0.65066(4)	0.0176(3)
O4	0.6272(9)	0.4550(3)	0.67814(12)	0.0167(8)
O12	0.9094(10)	0.3271(4)	0.54805(13)	0.0240(9)
O13	0.5134(9)	0.2339(4)	0.56865(12)	0.0204(9)
O16	0.2213(9)	0.5554(3)	0.62958(12)	0.0189(8)
O17	0.4477(9)	0.4997(3)	0.57138(12)	0.0182(8)
O26	0.0200(9)	0.1015(4)	0.72501(13)	0.0219(9)
C2	0.7657(12)	0.3382(5)	0.61958(17)	0.0154(11)

C3	0.6164(12)	0.4250(5)	0.63591(17)	0.0161(11)
C5	0.6734(12)	0.3686(5)	0.70695(16)	0.0143(10)
C6	0.5340(12)	0.3934(4)	0.74783(17)	0.0135(10)
C7	0.6737(13)	0.3230(5)	0.78391(17)	0.0172(11)
C8	0.6977(13)	0.1972(5)	0.77097(17)	0.0187(11)
C9	0.8618(12)	0.1832(5)	0.73136(17)	0.0170(11)
C10	0.8238(12)	0.2733(5)	0.69923(16)	0.0155(11)
C11	0.7389(12)	0.3028(5)	0.57429(17)	0.0162(11)
C14	0.4661(15)	0.1906(6)	0.52611(18)	0.0268(14)
C15	0.4042(11)	0.5003(5)	0.61261(17)	0.0143(10)
C18	0.2506(14)	0.5706(5)	0.54647(19)	0.0249(13)
C19	0.5144(13)	0.3425(5)	0.82450(17)	0.0178(11)
C20	0.5539(14)	0.4494(5)	0.84449(18)	0.0215(12)
C21	0.4131(13)	0.4750(5)	0.88088(18)	0.0209(12)
C22	0.2297(13)	0.3945(5)	0.89750(17)	0.0194(12)
C23	0.1848(14)	0.2879(5)	0.87851(18)	0.0223(12)
C24	0.3281(13)	0.2642(5)	0.84195(18)	0.0191(11)

S5. References

(1) L. Moafi, S. Ahadi, H.R. Khavasi and A. Bazgir. Three-component diastereoselective synthesis of stable 1,4-diionic organosulfurs. *Synthesis* **2011**, *9*, 1399-1402.

(2) S. Duan, C. Chen, Y. Chen, Y. Jie, H. Luo, Z.-F. Xu, B. Cheng and C.-Y. Li. Two reaction modes of 1-sulfonyl-1,2,3-triazoles and pyridinium 1,4-zwitterionic thiolates: catalyst-free synthesis of pyrido[1,2-a]pyrazine derivatives and 1,4-thiazine derivatives. *Org. Chem. Front.,* **2021**,*8,* 6962-6967.

(3) Y. Yao, B. Lin, M. Wu, Y. Zhang, Y. Huang, X. Han and Z. Weng. Synthesis of 2-trifluoromethyl thiazoles via [3 + 2] cycloaddition of pyridinium 1,4-zwitterionic thiolates with CF_3CN. *Org. Biomol. Chem*., **2022**, *20*, 8761-8765.

(4) B. Cheng, X. Zhang, Y. Li, H. Li, Y. He, Y. Li, T. Wang and H. Zhai. Synthesis of indolizines from pyridinium 1,4-zwitterionic thiolates and α-functionalized bromoalkanes via a stepwise [(5+1)−1] pathway. *Chem. Commun.* **2020**, *56*, 8396-8399.

(5) S. Mayakrishnan, M.Tamizmani, N. Uma-Maheswari. Harnessing hypervalent iodonium ylides as carbene precursors: C–H activation of N-methoxybenzamides with a Rh(III)-catalyst. *Chem. Commun.,* **2020**, *56*, 15462-15465.

(6) C. A. Montgomery, I. Jameel, F. Cuzzucoli, T. Chidley, W. S. Hopkins and G. K. Murphy. σ-Holes in iodonium ylides: halogen-bond activation of carboxylic acids, phenols and thiophenols may enable their X-H insertion reactions. *Chem. Eur. J.,* **2022**, *28*, e20220202.

(7) J. Wang, M. Wang, K. Chen, S. Zha, C. Song and J. Zhu. C–H Activation-based traceless synthesis via electrophilic removal of a directing group. Rhodium(III)-catalyzed entry into indoles from N-nitroso and α-diazo-β-keto compounds. *Org. Lett*. **2016**, *18*, 1178–1181.

(8) R. Yan, J. Huang, J. Luo, P. Wen, H. Huang and Y. Liang. Copper(I)-catalyzed synthesis of polysubstituted furans from alkynoates and 1,3-dicarbonyl compounds in the presence of oxygen. *Synlett* **2010**, *7*, 1071-1074.

(9) R. Monreal-Corona, À. Díaz-Jiménez, A. Roglans, A. Poater, A. Pla-Quintana. Indolizine Synthesis through annulation of pyridinium 1,4-thiolates and copper carbenes: a predictive catalysis approach. *Adv. Synth. Catal.* **2023**, *365*, 760-766.

(10) W. Wu, X. Wu, S. Fan and J. Zhu. Rh(III)-Catalyzed enaminone-directed C–H coupling with diazodicarbonyls for skeleton-divergent synthesis of isocoumarins and naphthalenes. *Org. Lett*. 2022, *24*, 7850–7855.

S6. NMR SPECTRA

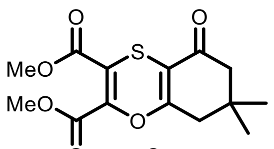

3aa

^{1}H NMR (Acetone d_6, 400 MHz)

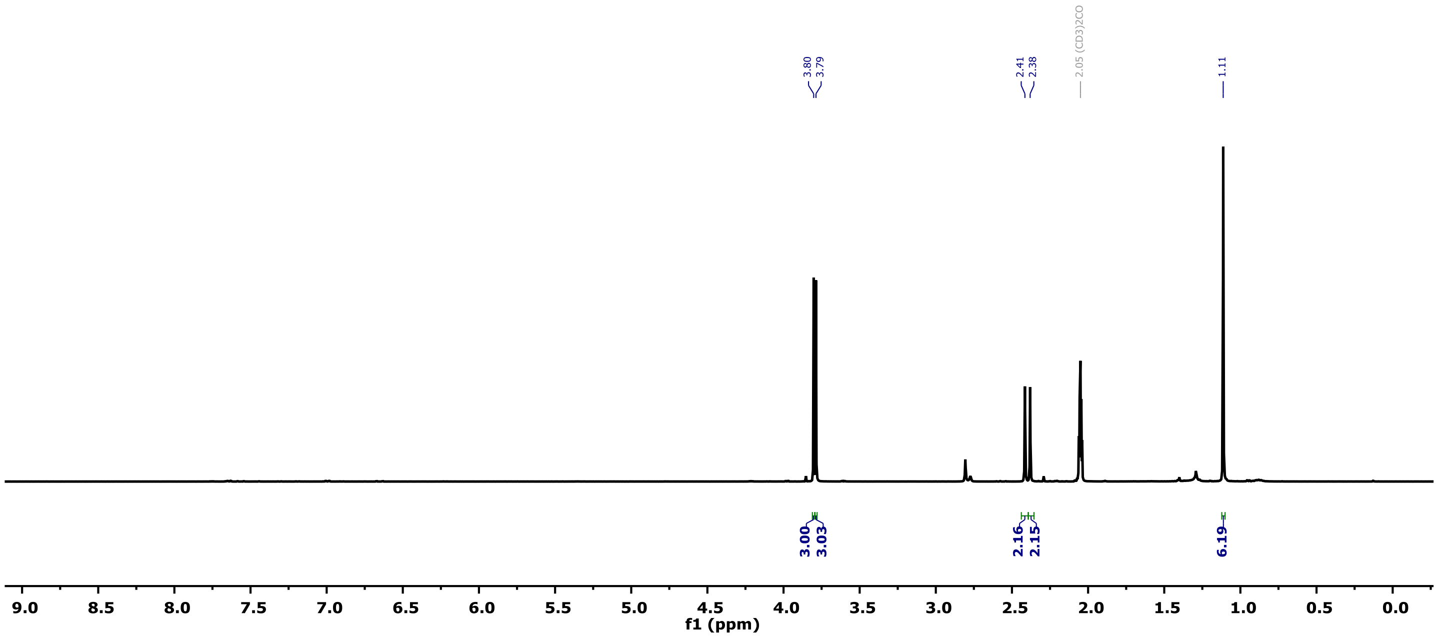

$^{13}C\{H\}$ NMR (Acetone d_6, 101 MHz)

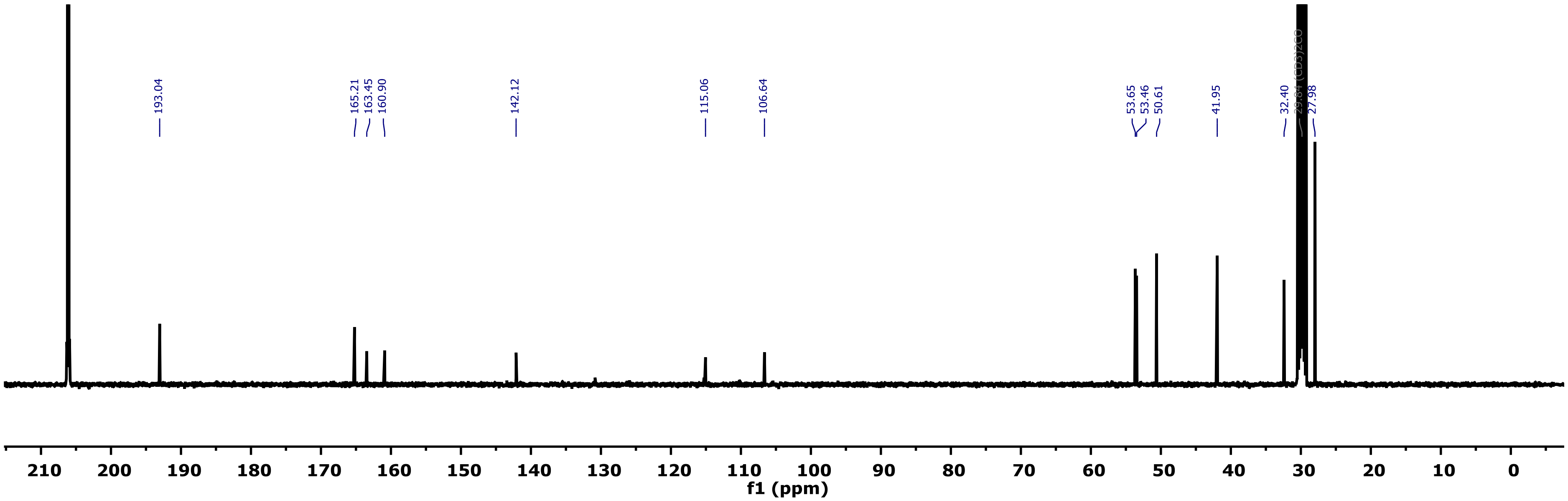

2D NMR HSQC (Acetone d_6)

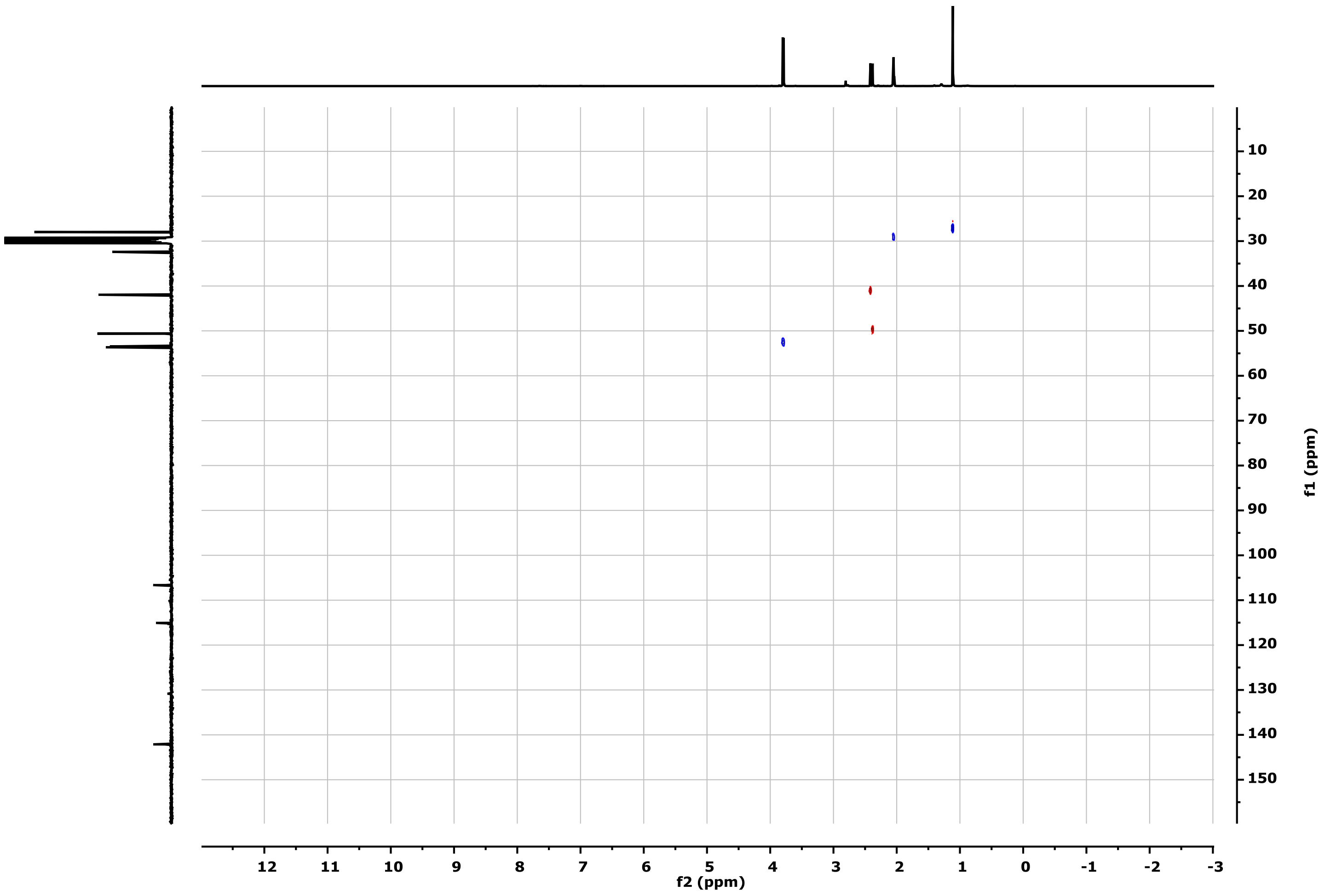

2D NMR COSY (Acetone d_6)

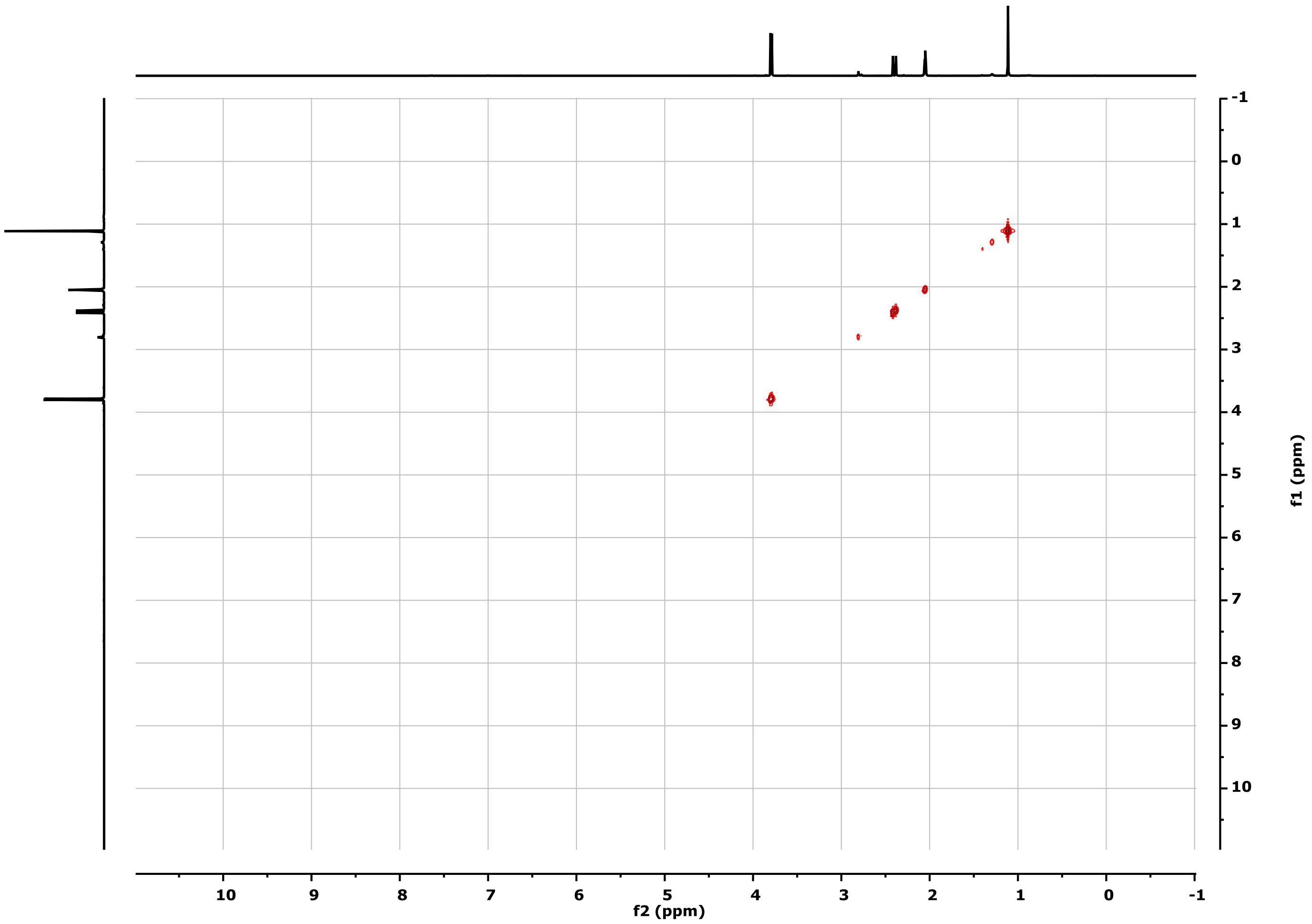

2D NMR HMBC (Acetone d_6)

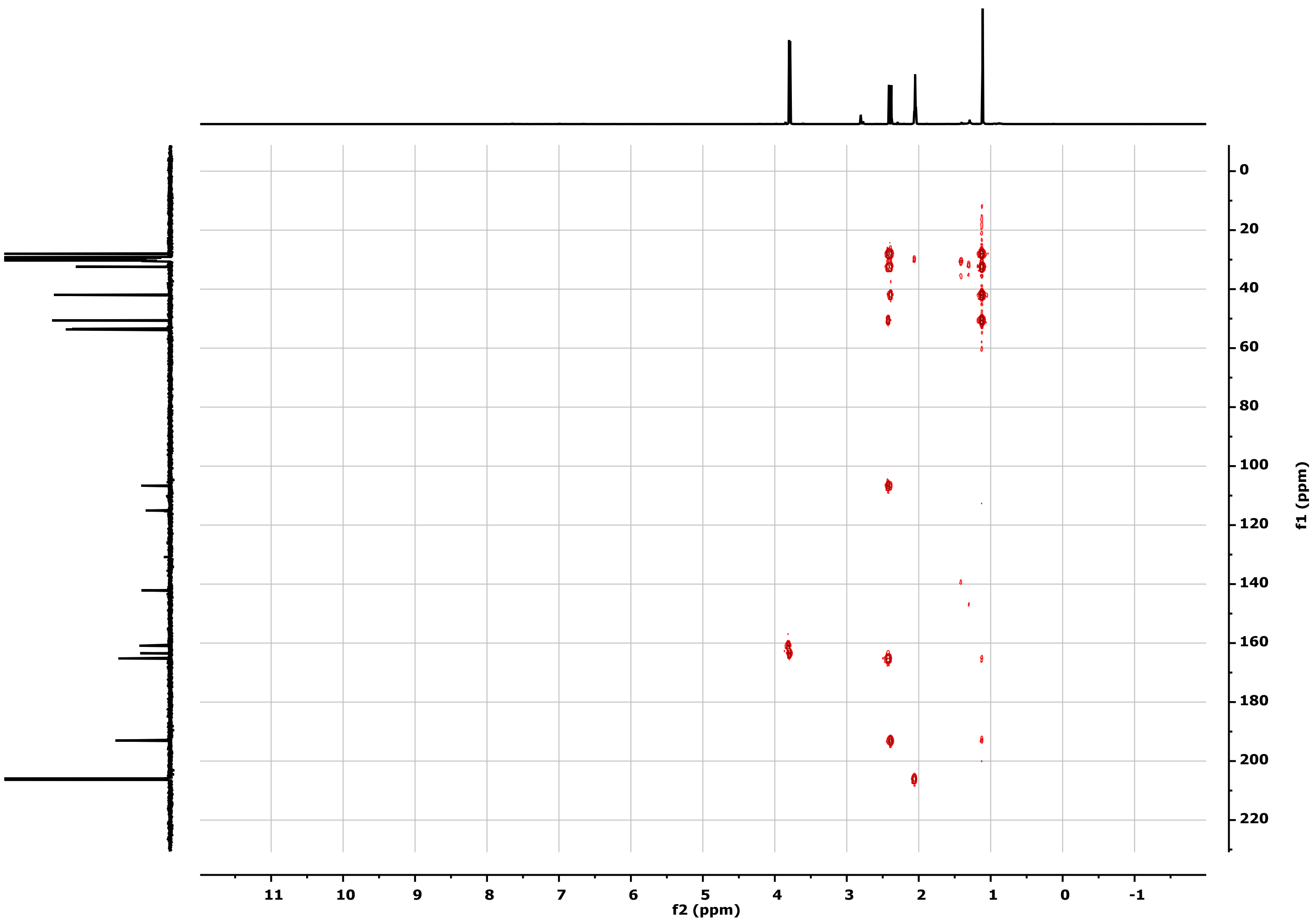

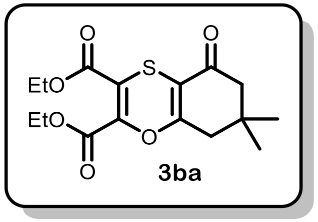

3ba

^{1}H NMR (Acetone d$_6$, 400 MHz)

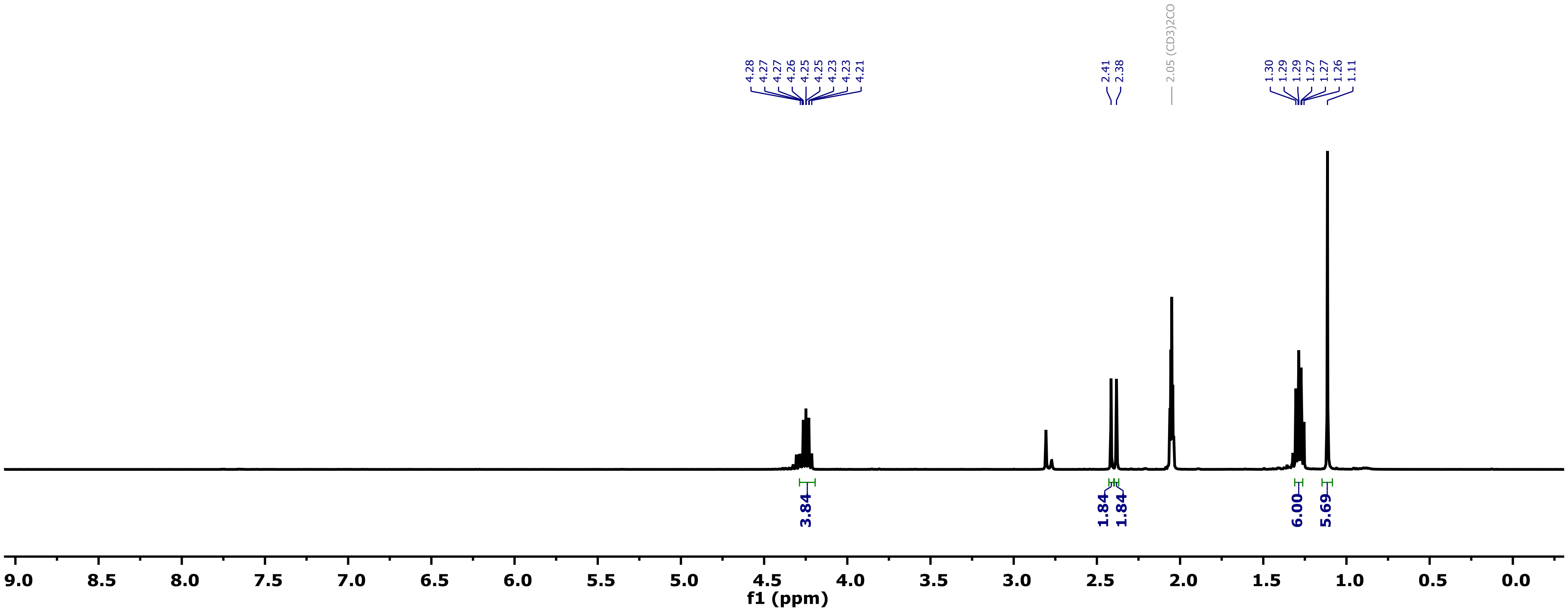

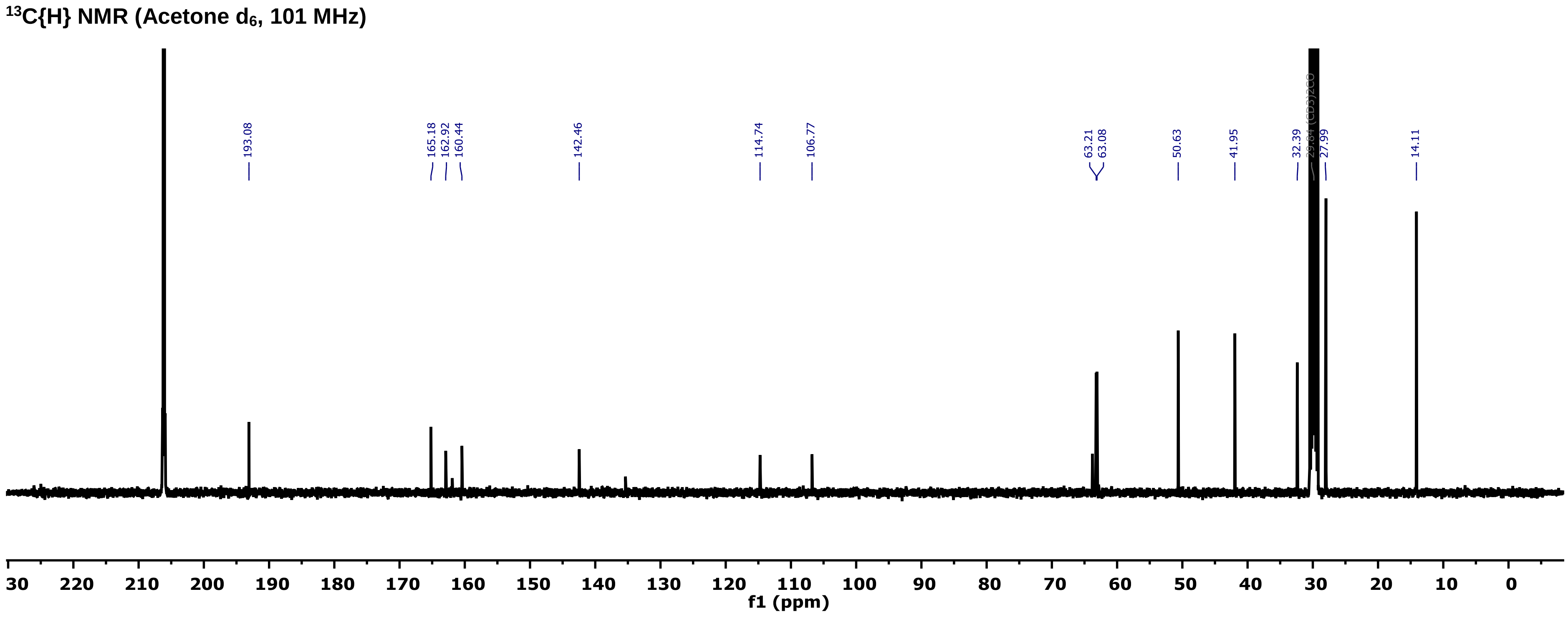

$^{13}C\{H\}$ NMR (Acetone d_6, 101 MHz)
193.08
165.18
162.92
160.44
142.46
114.74
106.77
63.21
63.08
50.63
41.95
32.39
29.84 (CD3)2CO
27.99
14.11
30
220
210
200
190
180
170
160
150
140
130
120
110
100
90
80
70
60
50
40
30
20
10
0
f1 (ppm)

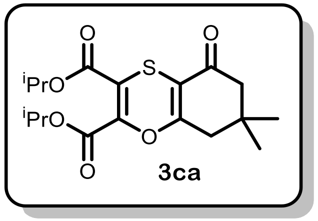

1H NMR (Acetone d_6, 400 MHz)

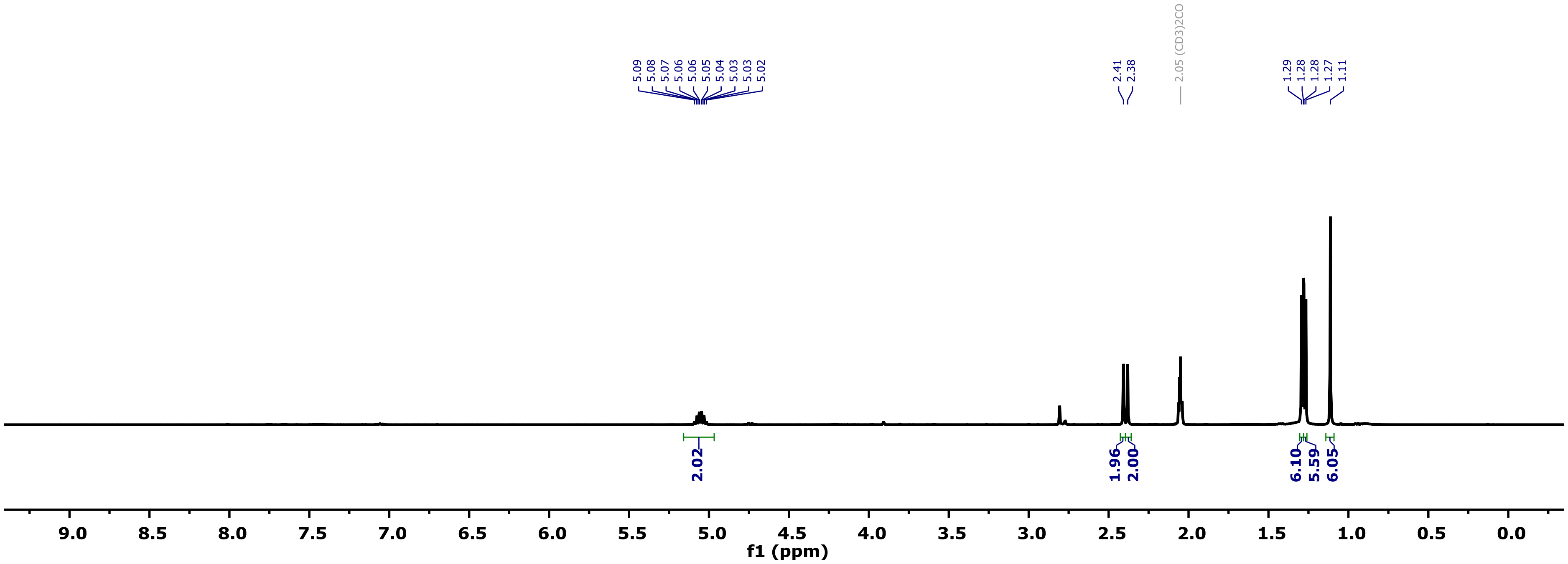

$^{13}C\{H\}$ NMR (Acetone d_6, 101 MHz)

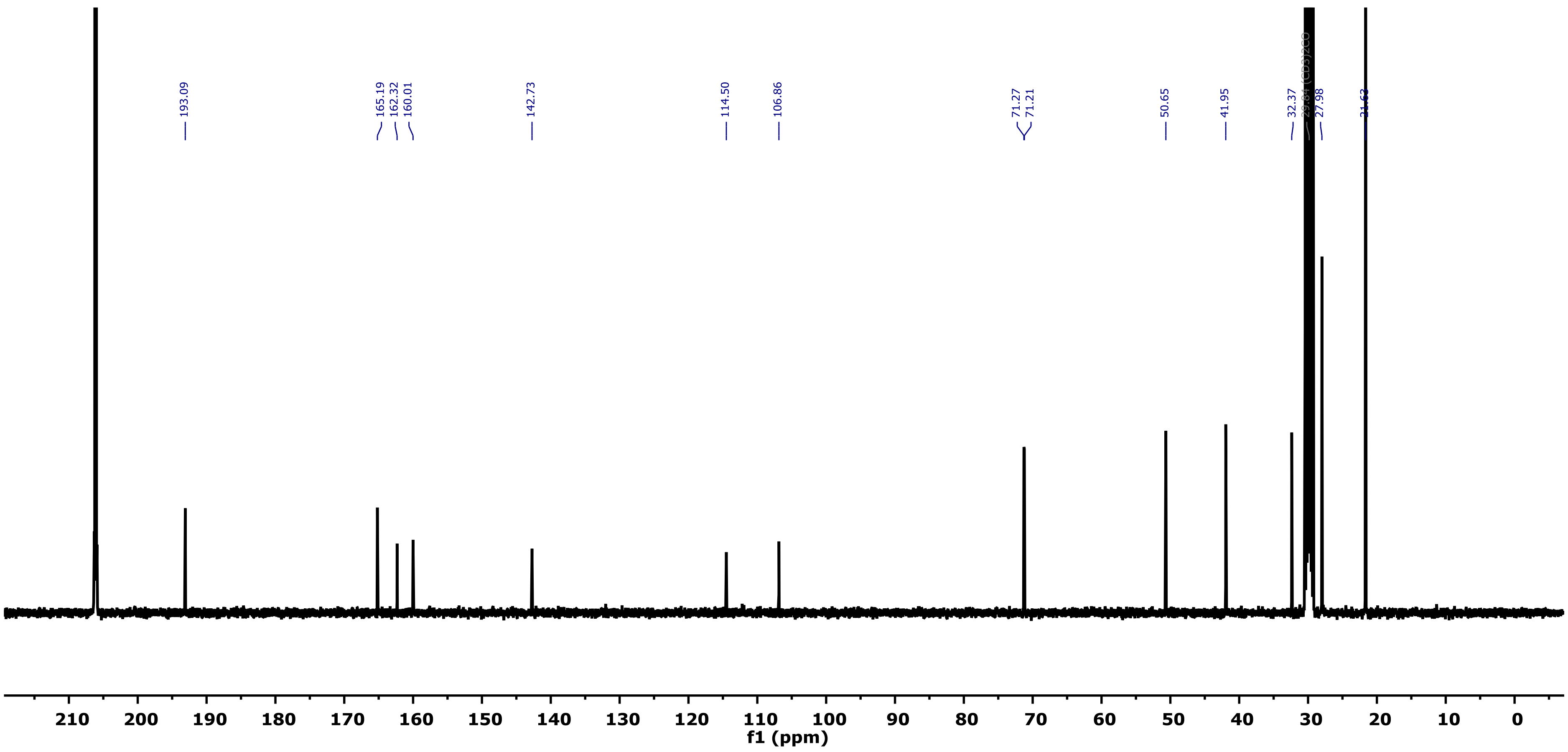

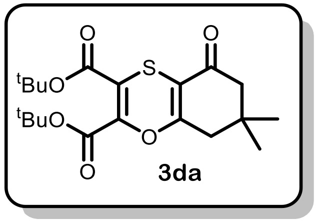

^1H NMR (Acetone d_6, 400 MHz)

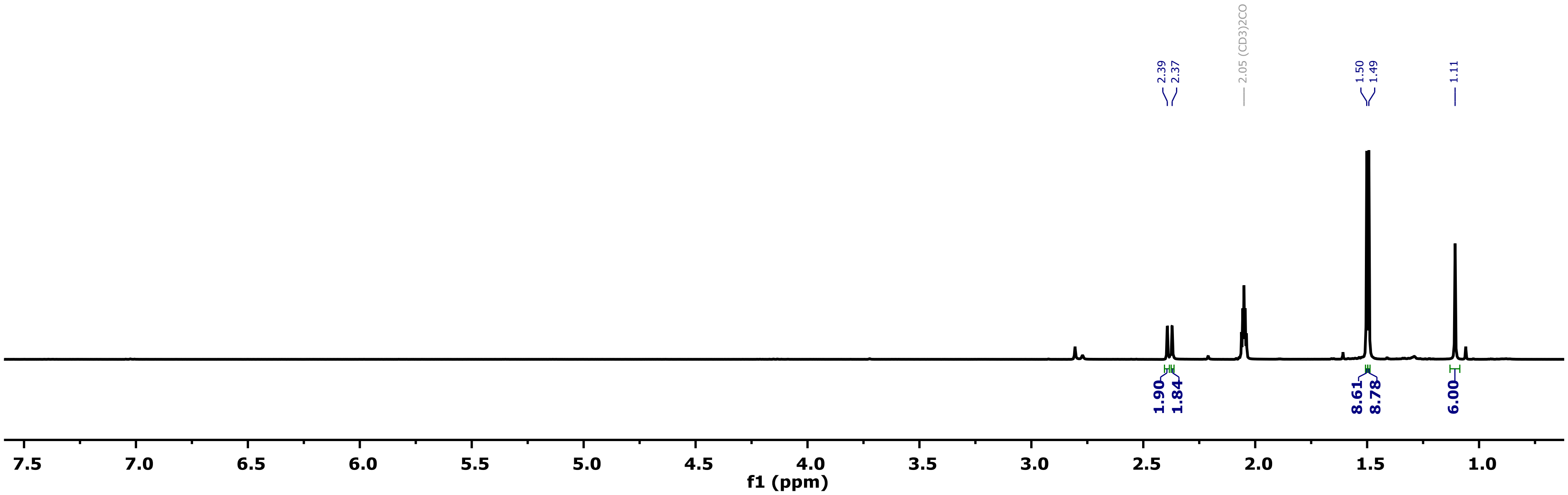

$^{13}C\{H\}$ NMR (Acetone d_6, 101 MHz)

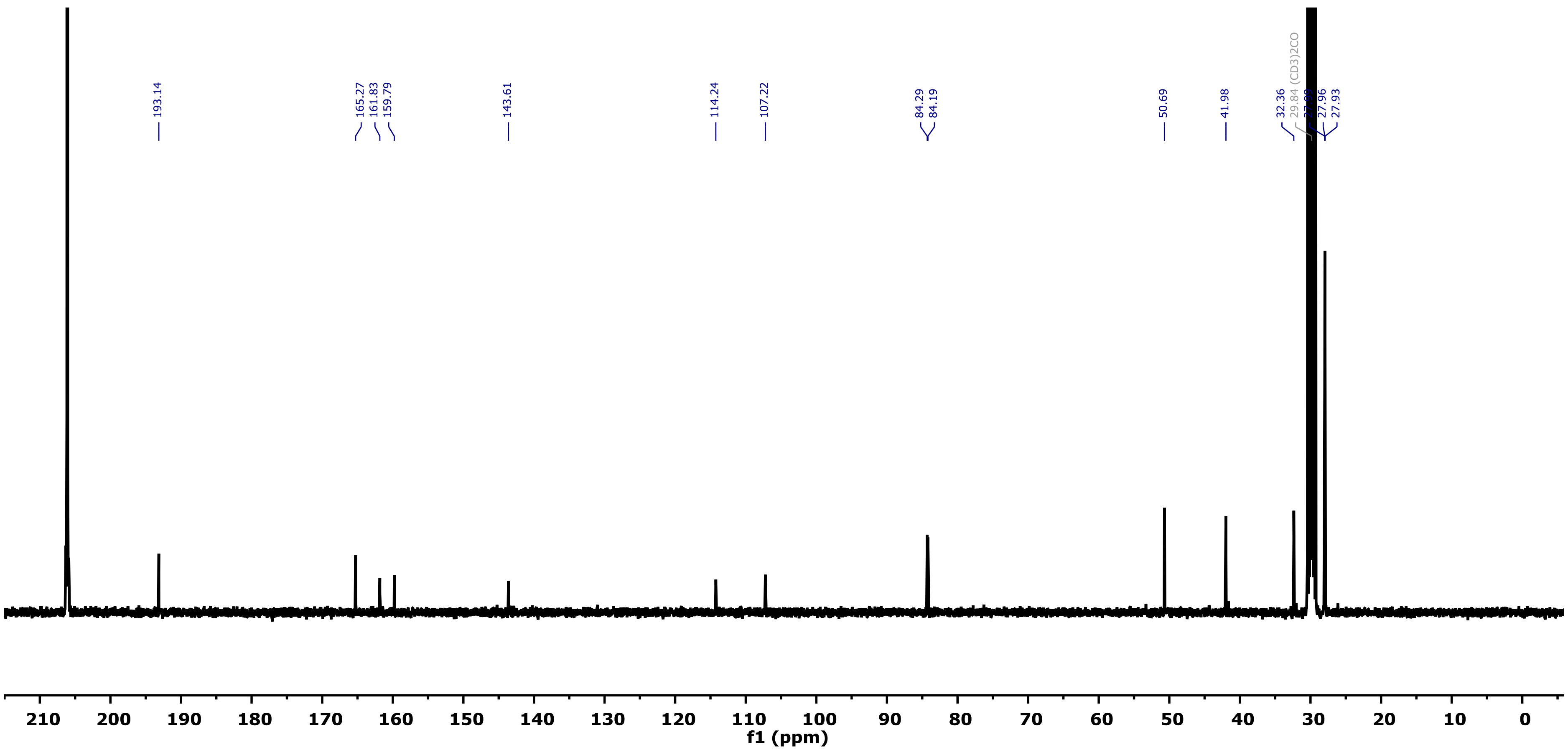

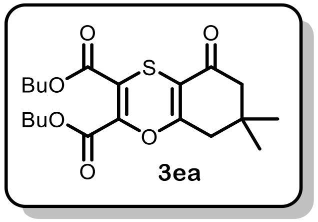

^{1}H NMR (Acetone d_6, 400 MHz)

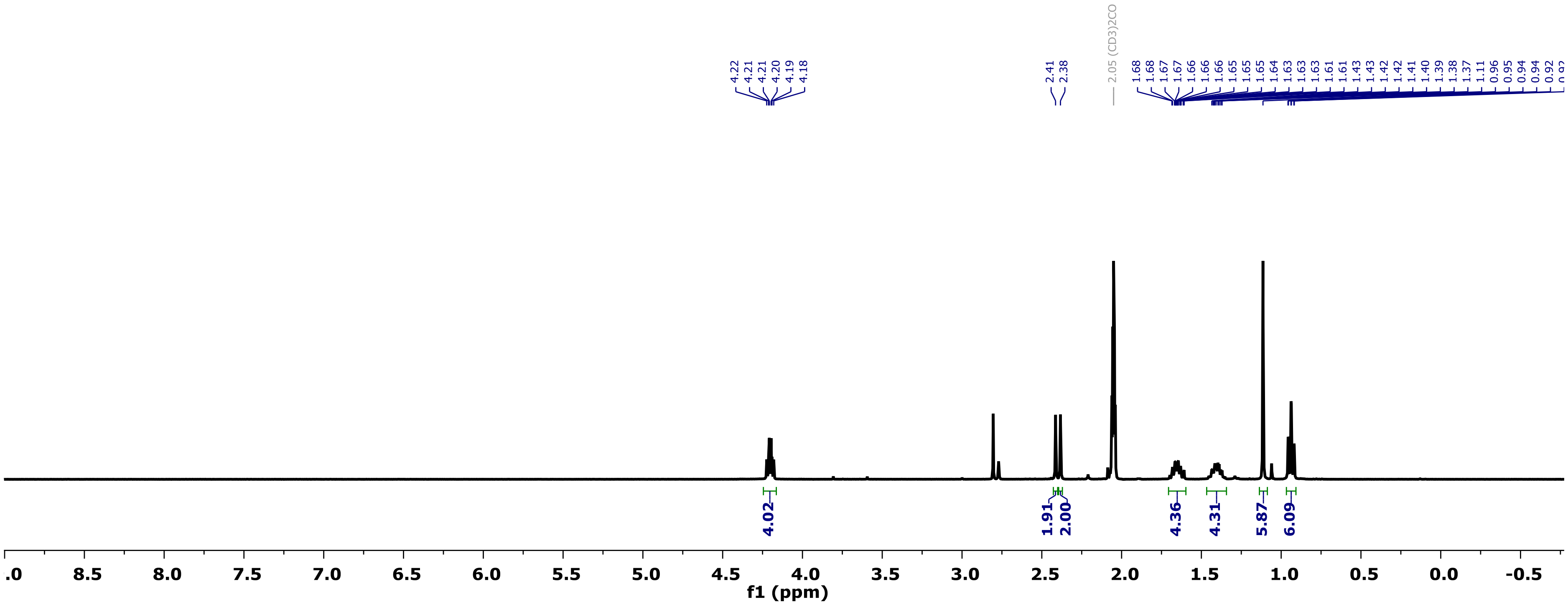

$^{13}C\{H\}$ NMR (Acetone d_6, 101 MHz)

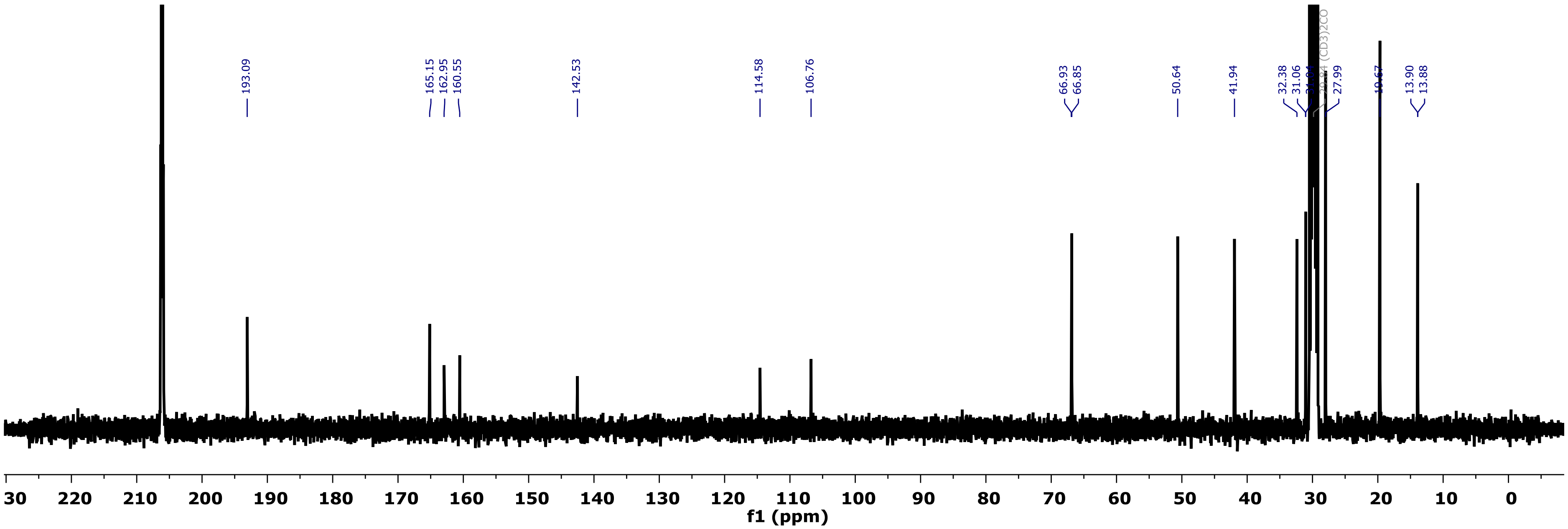

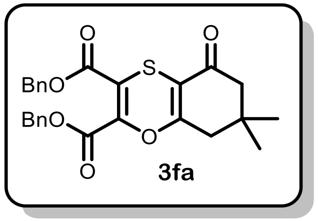

^{1}H NMR (Acetone d$_6$, 400 MHz)

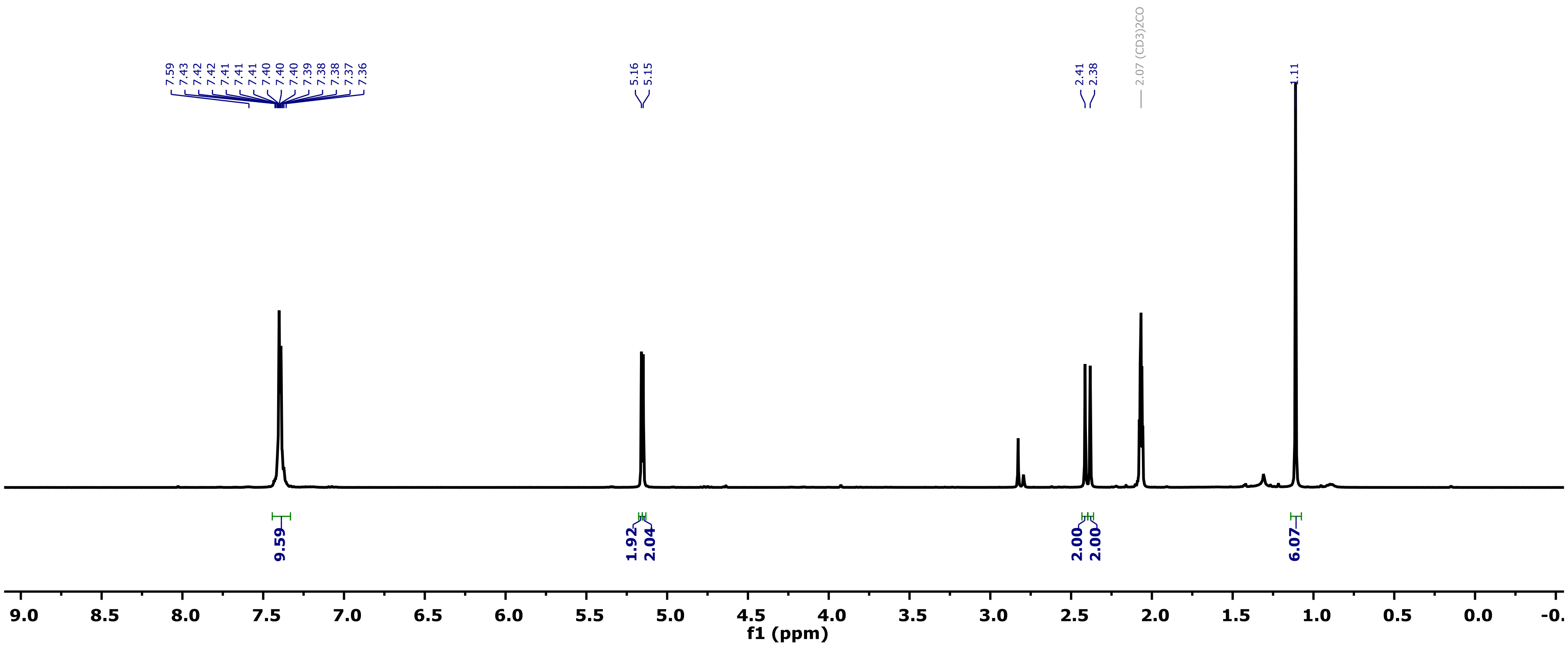

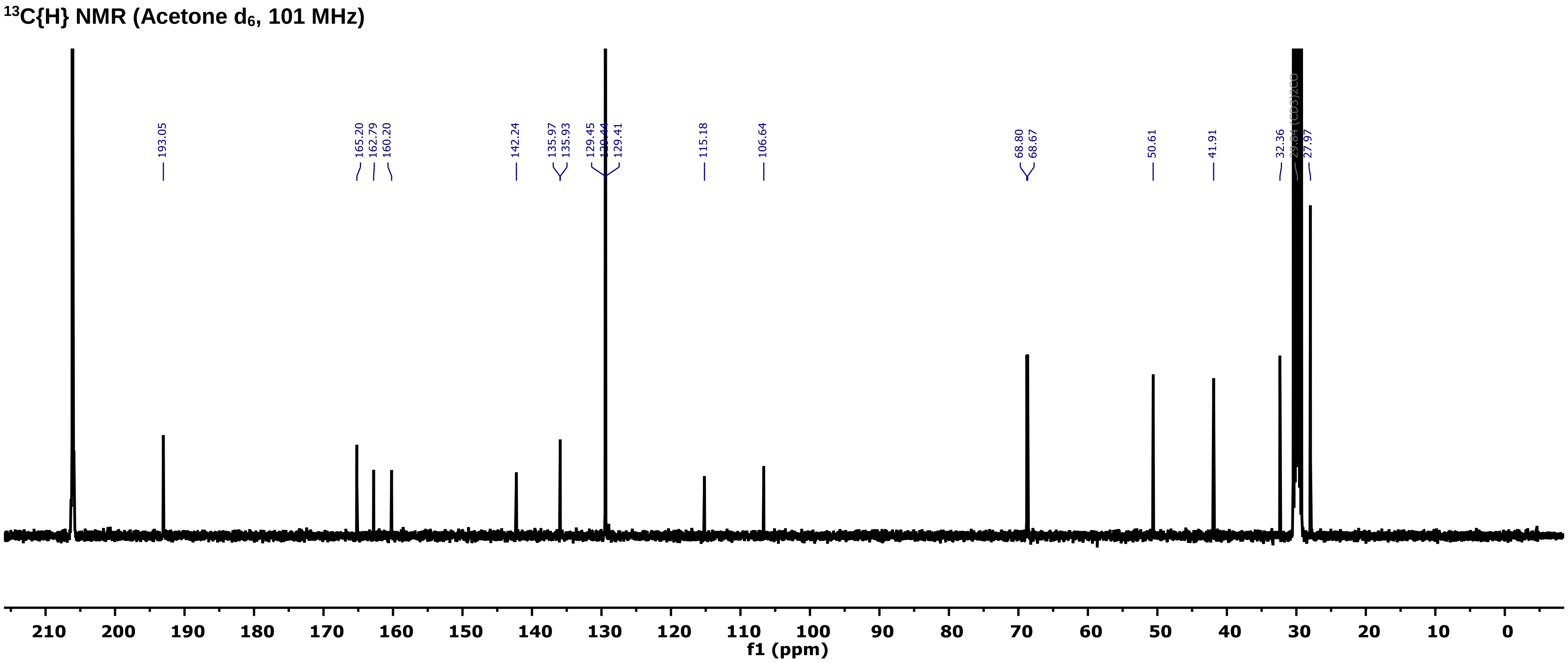

$^{13}C\{H\}$ NMR (Acetone d_6, 101 MHz)
193.05
165.20
162.79
160.20
142.24
135.97
135.93
129.45
129.44
129.41
115.18
106.64
68.80
68.67
50.61
41.91
32.36
29.84 (CD3)2CO
27.97
210
200
190
180
170
160
150
140
130
120
110
100
90
80
70
60
50
40
30
20
10
0
f1 (ppm)

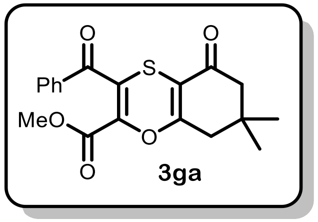

^{1}H NMR (Acetone d_6, 400 MHz)

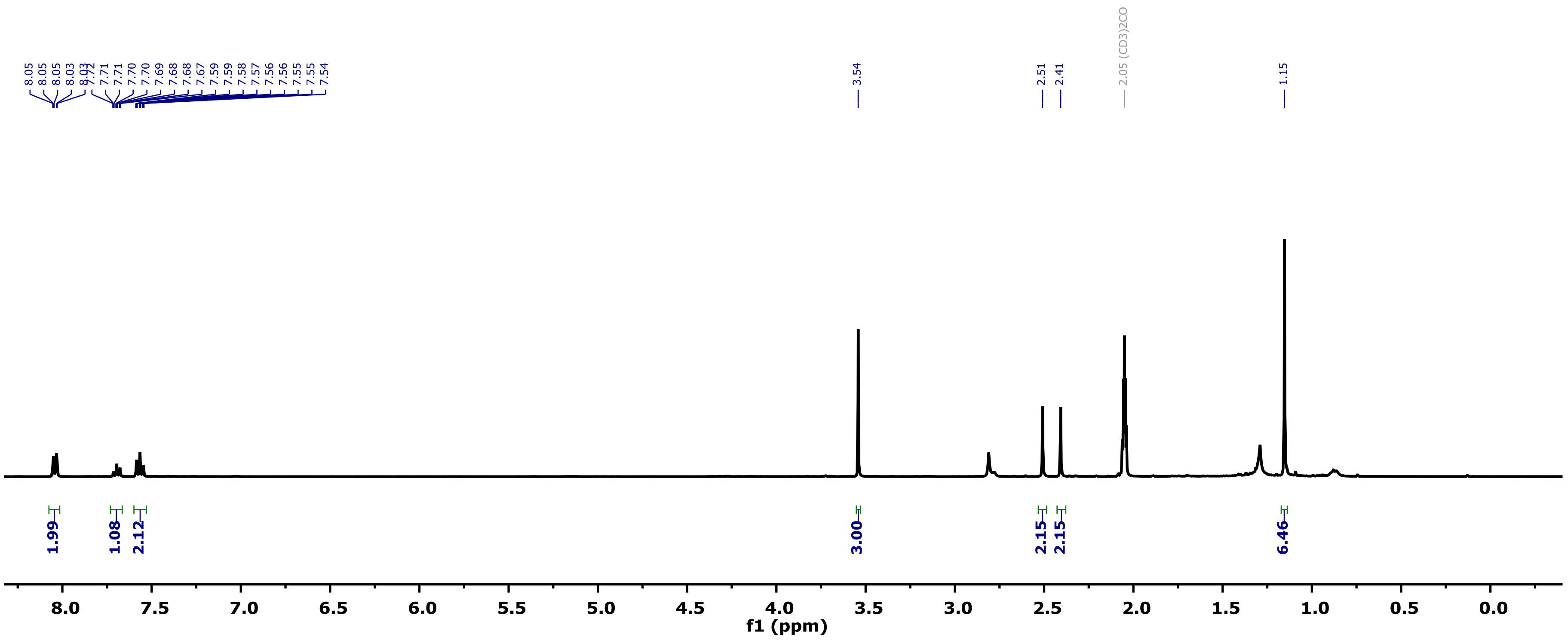

$^{13}C\{H\}$ NMR (Acetone d_6, 101 MHz)

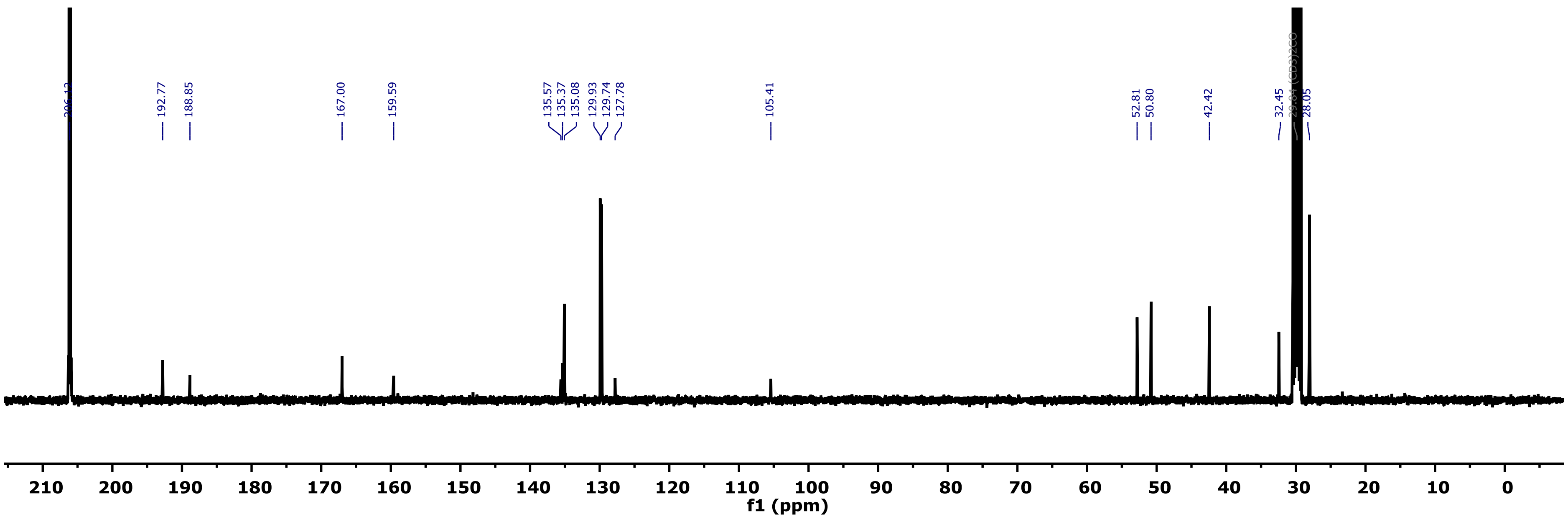

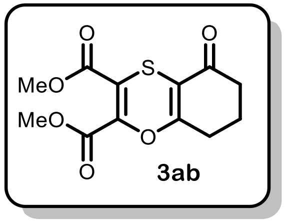

^{1}H NMR (Acetone d_6, 400 MHz)

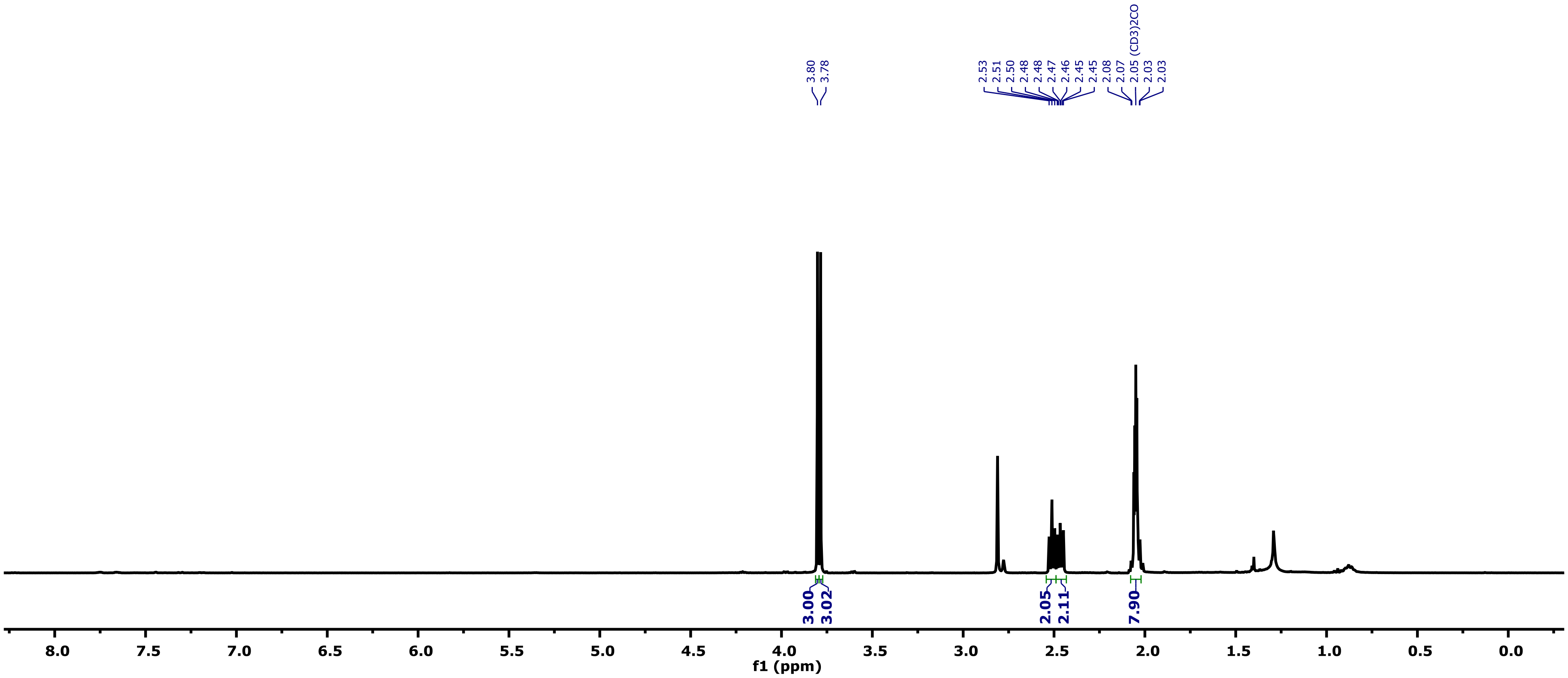

^{13}C{H} NMR (Acetone d_6, 101 MHz)

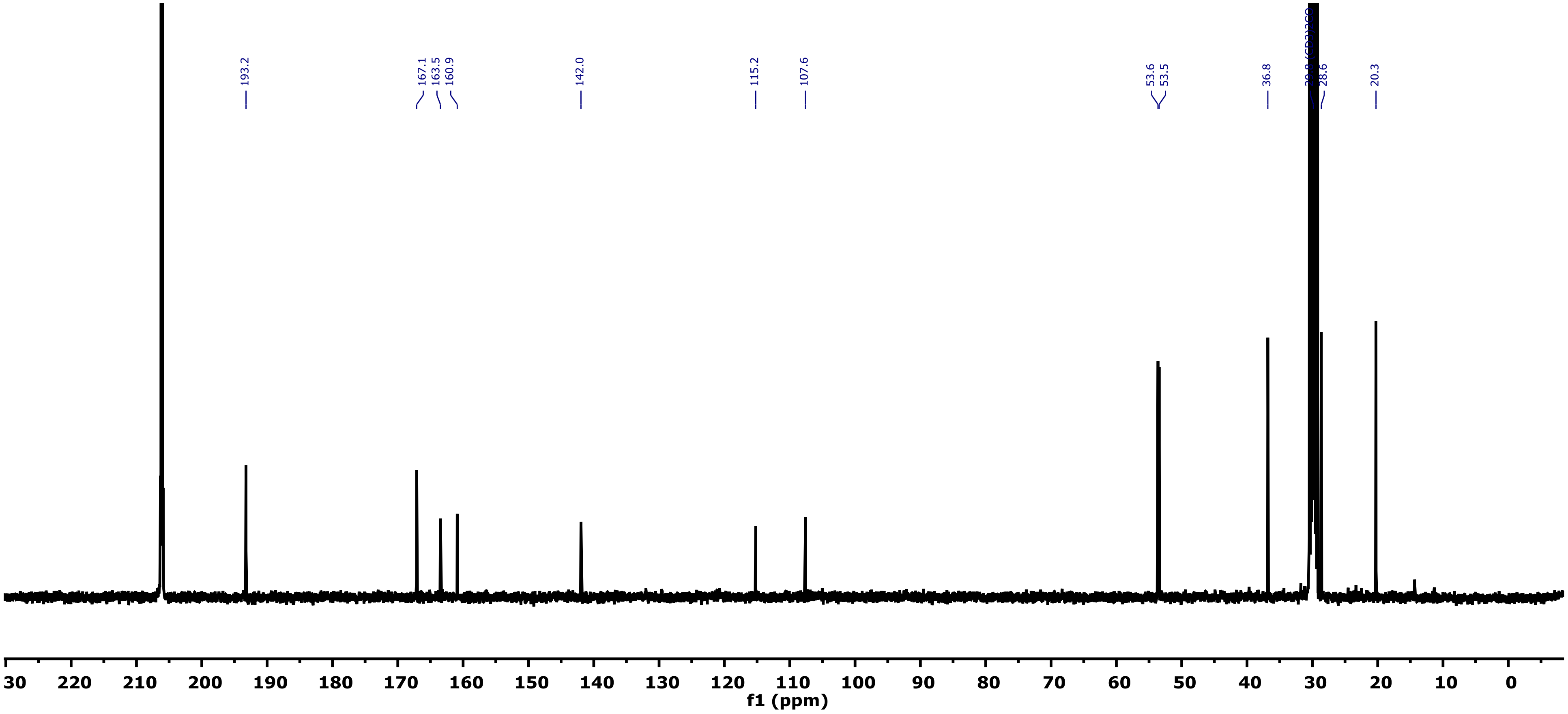

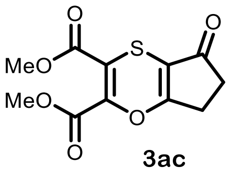

3ac

^{1}H NMR (Acetone d$_6$, 400 MHz)

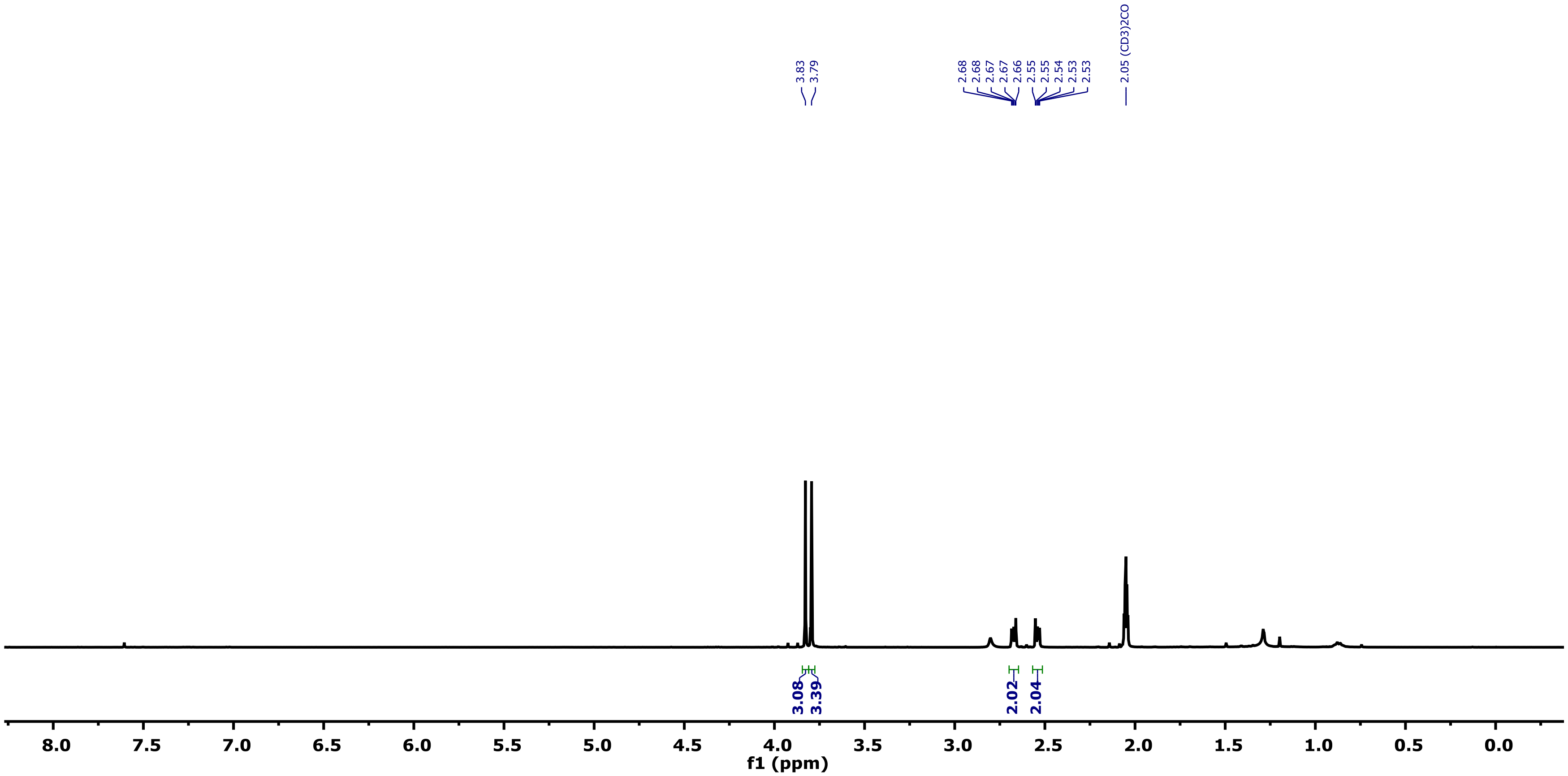

$^{13}C\{H\}$ NMR (Acetone d_6, 101 MHz)

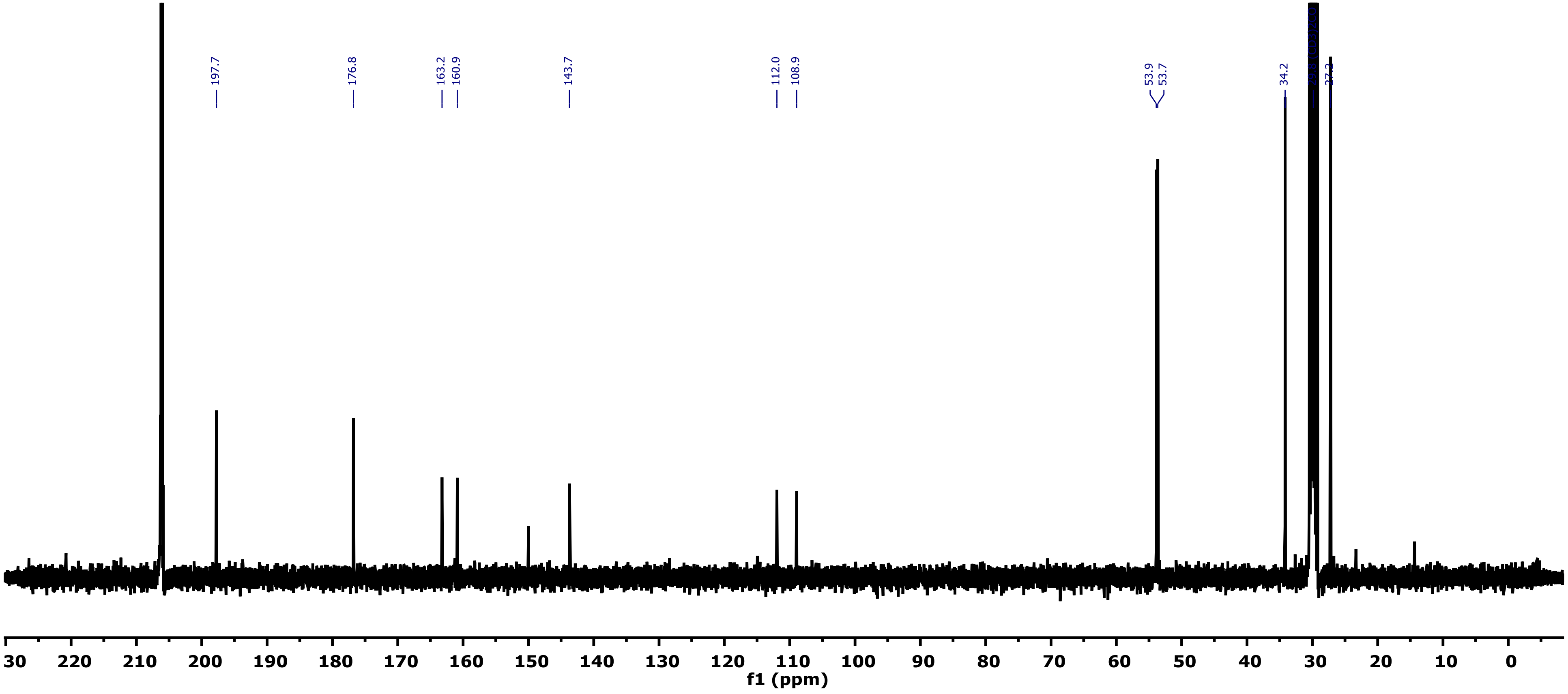

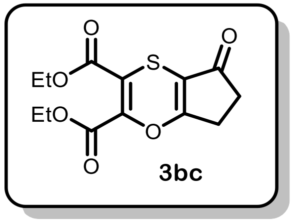

3bc

^{1}H NMR (Acetone d_6, 400 MHz)

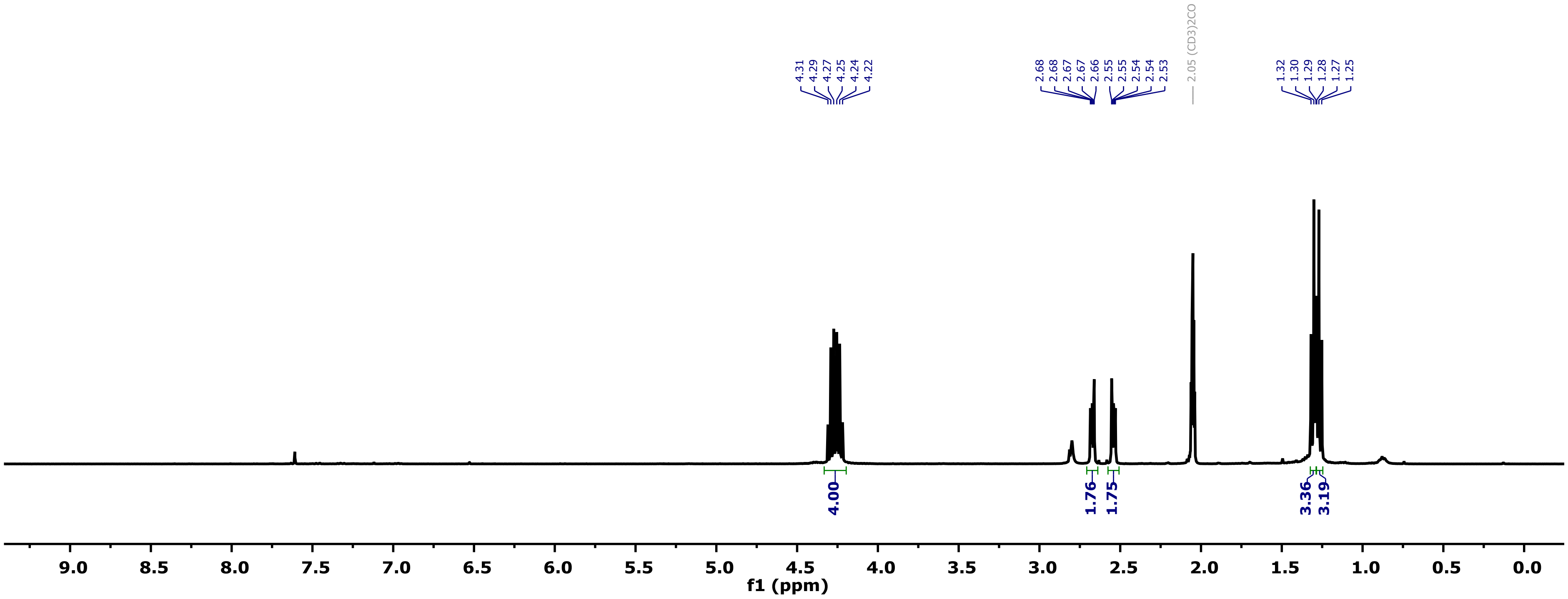

$^{13}C\{H\}$ NMR (Acetone d_6, 101 MHz)

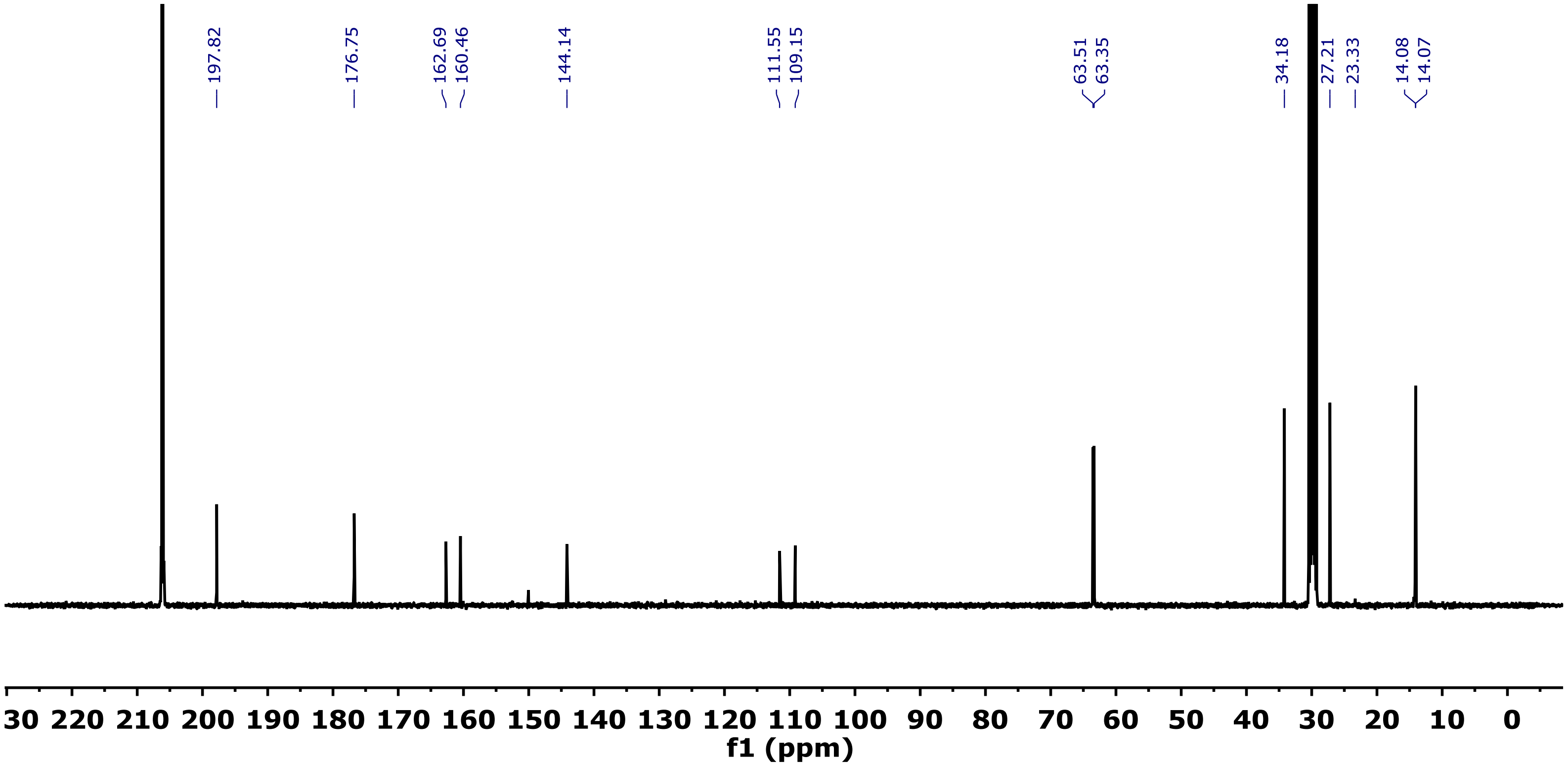

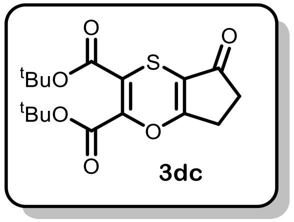

^{1}H NMR (Acetone d$_6$, 400 MHz)

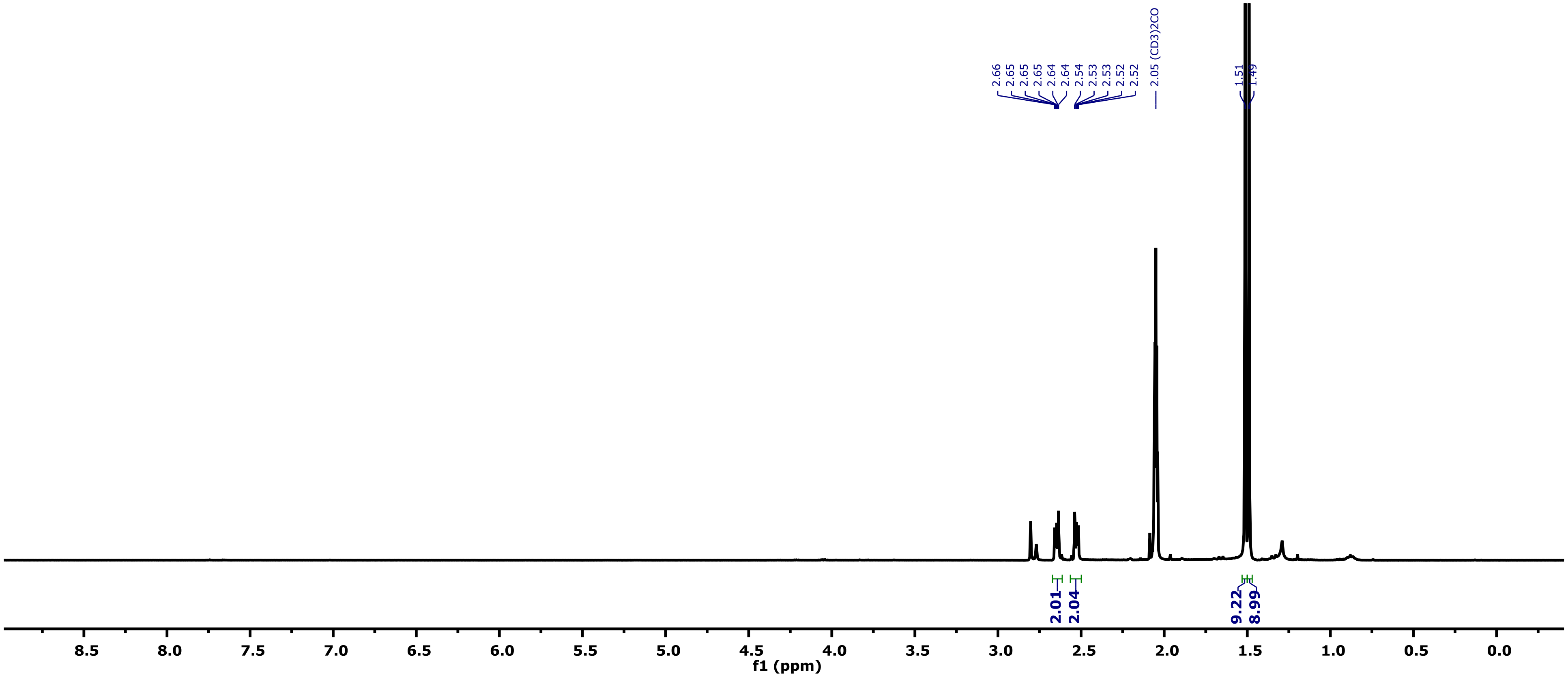

$^{13}C\{H\}$ NMR (Acetone d_6, 101 MHz)

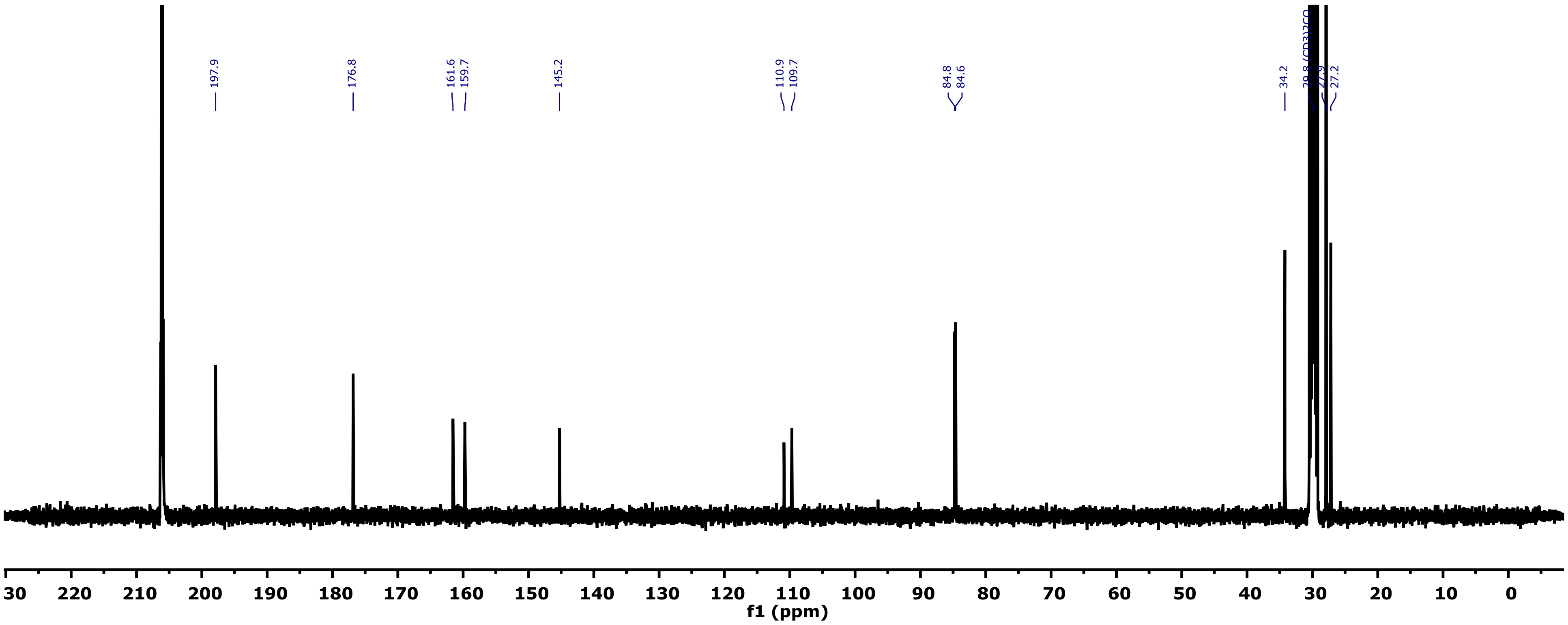

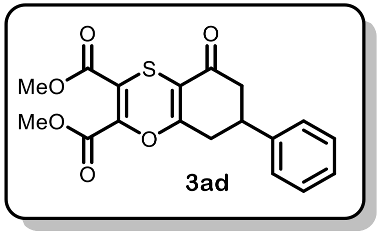

^{1}H NMR (Acetone d_6, 400 MHz)

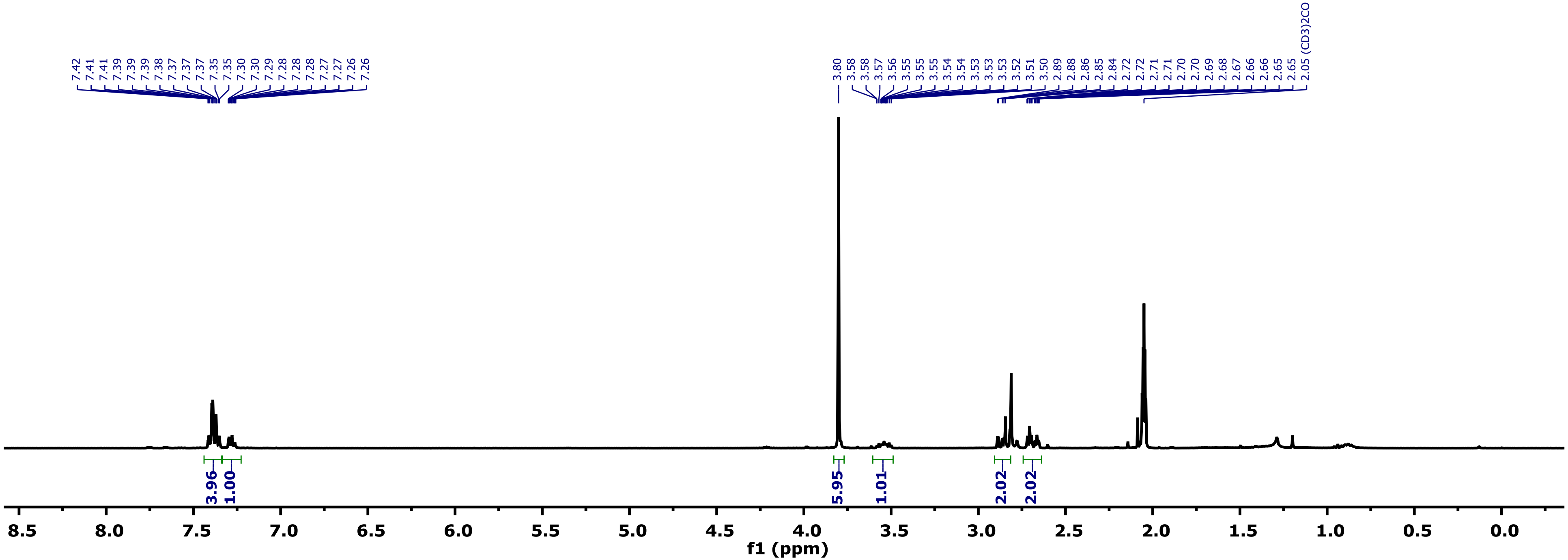

^{13}C{H} NMR (Acetone d_6, 101 MHz)

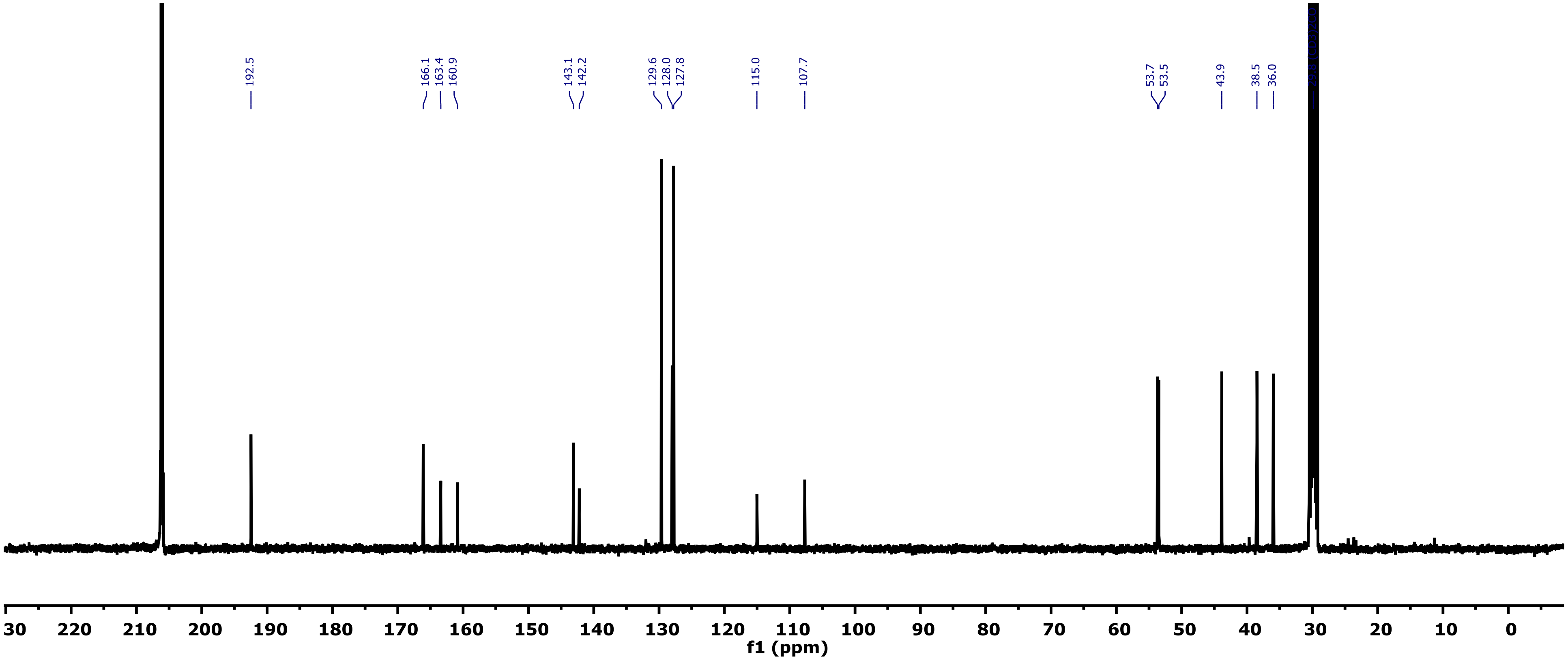

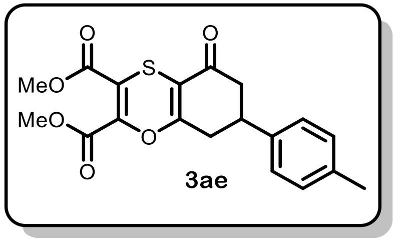

^{1}H NMR (Acetone d$_6$, 400 MHz)

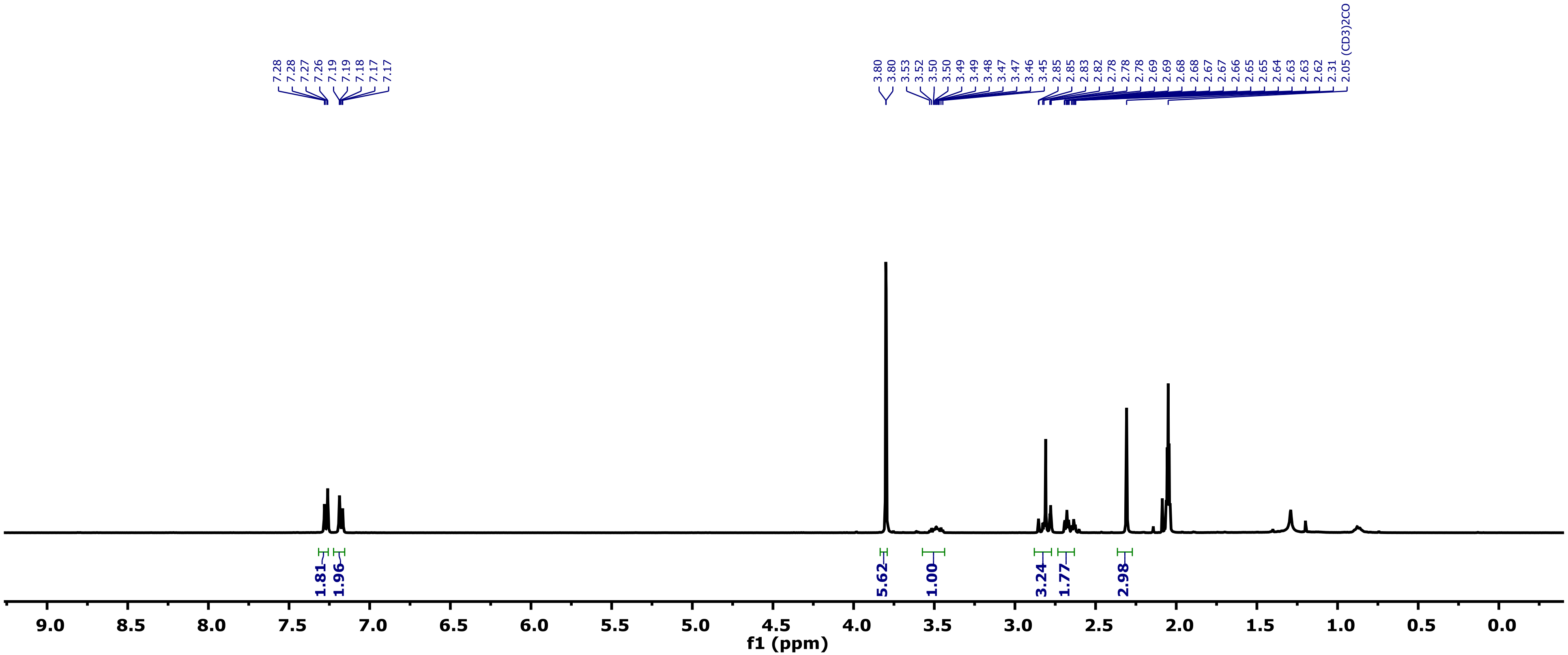

$^{13}C\{H\}$ NMR (Acetone d_6, 101 MHz)

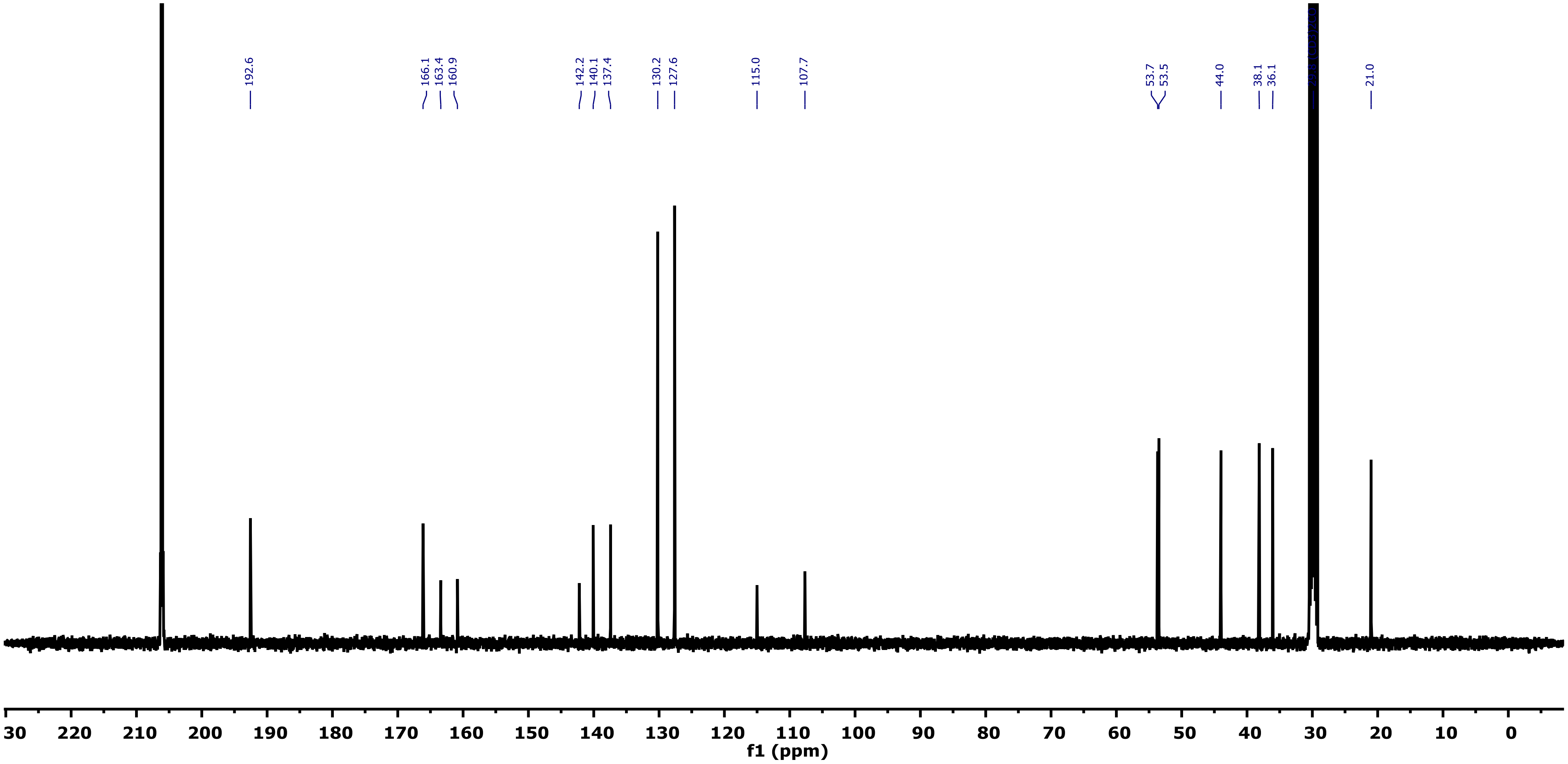

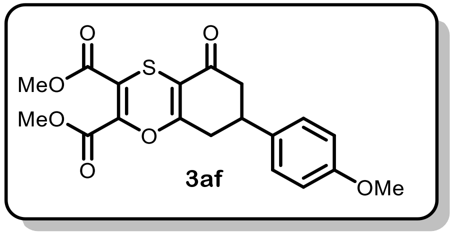

^{1}H NMR (Acetone d_6, 400 MHz)

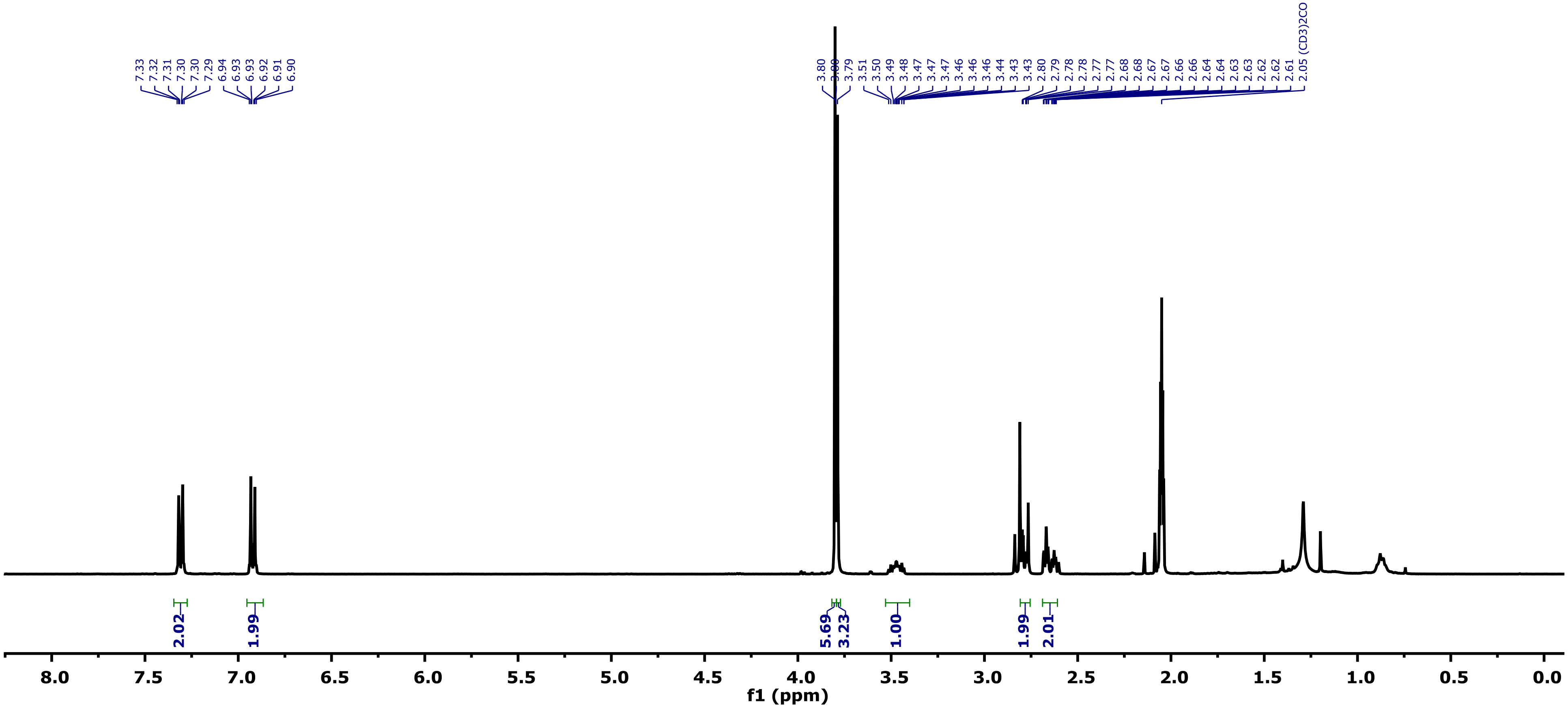

$^{13}C\{H\}$ NMR (Acetone d_6, 101 MHz)

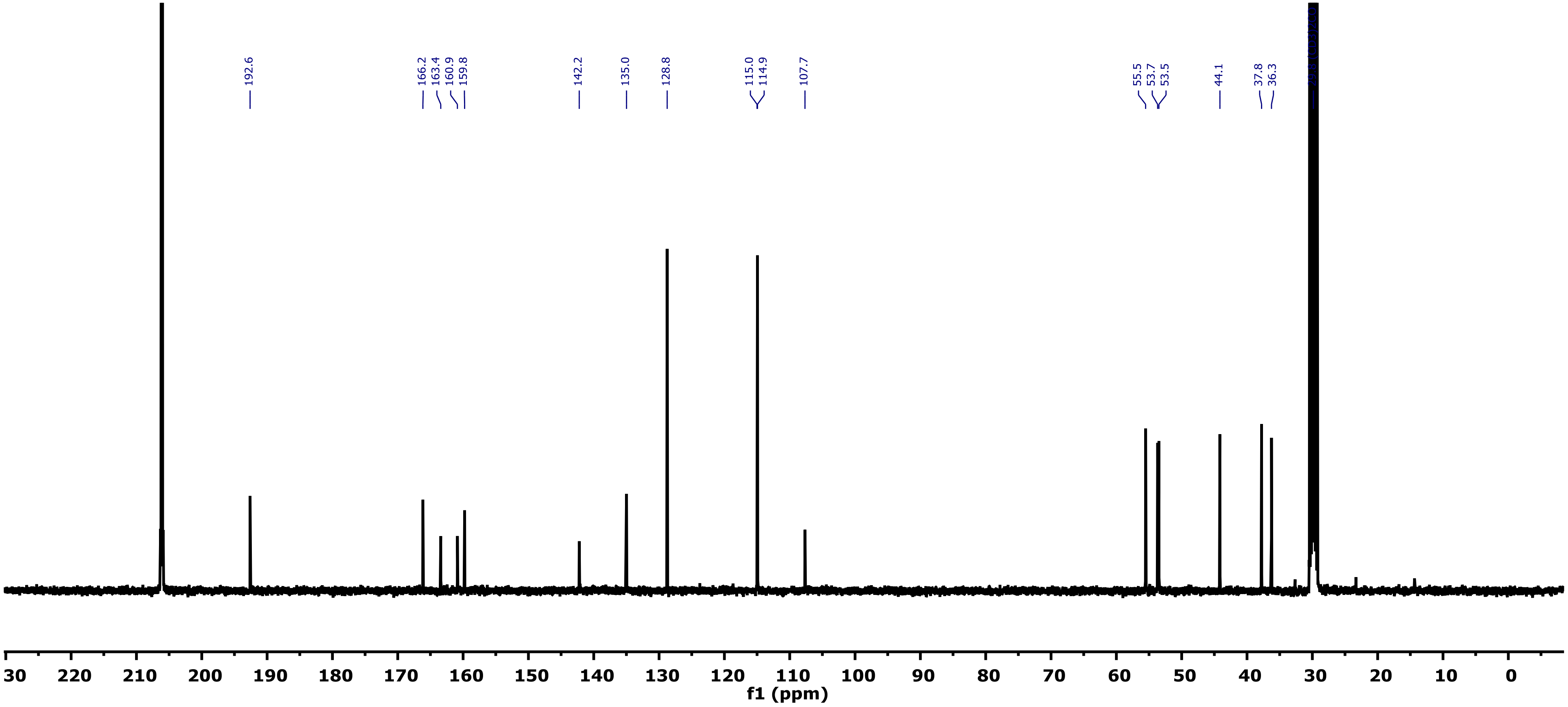

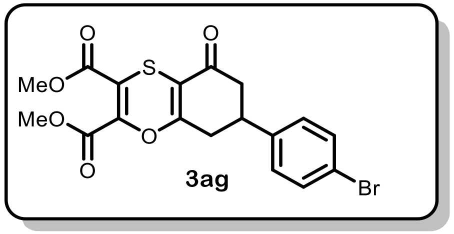

^{1}H NMR (Acetone d_6, 400 MHz)

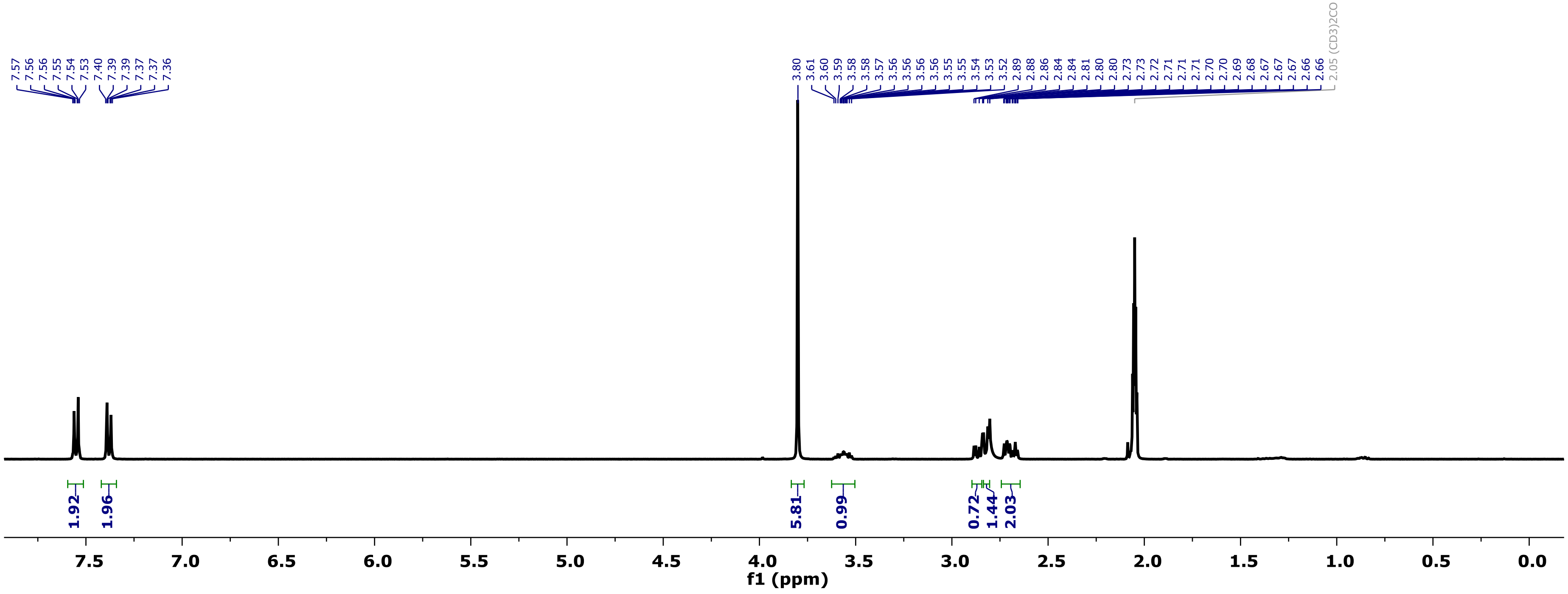

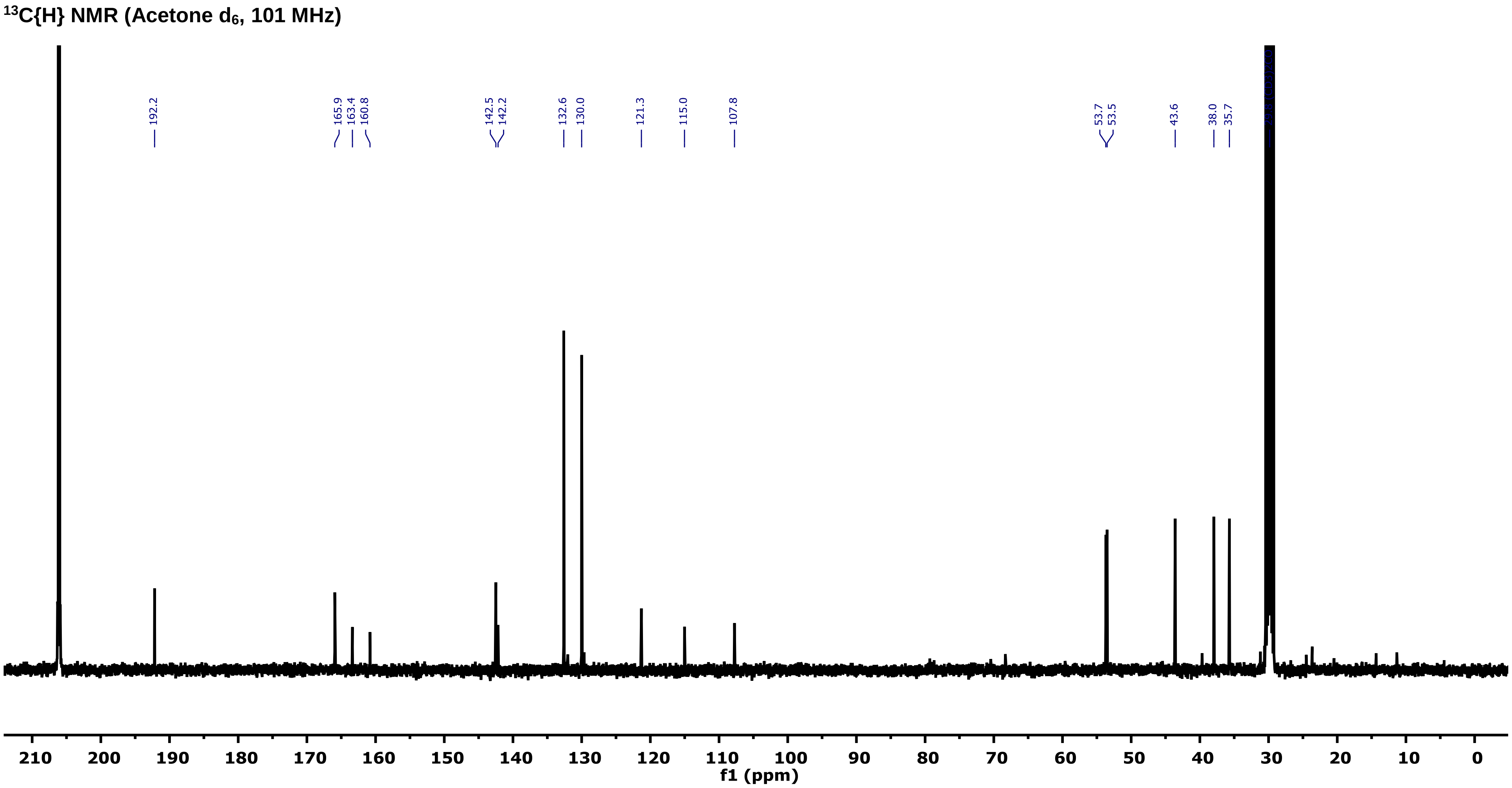

$^{13}C\{H\}$ NMR (Acetone d_6, 101 MHz)
192.2
165.9
163.4
160.8
142.5
142.2
132.6
130.0
121.3
115.0
107.8
53.7
53.5
43.6
38.0
35.7
210 200 190 180 170 160 150 140 130 120 110 100 90 80 70 60 50 40 30 20 10 0
f1 (ppm)

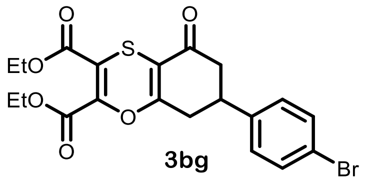

3bg

^{1}H NMR (Acetone d_6, 400 MHz)

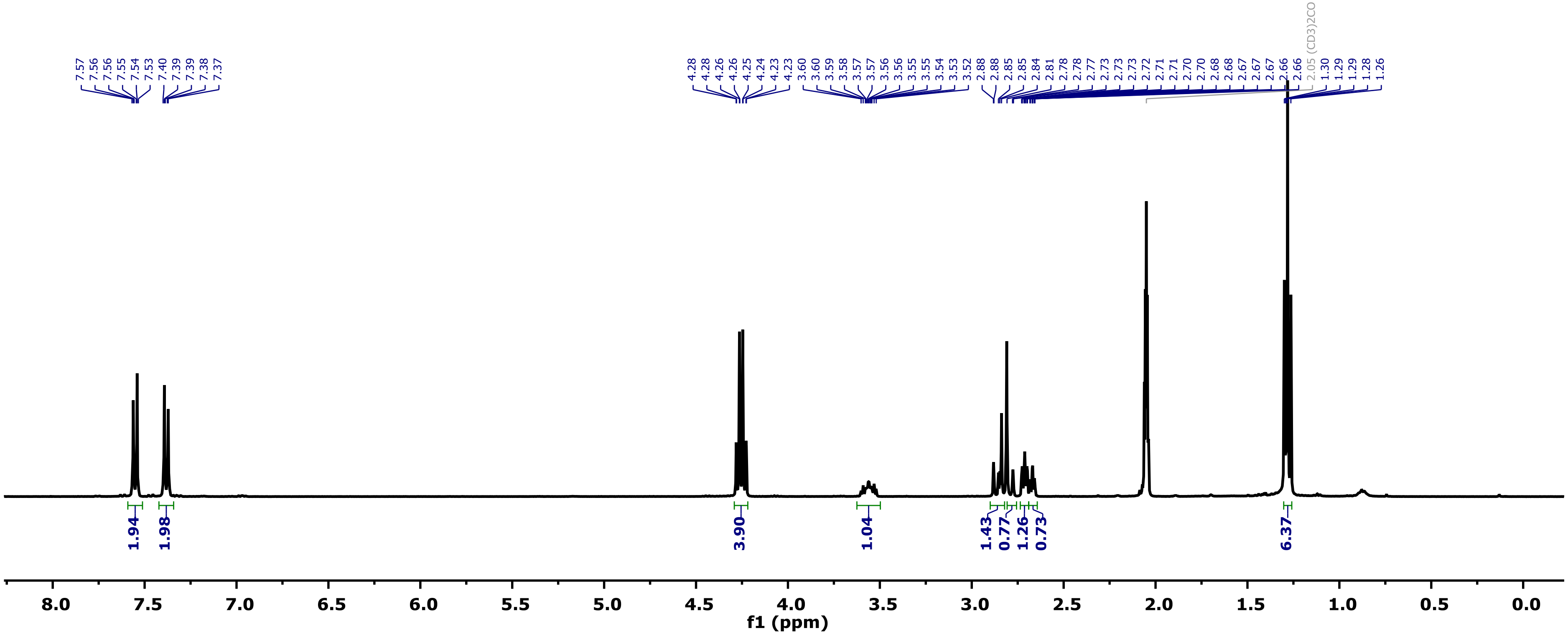

$^{13}C\{H\}$ NMR (Acetone d_6, 101 MHz)

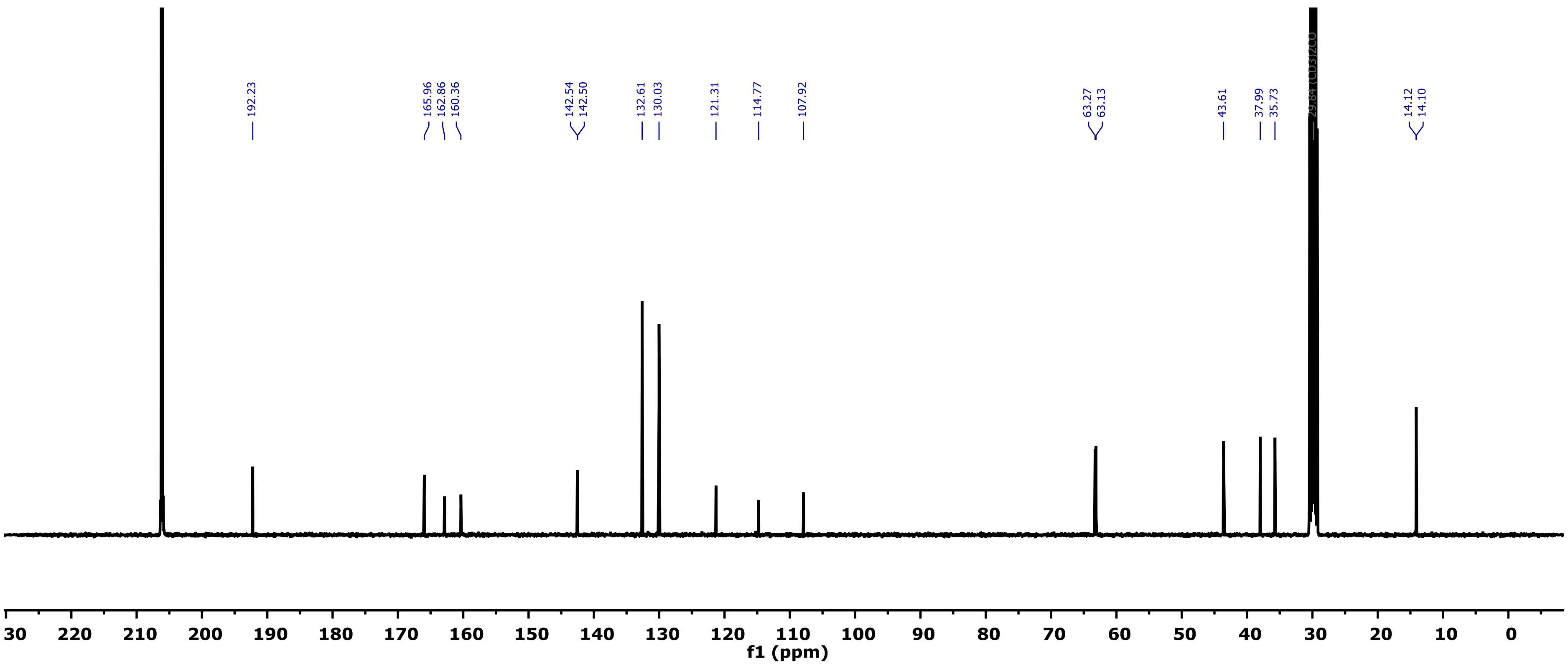

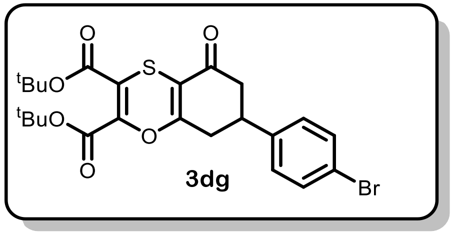

^{1}H NMR (Acetone d$_6$, 400 MHz)

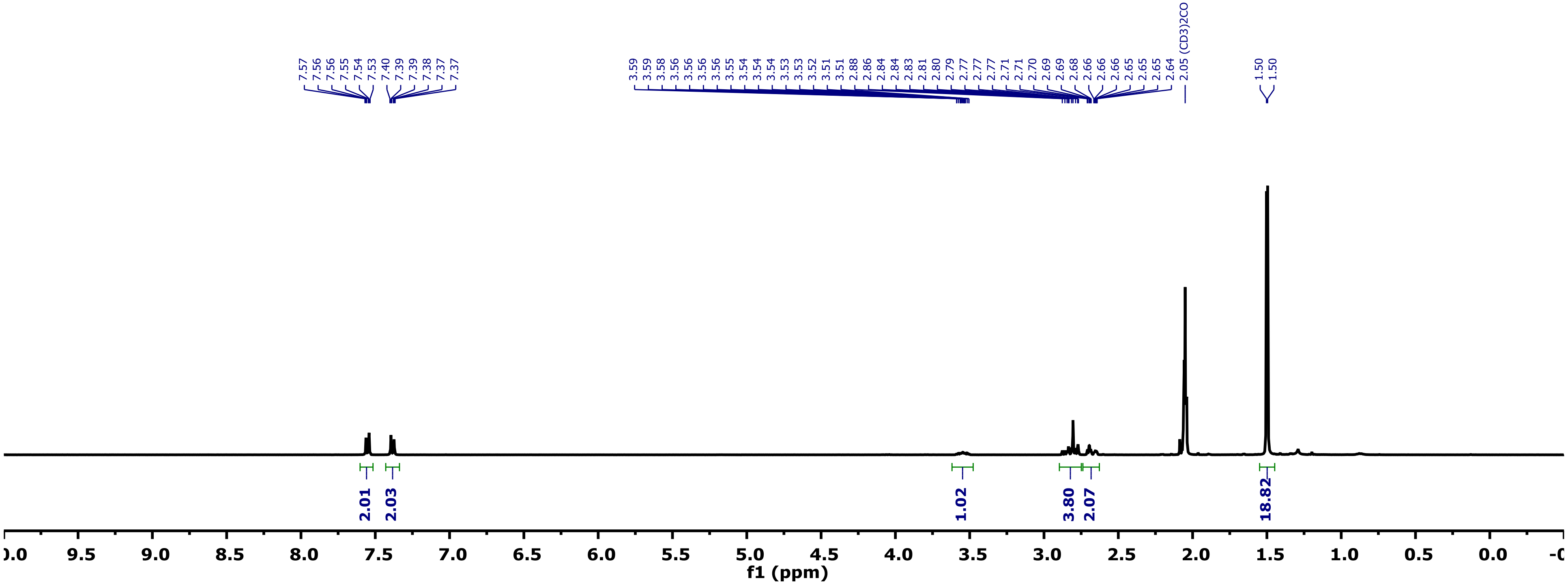

$^{13}C\{H\}$ NMR (Acetone d_6, 101 MHz)

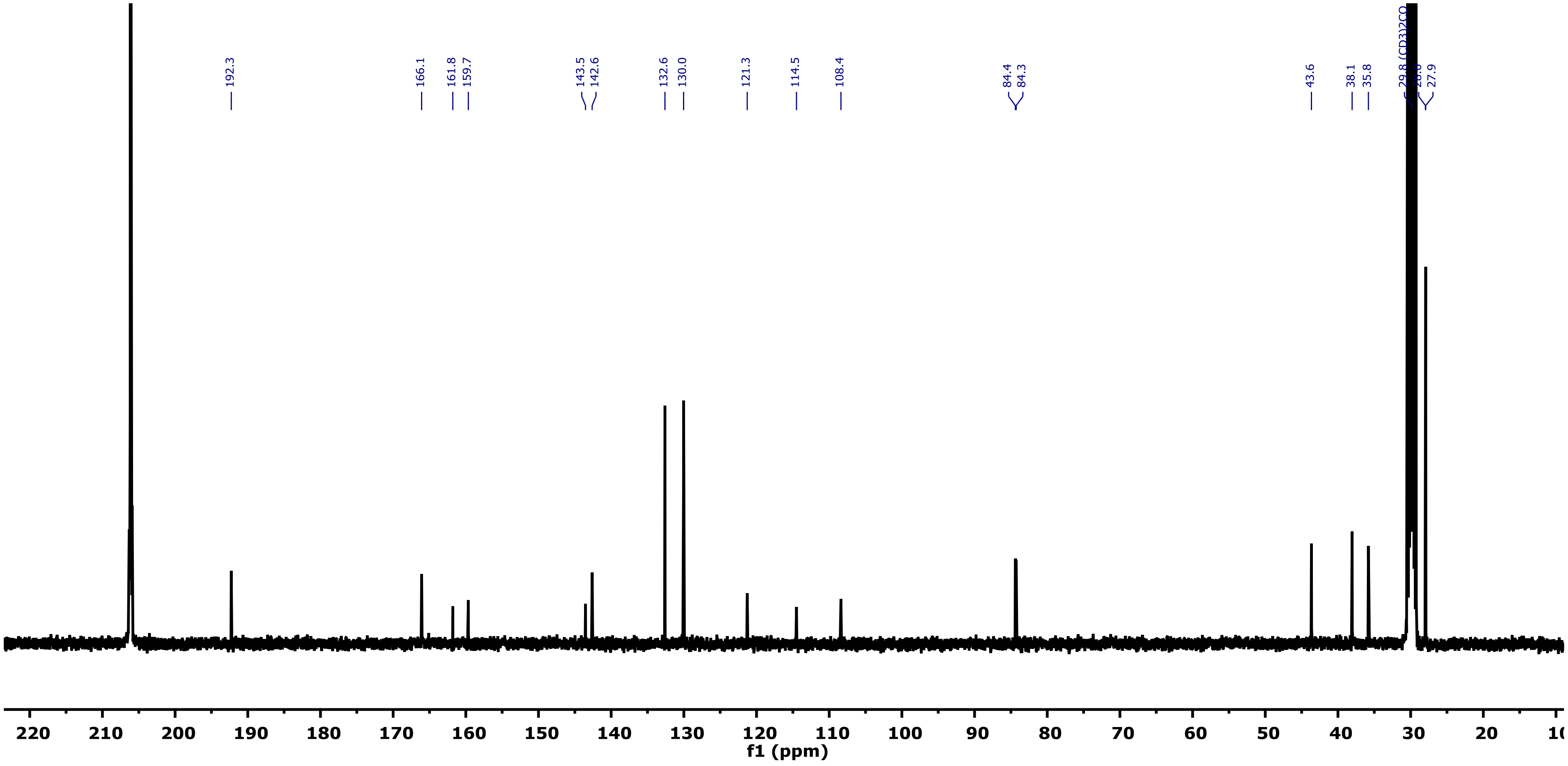

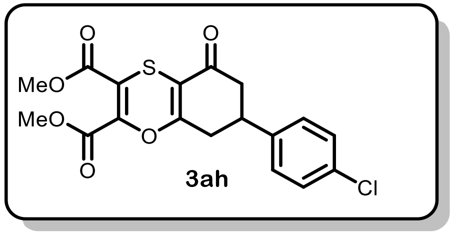

^{1}H NMR (Acetone d_6, 400 MHz)

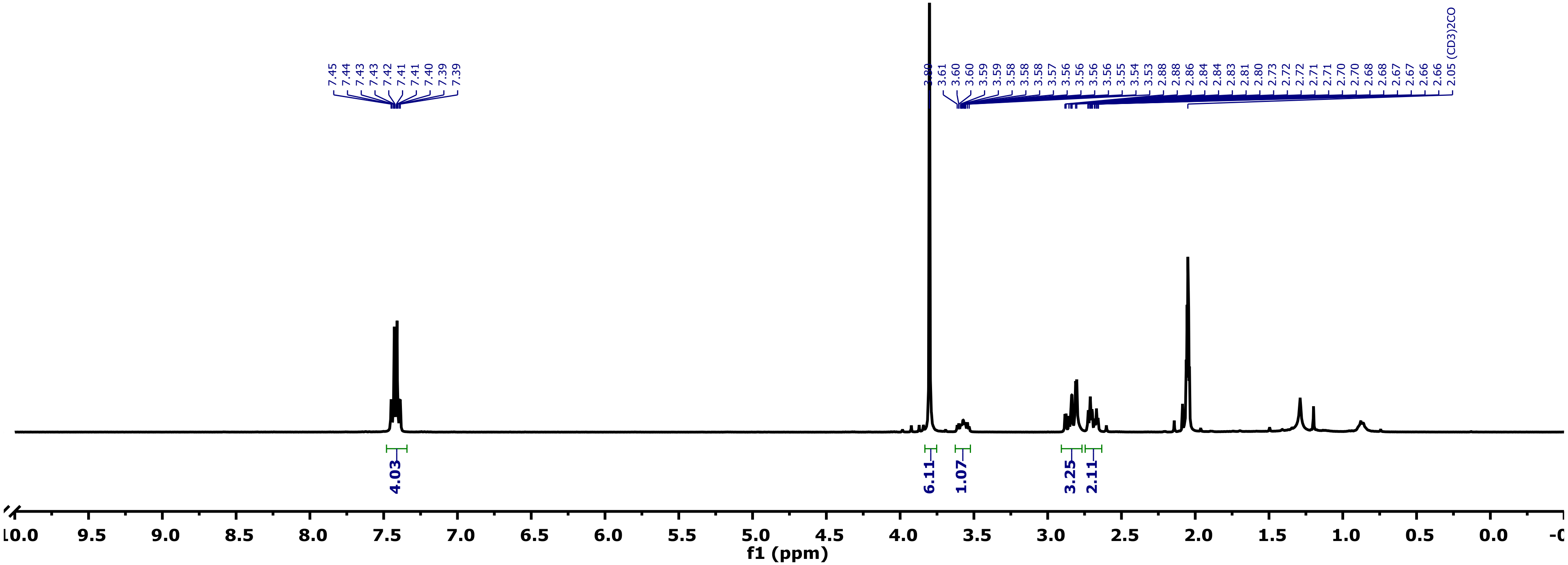

$^{13}C\{H\}$ NMR (Acetone d_6, 101 MHz)

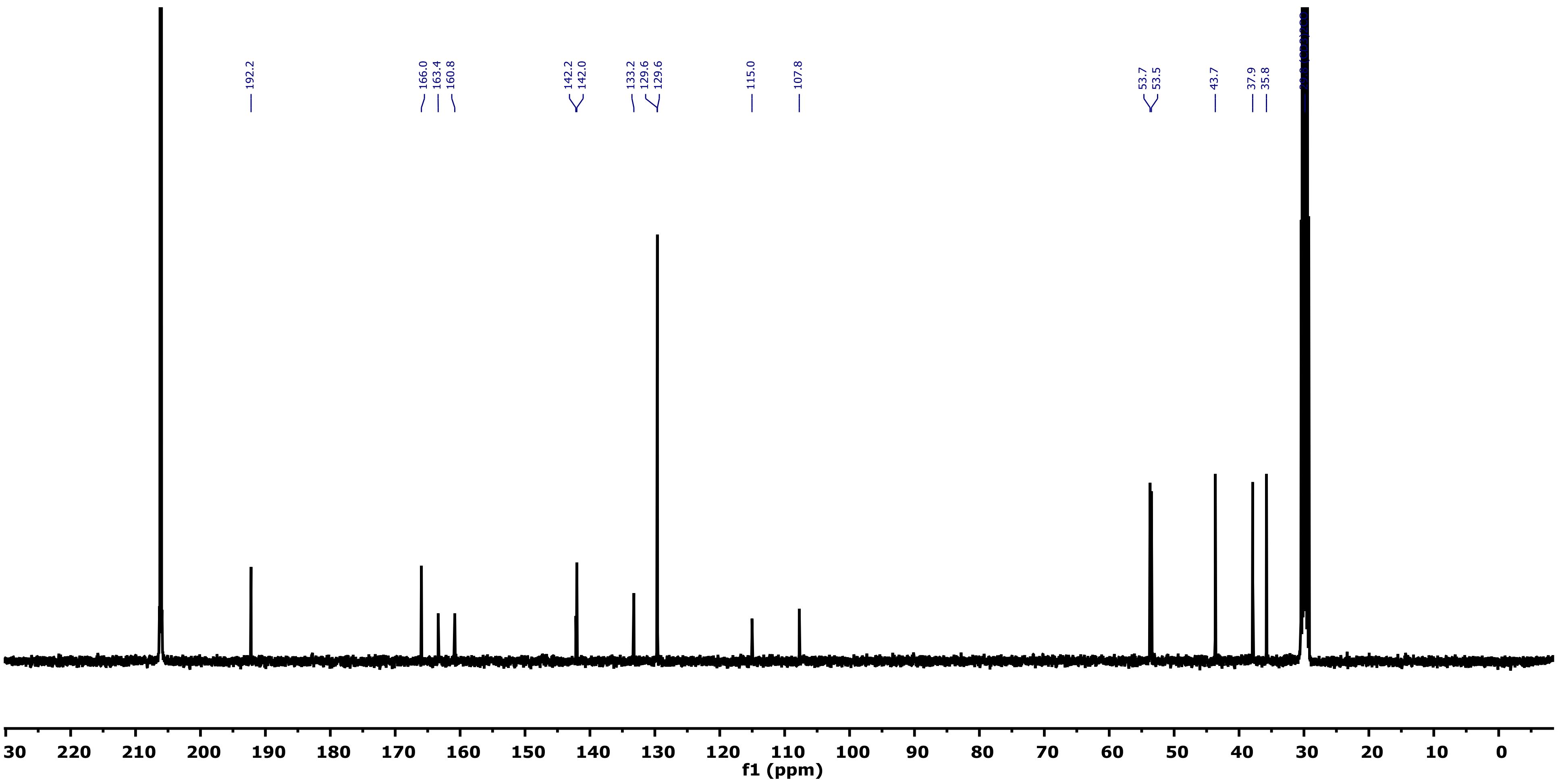

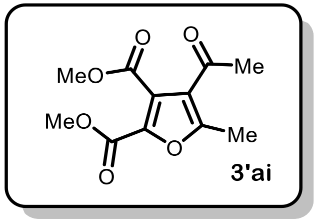

^{1}H NMR (Acetone d_6, 400 MHz)

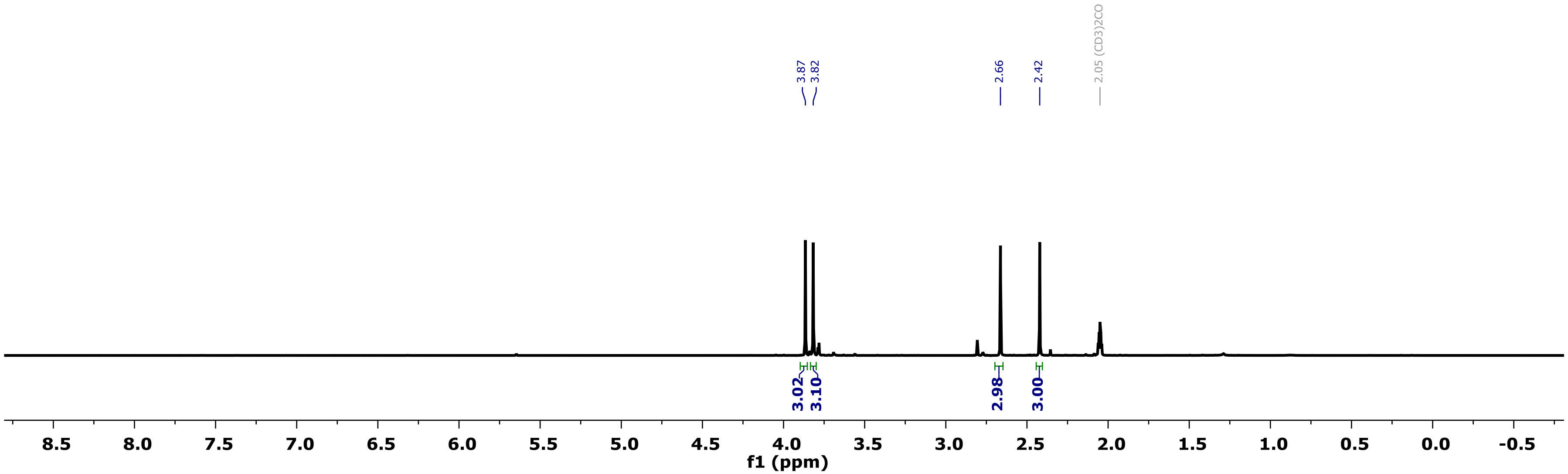

$^{13}C\{H\}$ NMR (Acetone d_6, 101 MHz)

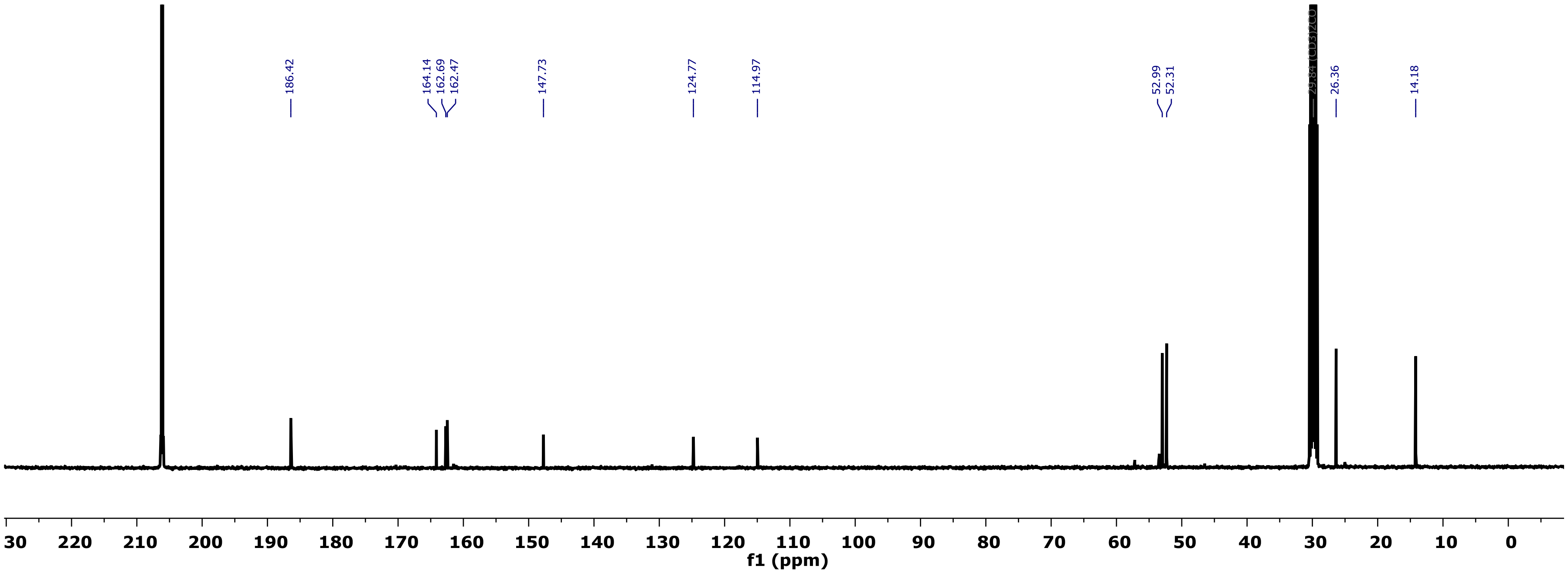

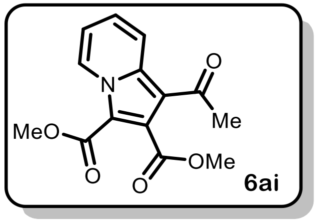

6ai

^{1}H NMR (CDCl$_3$, 400 MHz)

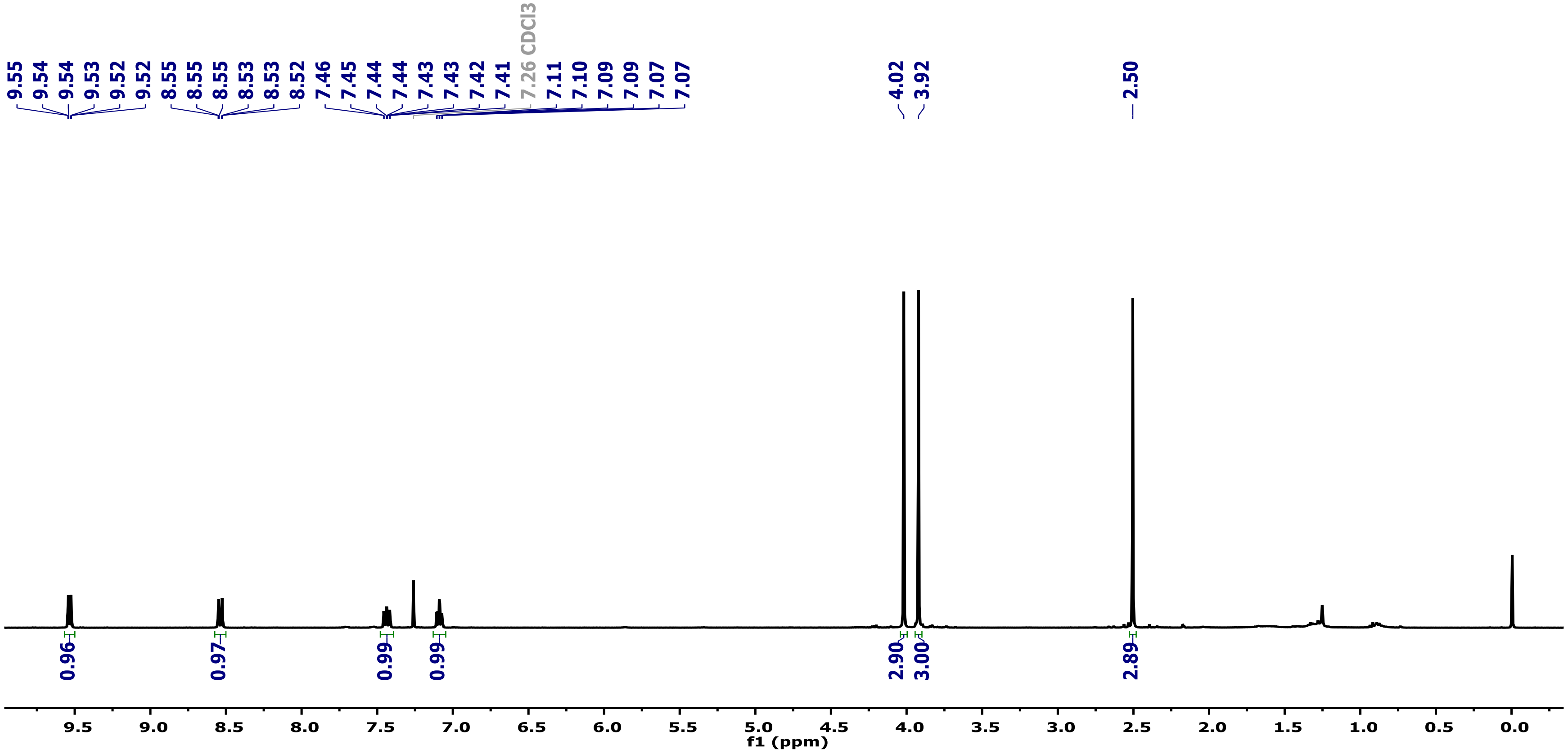

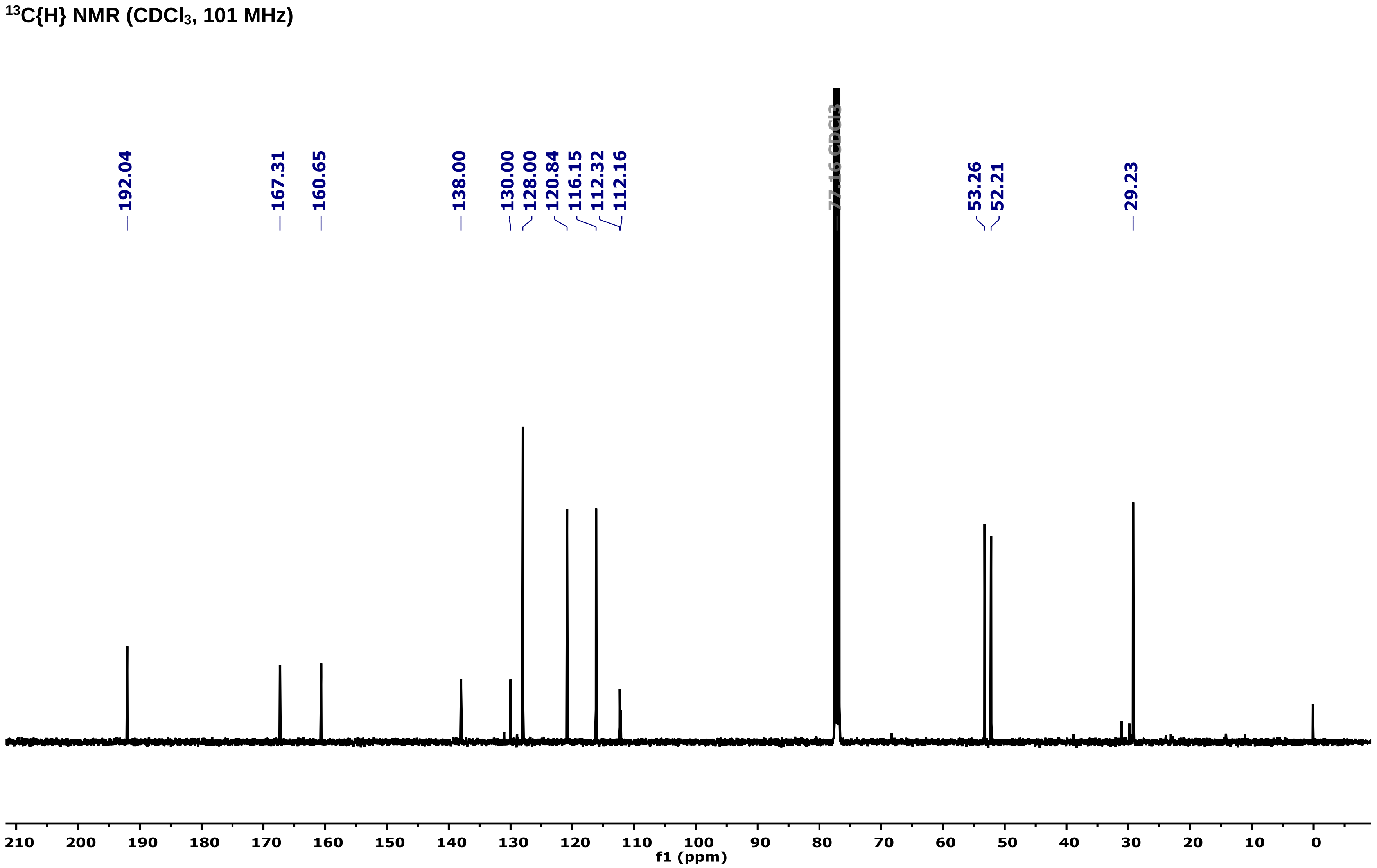

13C{H} NMR (CDCl3, 101 MHz)
192.04
167.31
160.65
138.00
130.00
128.00
120.84
116.15
112.32
112.16
77.16 CDCl3
53.26
52.21
29.23
210 200 190 180 170 160 150 140 130 120 110 100 90 80 70 60 50 40 30 20 10 0
f1 (ppm)

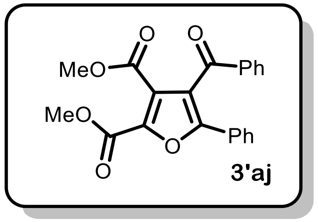

^{1}H NMR (Acetone d$_6$, 400 MHz)

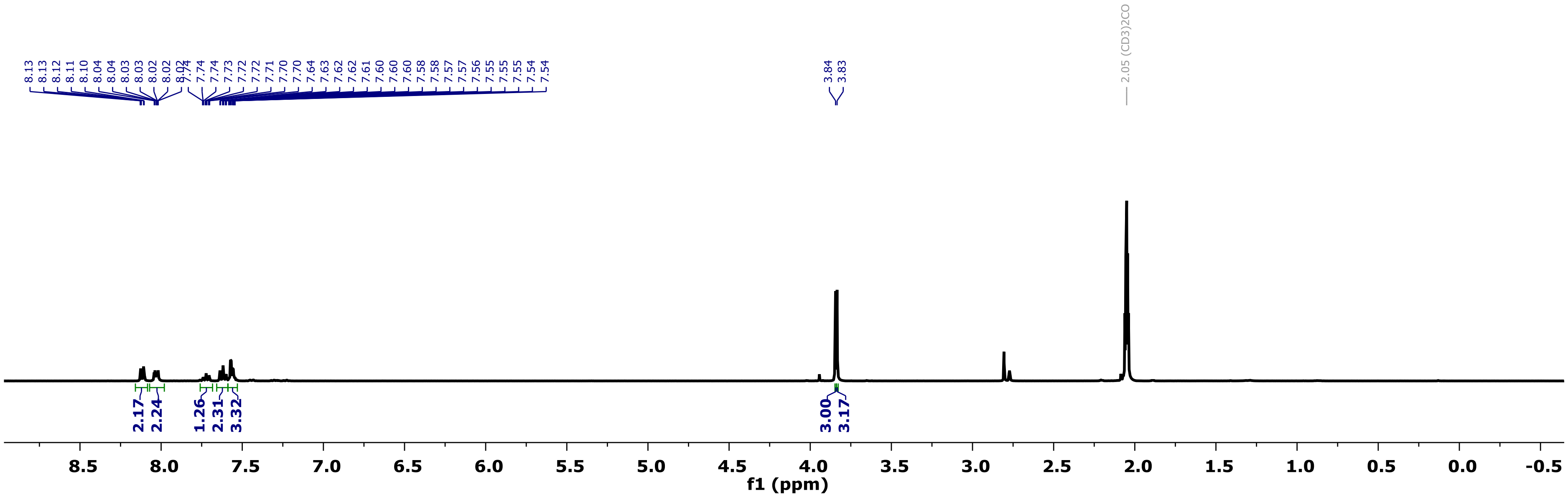

$^{13}C\{H\}$ NMR (Acetone d_6, 101 MHz)

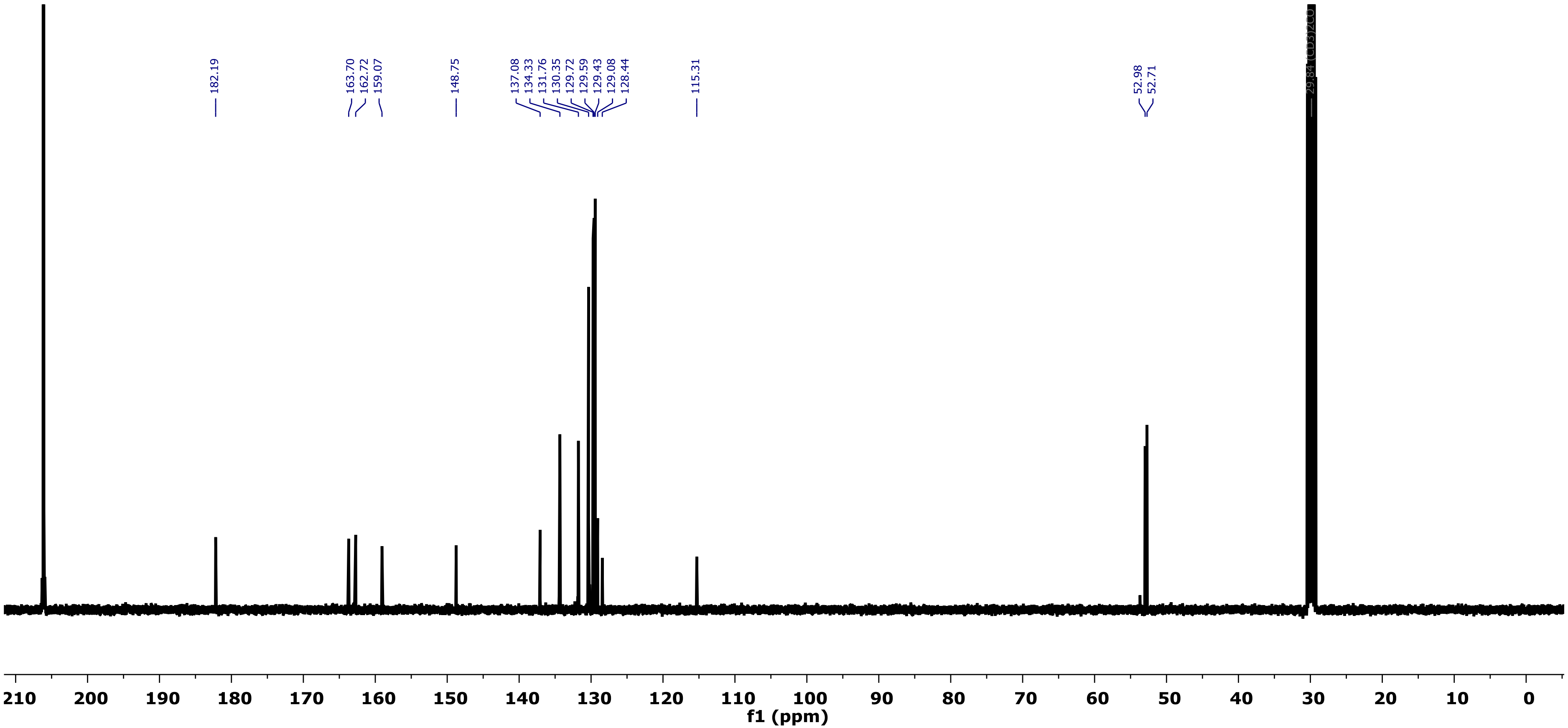

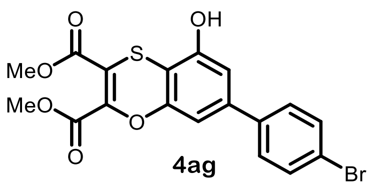

4ag

^{1}H NMR (Acetone d$_6$, 400 MHz)

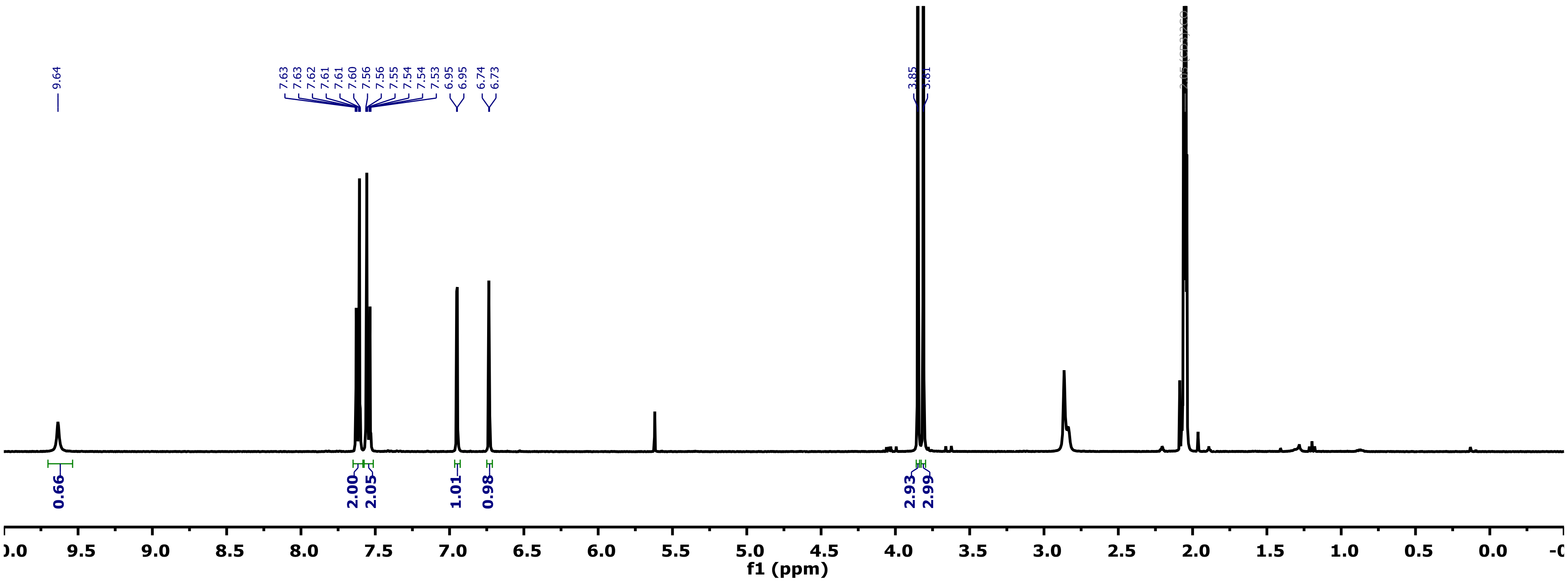

$^{13}C\{H\}$ NMR (Acetone d_6, 101 MHz)

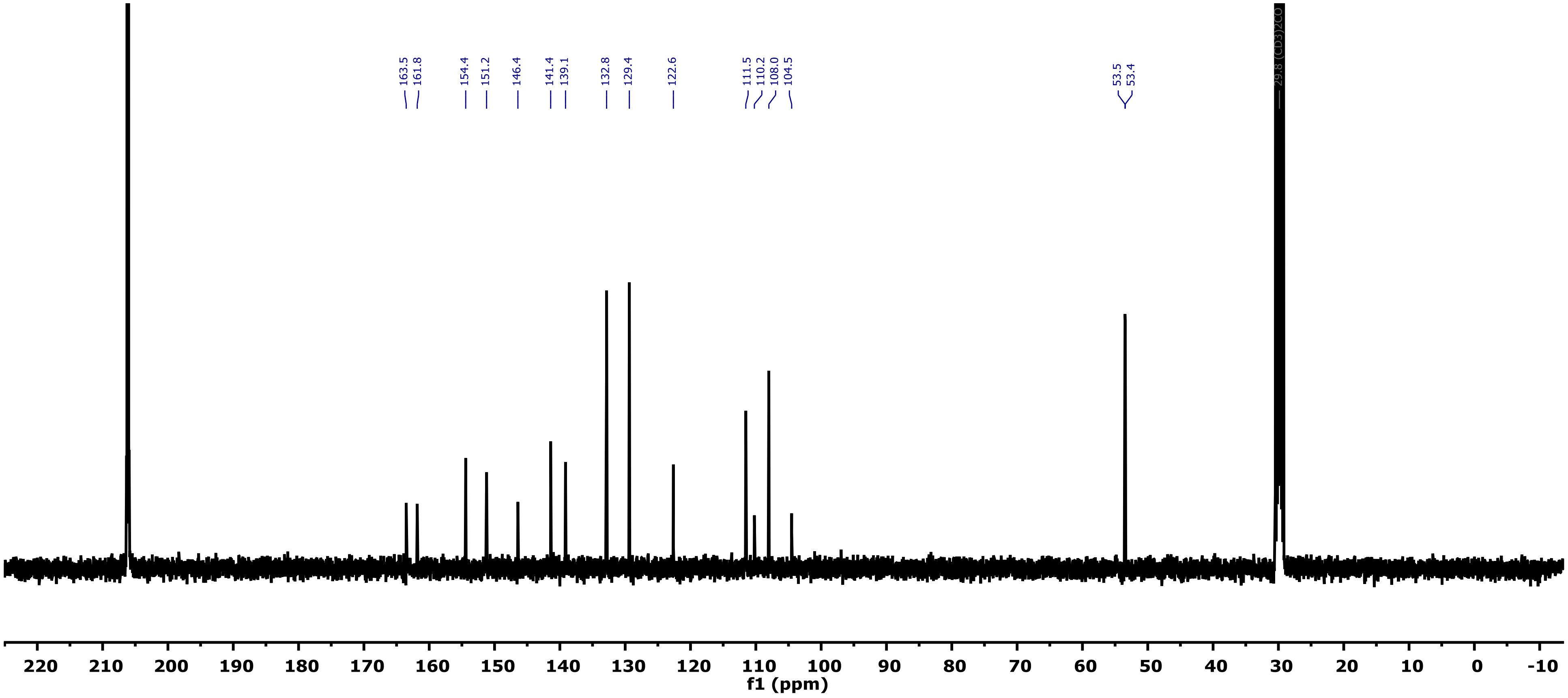

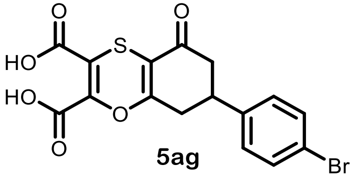

5ag

^{1}H NMR (Acetone d_6, 400 MHz)

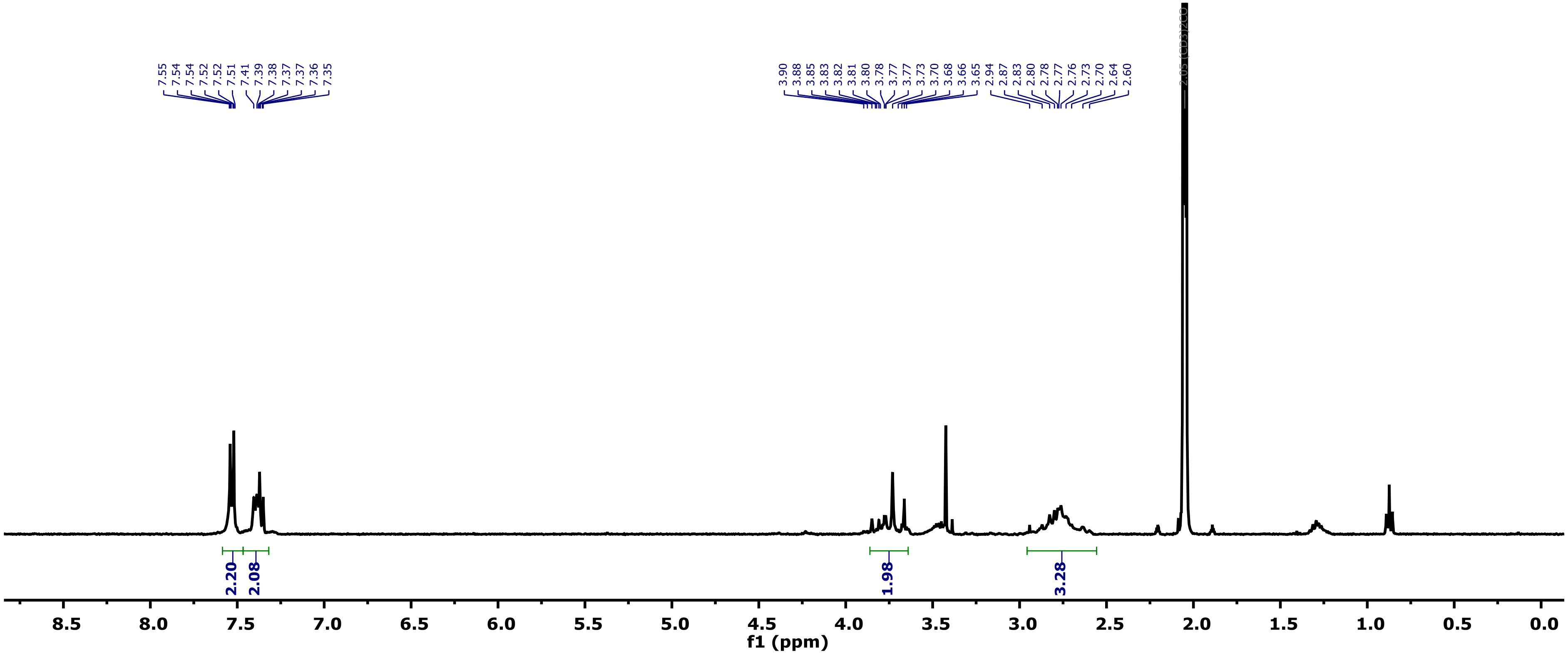

$^{13}C\{H\}$ NMR (Acetone d_6, 101 MHz)

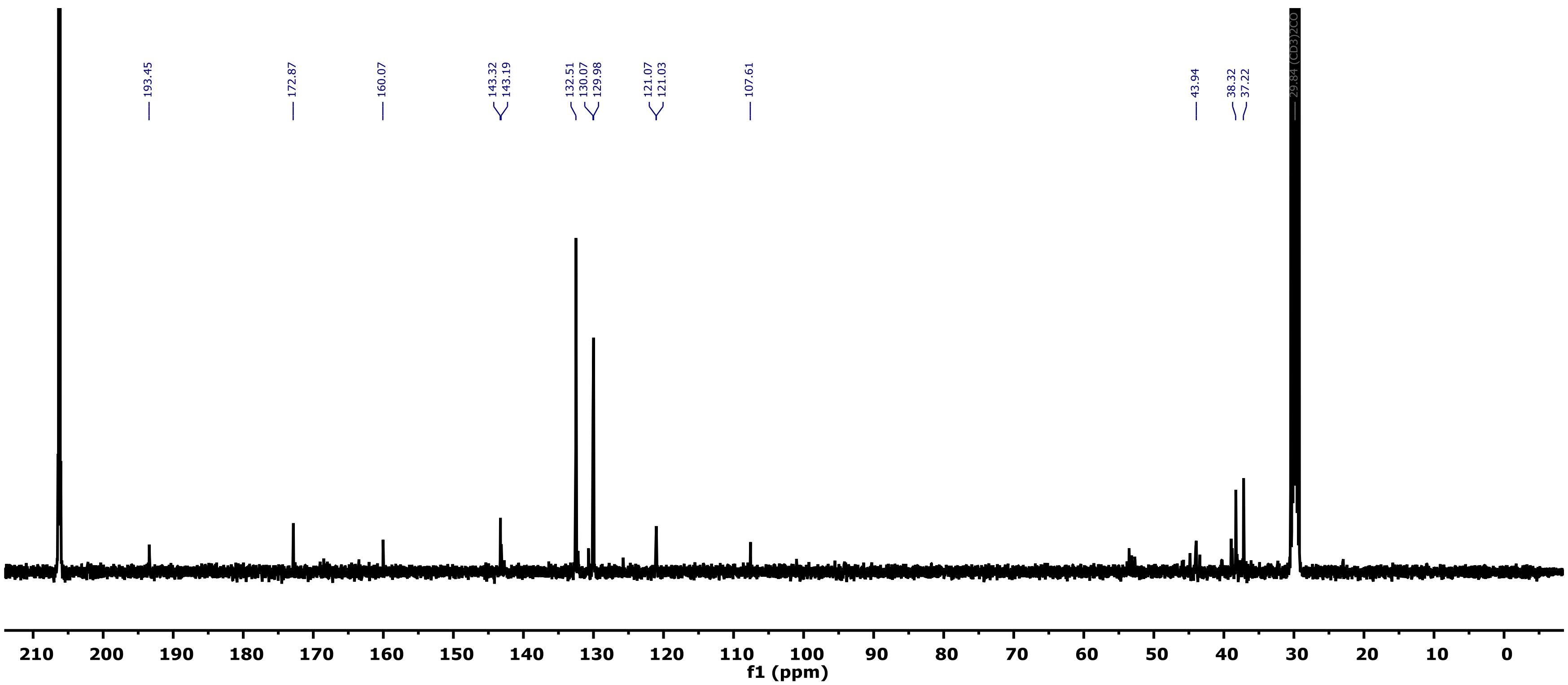

Supplement: Supplementary file 1 — ol3c01538_si_001.pdf [file ol3c01538_si_001.pdf]
